# Discovery of mammalian collagens I and III within ancient poriferan biopolymer spongin

**DOI:** 10.1038/s41467-025-57460-y

**Published:** 2025-03-13

**Authors:** Hermann Ehrlich, Ivan Miksik, Mikhail V. Tsurkan, Paul Simon, Filip Porzucek, Jakub Dalibor Rybka, Monika Mankowska, Roberta Galli, Christine Viehweger, Erica Brendler, Alona Voronkina, Martyna Pajewska-Szmyt, Aleksei Tabachnik, Konstantin R. Tabachnick, Carla Vogt, Marcin Wysokowski, Teofil Jesionowski, Tomasz Buchwald, Miroslaw Szybowicz, Kinga Skieresz-Szewczyk, Hanna Jackowiak, Alexander Ereskovsky, Amadeus C. S. de Alcântara, Alberto M. dos Santos, Clauber H. S. da Costa, Sofia E. Arevalo, Munir S. Skaf, Markus J. Buehler

**Affiliations:** 1 Center for Advanced Technology, Adam Mickiewicz University, Poznan, Poland; 2 Institute of Chemical Technology and Engineering, Faculty of Chemical Technology, Poznan University of Technology, Poznan, Poland; 3 Department of Analytical Chemistry, Faculty of Chemical Technology, University of Pardubice, Pardubice, Czech Republic; 4 Leibniz Institute of Polymer Research Dresden, Dresden, Germany; 5 Max Planck Institute for Chemical Physics of Solids, Dresden, Germany; 6 NanoBioMedical Centre, Adam Mickiewicz University in Poznań, 61-614, Poznań, Poland; 7 Department of Medical Physics and Biomedical Engineering, Faculty of Medicine Carl Gustav Carus, Technische Universität Dresden, Dresden, Germany; 8 Institute of Geology, Chair of Hydrogeology and Hydrochemistry, TU Bergakademie Freiberg, Freiberg, Germany; 9 Institute of Analytical Chemistry, TU Bergakademie Freiberg, Freiberg, Germany; 10 Institute for Nanoscale and Biobased Materials, TU Bergakademie Freiberg, Freiberg, Germany; 11 Department of Pharmacy, National Pirogov Memorial Medical University Vinnytsya, Vinnytsia, Ukraine; 12 Department of Marine Biology, Leon H. Charney School of Marine Sciences, University of Haifa, Haifa, Israel; 13 International Institute of Biomineralogy GmbH, Freiberg, Germany; 14 Institute of Materials Research and Quantum Engineering, Faculty of Materials Engineering and Technical Physics, Poznan University of Technology, Poznan, Poland; 15 Department of Histology and Embryology, Poznań University of Life Sciences, Poznan, Poland; 16 Aix Marseille University, Avignon Université, CNRS, IRD, IMBE, Marseille, France; 17 Laboratory for Atomistic and Molecular Mechanics (LAMM), Massachusetts Institute of Technology, Cambridge, MA USA; 18 Department of Computational Mechanics, School of Mechanical Engineering, Universidade Estadual de Campinas (UNICAMP), Sao Paulo, Brazil; 19 Center for Computing in Engineering & Sciences (CCES), Universidade Estadual de Campinas (UNICAMP), Sao Paulo, Brazil; 20 Institute of Chemistry, Universidade Estadual de Campinas (UNICAMP), Sao Paulo, Brazil; 21 Department of Civil and Environmental Engineering, Massachusetts Institute of Technology, Cambridge, MA USA; 22 Center for Computational Science and Engineering, Schwarzman College of Computing, Massachusetts Institute of Technology, Cambridge, MA USA

**Keywords:** Molecular modelling, Solid-state NMR, Proteins, Proteomics, Natural products

## Abstract

Spongin is a fundamental biopolymer that has played a crucial role in the skeletogenesis of keratosan sponges for over 800 million years. This biomaterial had so far remained chemically unidentified and believed to be an enigmatic type of halogenated collagen-keratin-based bioelastomer. Here we show collagen I and III as the main structural components of spongin. Proteomics, ^13^C solid state NMR and Raman spectroscopy confirm the identity of collagenous domains in spongin with collagen from mammals. Using an HPLC-MS analysis, we found halogenated di- and tri-tyrosines as crosslinking agents in spongin. Using molecular dynamics modeling, we solvated the crystal structures of collagen mimetic peptides for type I and type III collagens in four different systems, including selected brominated crosslinks. The results underscore the complex interplay between the collagen structures and crosslinks, raising intriguing questions about the molecular mechanisms underlying collagen chemistry within spongin as an ancient biocomposite.

## Abstract (web summary)

Scientists find that spongin, a key biomaterial in sponges, contains the same collagens as mammals and that these biocomposites contain brominated crosslinks. Now, the question: “Maybe we are all sponge to some degree?” is no longer so absurd.

## Introduction

Sponges (Porifera) are among the oldest metazoans, established in the Late Proterozoic^1^. These ancient, exclusively aquatic and filter-feeding organisms survived over millions of years of evolution due to their ability to produce biomineralized, mechanically robust three-dimensional (3D) skeletal constructs and to synthesize a broad variety of secondary metabolites with antibiotic and cytotoxic properties^2^. In other words, sponges diverged early in the evolutionary history of multicellular animals, close to the origin of all other metazoans. Not surprisingly, fundamental structural biopolymers such as the amino-polysaccharide chitin^3^ and structural proteins such as actin^4^ and collagens^5–7^ have been found within the skeletal formations of these organisms. The first of these exists in all multicellular organisms up to fish and amphibians^8^, and the other two up to higher mammals, including humans (see refs. ^9,10^ for an overview).

The class Demospongiae, which includes more than 7500 species, contains the oldest fossilized metazoans reported today, at 890 MYR^1^. The mechanically robust and simultaneously elastic 3D skeleton of sponges is made of spongin, one of the main enigmatic structural biopolymers, whose nature has still not been deciphered since its discovery in 1655^11^. Numerous attempts in over 350 years of research to define the makeup of this biopolymer have resulted in a rather complex mosaic picture in which individual pieces are represented by something like collagen and keratin additionally halogenated with bromine, iodine, and chlorine and partially biomineralized with traces of silica and calcium carbonates^3,6,11–13^. Spongin compositions have been found to be rather complex partially due to the presence of xylose^14^, a sugar occurring mostly in plants. In our opinion, the reasons for these unsuccessful attempts were erroneous methodological approaches and misconceptions about spongin as collagen or as keratin. Neither collagenases, keratinases, nor other proteolytic enzymes are able to dissolve spongin^11^. In contrast, some sponginolytic marine microorganisms have been reported previously^15^. However, the possibility is not to be excluded that these microorganisms initially destroy spongin due to the biosynthesis of corresponding “dehalogenases,” enzymes that are still not available on the modern biochemicals market and consequently are not used in research.

The situation surrounding the clarification of the origin of spongin was further complicated by a publication by Fiedler et al.^16^, in which spongins were defined as a family of collagen IV-related proteins composed of a short collagenous domain attached to an NC1 domain. Interestingly, a comparison of collagen IV sequences revealed four cysteine residues absent in spongin sequences, however, the spongin variants do show the conservation of three cysteines absent in collagen IV sequences^16^. Intriguingly, the sulfur content in spongin reaches 5%, which is similar to the content in keratins^17^ and confirms the possible existence of keratin-like domains^18^ within this biopolymer.

We recently reported the identification of a structural triple-helical motif typical for collagen^9^ in spongin fibers of our model demosponge *Hippospongia communis*, using high-resolution electron microscopy (HR-TEM) with a resolution level of 2 nm^3,12^. However, we did not obtain evidence as to which of 29 known collagen types^10^ are related to the nanofibrillar collagen within spongin microfibers (see also Supplementary Note 1).

Thus, our ultimate goal is to obtain fundamental knowledge about spongin’s chemical and material nature as a natural biocomposite with still unknown scientific definitions. Our working hypothesis assumes the discovery of chemical crosslinks, which most likely chemically bind individual collagen triple helices located within the spongin microfibers. We also assumed that a very high concentration of chlorine, iodine, and bromine ions (from 1 to 6%) in spongin^11^ contributed to the particular chemical structure of such crosslinks. In view of the long history of challenges in identifying the composition of spongin, the approach reported here required us to utilize a deep integration of experimental design, theory and modeling.

## Results

### Discovery of collagen within spongin from the *Hippospongia communis* demosponge

**Fig. 1:**
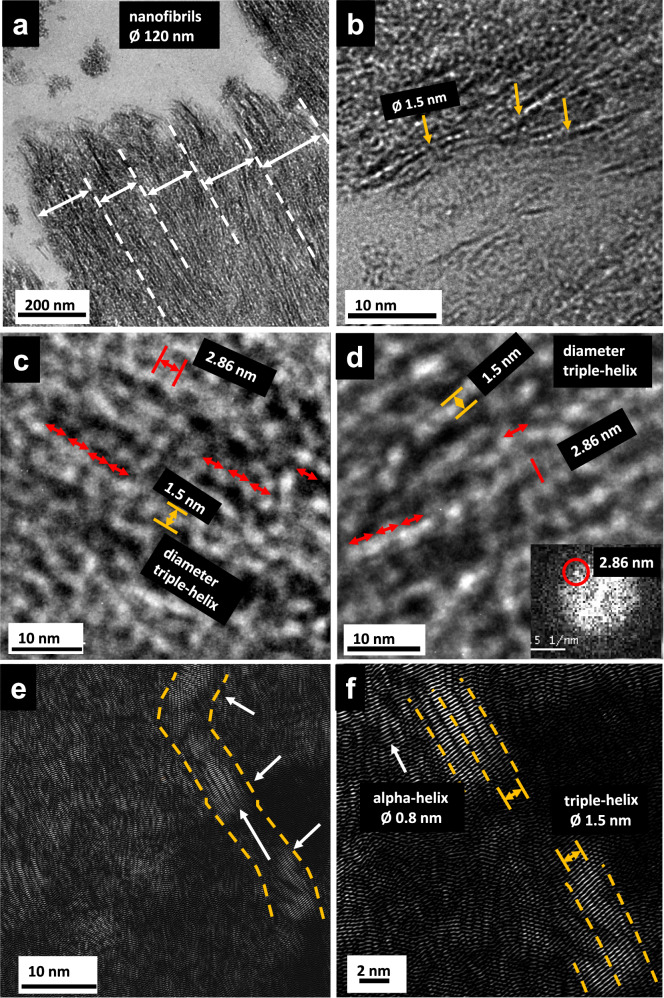
TEM micrographs of ultramicrotomy of non-stained, naturally occurring spongin fiber of *Hippospongia communis* demosponge origin. **a** Assembly of about 120 nm diameter nanofibrils as typically found in collagen type I. **b** Zoom displays individual triple helices (yellow arrows). **c**, **d** Further enlargement reveals a triple helix of 1.5 nm diameter of collagen type I with a characteristic helix periodicity of 2.86 nm along the chain (red arrows). **d** Inset Fast Fourier transform (FFT) of the zoomed region with discrete reflection (red circle) indicating 2.86 nm spacing. **e** Fourier-filtered high-resolution displays a collagen type I fibril (orange lines). **f** Collagen triple helices (white arrows) with 1.5 nm diameter within the spongin are visualized (orange lines). Also, their individual subunits, the so-called alpha chains with 0.85 nm diameter, are resolved (white arrow) (see Supplementary Figs. 2 and 3). The measured spacings were confirmed by repeating the measurements at least at 3 different regions of the sample.

We note that, according to widely established opinion, collagen genes are ancient^19^ and that “*fibril-forming (fibrillar) collagens are extracellular matrix proteins conserved in all multicellular animals. Fibrillar collagens share a single common ancestor that arose at the very dawn of the metazoan world”*^9,20^. Second Harmonic Generation (SHG) imaging is a well-accepted method for label-free visualization of fibrillar collagen^21^. SHG images, presented in the Supplementary Information (see Supplementary Fig. 1) confirms fibrillar collagens in spongin and in collagen samples extracted from spongin. Furthermore, using HR-TEM, we detected the presence of nanofibrils (Fig. 1) within non-stained *H. communis* spongin microfibers with a diameter of 100 µm sequentially treated with 3 M HCl for decalcification and with 10% HF for desilicification.

The nanostructural parameters of these nanofibrils correspond nearly identically to those observed using the same method for collagen I in rat tibia (for details, see ref. ^22^ and Supplementary Figs. 2 and 3).

### Solid state ^13^C NMR, FTIR, and Raman spectroscopy identified collagen within spongin fibers

**Fig. 2:**
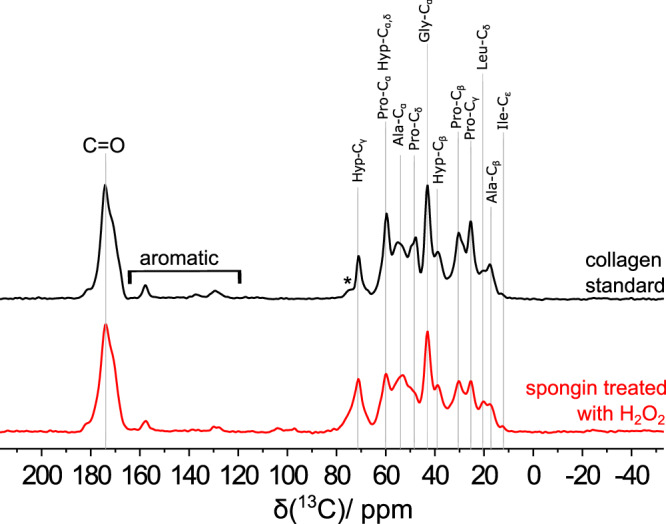
^13^C-CP-MAS NMR spectra of collagen. I standard (black line) and a spongin sample treated with hydrogen peroxide (red line) at a spinning speed of 10 kHz. Asterisks mark spinning sidebands (see also Supplementary Fig. 4). Source data are provided as a Source Data file.

To better understand the spongin structure, we compared the ^13^C cross-polarization (CP) NMR spectra of a collagen type I standard and selected spongin samples measured in natural abundance using the magic angle spinning (MAS) technique (Fig. 2).

In particular, the ^13^C-CP-MAS NMR spectrum of the collagen type I standard (Fig. 2) shows ^13^C signals in a range of 200 ppm to 0 ppm, where especially the region between 80 ppm and 0 ppm, and the broad carbonyl carbon signal at 173 ppm, can be clearly assigned to known chemical shift data of type I collagen^23^. The corresponding dominant amino acids are proline (Pro), alanine (Ala), glycine (Gly), and hydroxyproline (Hyp), and the Greek index refers to the position of the carbon atom within the amino acid. Furthermore, there are ^13^C-signals in the shift range for aromatic carbon atoms in the NMR spectrum of the collagen standard material. The important outcome of the comparison of the ^13^C-CP-MAS NMR spectrum of the spongin sample (Fig. 2, red line) and the assigned spectrum of the type I collagen standard (black line) is the similarity of both. This clear correspondence shows the structural conformity between the spongin sample and the type I collagen. The differences in signal intensities are likely due to differences in amino acid abundance in the two biomaterials. The larger full widths at half maximum (FWHM) in the spongin sample correspond with a less fine ordering of the structures and may also be due to partial hydrolysis of the material^23^.

Furthermore, treatment tests with potassium hydroxide and hydrogen peroxide (for details see “Methods,” Supplementary Fig. 4) show that the spongin structure is clearly degenerated by potassium hydroxide (loss of signal intensity and increase of FWHM), whereas hydrogen peroxide shows only small effects, especially on the assigned proline ^13^C signals, together with an increase in resolution resulting in a spectrum which is thus even more similar to the standard^23^.

Fourier transform IR (FTIR) spectroscopy is a nondestructive technique widely applied for the structural characterization of proteins and polypeptides, including collagens of diverse origin. Especially the amide I band serves as a crucial marker in FTIR analysis of collagen type identification due to the particular vibrational modes and corresponding peak positions^24^.

In this study, we compared the FTIR spectra of type I and III collagen standards with the spectra recorded for native spongin as well as spongin treated with KOH and H_2_O_2_ (see Supplementary Fig. 5). A visible difference between the IR spectra of collagen type I and type III is that the spectrum for collagen type I has distinctive peaks at 1720 cm^−^^1^ and 1199 cm^−^^1^^25^. Comparative analysis of IR spectra obtained for native spongin and KOH- and H_2_O_2_-treated spongin show remarkable similarity with the spectrum of the collagen type III standard (Supplementary Fig. 5).

In Raman spectroscopy, the different types of collagens share similar spectral profiles, substantially displaying the same Raman bands. However, there are a few differences that enable us to distinguish between the collagen types to some extent. It is known that collagen types I and IV can be distinguished based on the relative intensities of phenylalanine bands at 1003 and 1033 cm^−^^1^^26,27^. For collagen type I, the intensity at 1003 cm^−^^1^ is slightly lower than or similar to that at 1033 cm^−^^1^, while for collagen type IV, the intensity at 1003 cm^−^^1^ is much higher than at 1033 cm^−^^1^. This characteristic is clearly visible in the reference spectrum of whale collagen (type I) in Supplementary Fig. 6a and in the spectra of human collagens in Supplementary Fig. 6b (see Supplementary Information). Moreover, it is shared by human collagen types I and III in comparison to type IV.

In the spectra of collagen I standard treated with H_2_O_2_ and all spongin samples, the phenylalanine band at 1003 cm^−^^1^ is weaker than that at 1033 cm^−^^1^. This shows that these samples are constituted by collagen type I and/or III. The same does not apply to the sample COL-IL-H_2_O (see Supplementary Fig. 6a), which displays a strong band at 1003 cm^−^^1^ and additionally several band shifts compared with the collagen standards.

Additional differences reported between collagen types I and IV^26^ involve the bands at 814 cm^−^^1^ (stronger in collagen type I) and 940 cm^−^^1^ (stronger in collagen type IV), and changes in the form of the two overlapping bands at 1243 and 1270 cm^−^^1^ (the latter being less defined in the spectrum of collagen type IV).

**Fig. 3:**
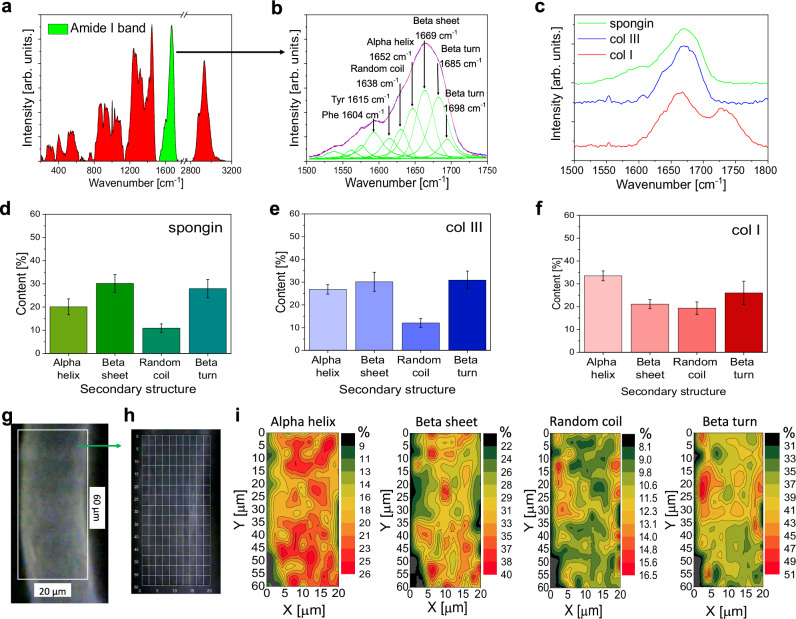
Raman spectroscopy of individual spongin fiber. **a** Raman spectrum of spongin fiber with analyzed amide I band marked in green. **b** Deconvolution of amide I band in Raman spectra of spongin. The individual bands are marked in green, while the red band represents the sum of all the individual bands. **c** Amide I band of spongin in comparison to reference samples of Col I and Col III. Percentage amount of secondary structure of proteins in spongin (**d**), Col I (**e**), and Col III (**f**). Data are presented as mean values. The mean value was calculated from *n* = 165 data points, with error bars representing the standard deviation. **g** Camera image of the spongin fiber subjected to analysis. **h** Raman spectra measurement grid. **i** Percentage distribution of alpha helix, beta sheet, beta turn and random coil conformations in spongin. Source data are provided as a Source Data file.

A Raman spectrum of the spongin studied here, with the amide I band marked in green, is shown in Fig. 3a. Therein, the curve-fitting process for the amide I band was used to determine the percentage content of different types of secondary protein structures, such as alpha helix, beta sheet, and beta turn (Fig. 3b)^28–30^. The shape of the amide I band of spongin, compared with that of collagen I and collagen III (Fig. 3c), as well as the percentage content of particular secondary structures (Fig. 3d–f), indicates the presence of collagen type III in spongin. Raman maps were used to present the spatial changes in the relative quantity of secondary protein structures. In Fig. 3g, the selected spongin fiber subjected to analysis is shown. Additionally, the Raman spectra measurement grid is presented in Fig. 3h. The percentage distribution of proteins in spongin in alpha helix, beta sheet, beta turn, and random coil conformations^31,32^ is shown in Fig. 3i.

Thus, the obtained analytical data confirmed the presence of both collagen type I and III, similar to those of mammalian origin, within selected *H. communis* spongin samples.

### Proteomics reveals collagens I and III as components of spongin fibers

The experimental data provides evidence for the existence of both collagens (types I and III) as the main structural proteinaceous components within the studied purified fibers of spongin. To confirm this, we analyzed selected extracts from purified spongin isolated from *H. communis* demosponge skeleton using complementary proteomic approaches with SDS-PAGE (Supplementary Figs. 7–9) and LC separation, followed by mass spectrometry and western blot analysis (see “Detailed protein report” in Supplementary data). Proteomics involves the application of various techniques to identify and characterize proteins, including the specific collagen chains present in diverse species of invertebrates and vertebrates (see ref. ^33^ for an overview).

**Fig. 4:**
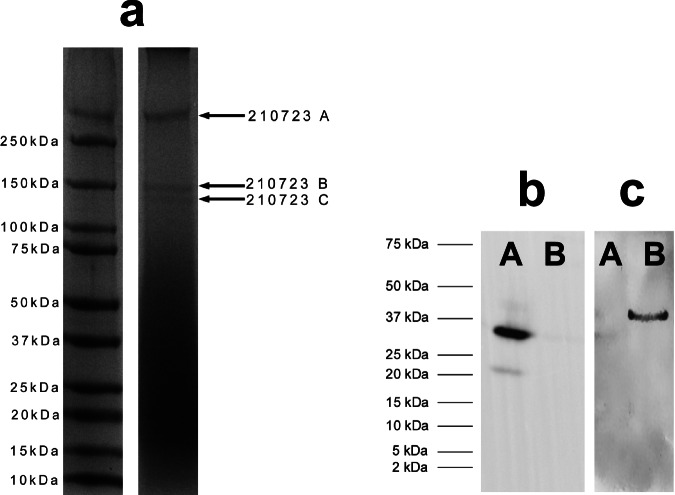
Identification of collagens within spongin fibers of *H. communis* marine demosponge. **a** SDS-PAGE analysis of spongin extracted by method C (see “Methods” and Supplementary Figs. 7–9) indicating the presence of three collagen chains (210723A, 210723B, 210723C). **b** Identification of collagens I and III in this spongin with Western Blot—two bands present. **c** Identification of Collagen III in the spongin with Western Blot—one band detected. Sample A—disintegrated spongin (pH 7.1); Sample B—disintegrated spongin (pH 2.3). Both the primary antibodies (Collagen I and III) as well as HRP-conjugated anti-rabbit secondary antibodies were used at dilution 1:1000 (see “Methods”). Merged results for proteins identified by LC-MS/MS in specific bands are given in Table 1. Each investigation was repeated 3 times. Source data are provided as a Source Data file.

SDS-PAGE analysis of spongin extracted by method C indicated the presence of three collagen bands in the sample (Fig. 4). LC-MS/MS analysis proved the presence of collagen I alpha-1 chain isoform X1 and collagen I alpha-2 chain in band A, collagen I alpha-1 chain isoform and collagen III alpha-1 chain precursor in band B, and collagen I alpha-2 chain precursor and collagen I alpha-1 chain isoform in band C (Fig. 4, Table 1, Supplementary Data). The detailed proteomic profile of bands identified within spongin is presented in “Detailed protein report” in Supplementary data.

**Table 1:** Proteins identified in spongin by LC-MS/MS

SDS-PAGE Band	Accession	Protein	MW (kDa)	Scores	#Peptides	SC (%)
210723 A	XP_020922812.1	Collagen alpha−1(I) chain isoform X1 [Sus scrofa]	139.20	1557.46	23	23.70
210723 A	BAX02569.1	Alpha 2 chain of type I collagen [Sus scrofa domesticus]	129.10	640.97	9	9.80
210723 B	XP_020922812.1	Collagen alpha−1 (I) chain isoform X1 [Sus scrofa]	139.20	6511.94	112	50.20
210723 B	NP_001230226.1	Collagen alpha−1 (III) chain precursor [Sus scrofa]	138.50	1677.83	33	24.50
210723 C	NP_001230584.1	Collagen alpha-2(I) chain precursor [Sus scrofa]	129.10	4409.82	79	50.40
210723 C	XP_020922812.1	Collagen alpha−1(I) chain isoform X1 [Sus scrofa]	139.20	3449.69	66	45.70

(SDS-PAGE BAND—the band excised from the gel (Fig. 4) and subjected to analysis; Accession—database accession number of identified protein; Protein—name of the identified protein; MW—molecular weight of the native protein, as provided by theoretical database data; Scores—a statistical score indicating the probability of peptide or protein matching to the theoretical sequence in the database; Peptides—number of peptides derived from the analyzed protein and mapped to the theoretical sequence; SC (%)—Sequence Coverage—percentage of the protein sequence covered by the identified peptides). For Detailed protein reports, see Supplementary Data.

For spongin from the *H. communis* sponge, the presence of collagens I and III was additionally confirmed by western blot using specific primary antibodies (Fig. 4; see also “Methods”). This method has previously been successfully used for the identification of vertebrate collagen V in the fibrous tissue matrix of the sea-pen *Veretillum cynomorium*^34^. Thus, the selected molecular biological techniques used in the study unambiguously confirmed the presence of both collagen types I and III within *H. communis* spongin fibers.

Additionally, a search was made for genomes from six demosponge species (*Amphimedon queenslandica*, *Aplysina aerophoba*, *Chondrosia reniformis*, *Ephydatia muelleri*, *Halichondria panicea*, *Petrosia ficiformis*) and one homoscleromorph (*Oscarella lobularis*) using the BLAST-2.15.0+ toolkit (see Supplementary Note 2, Supplementary Fig. 10, Supplementary Table 2) in order to identify sequences similar to the collagen-related proteins identified in the spongin under study by LC-MS/MS or the nucleotide sequences encoding them (see Table 1, Fig. 4). Translated nucleotide BLAST was able to identify several sequences in the poriferan genomes with significant similarity to the same type and chain of collagen as input from the collagen of wild pig (*Sus scrofa*) (see Supplementary Note 2, Supplementary Table 2).

### Discovery of di-and tri-Br-tyrosines within the spongin matrix

Amino acid (AA) analysis of spongin has been described in several reports (see for overview Supplementary Table 3). An interesting observation in AA was a surprisingly high amount of tyrosine residues (3–5%), which is not common for collagens (0.5–1.0% tyrosines). Moreover, tyrosine in spongin is mostly (>90%) halogenated as mono- and di-bromine derivatives (which was also previously confirmed by CMXRF measurements of spongin, where the even distribution of bromine through the 3D matrix of the biopolymer is clearly visible; see Supplementary Fig. 11, Supplementary Note 3) with a smaller amount of chlorine and iodine derivatives^11^. Intriguingly, it was reported that a significant amount of the tyrosine residues consists of ortho-tyrosine, which, in nature, is a product of non-specific phenylalanine oxidation only^35^. Therefore, the source of the tyrosine excess in spongin is the oxidation of phenylalanine residue, which is well presented in collagen proteins (3–5%) but is almost missing in the spongin. This makes sense, as spongin represents an extracellular skeletal structure of bath sponges, which, although covered by mesohyl, still indirectly contacts the marine environment and is constantly exposed to oxidative and halogenated stresses (the concentration of bromine in seawater is about 65 mg/L^36^). The oxidation of phenylalanine to tyrosine could also lead to the formation of dityrosines, but this has never been observed in the AA analysis of spongin. However, the presence of di- and tri-tyrosines would explain spongin’s specific mechanical stability and robustness.

**Fig. 5:**
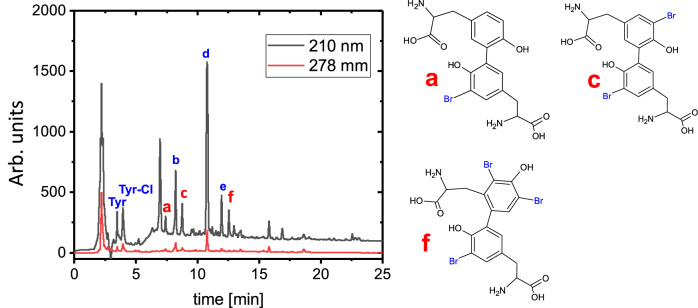
Reverse phase C−*1*8 HPLC chromatogram of spongin acid hydrolysis. The chromatogram peaks labeled with blue letters correspond to known halogenated derivatives of tyrosine. Those with red letters correspond to brominated dityrosines, never previously reported in sponges. The structure of the main brominated dityrosines found in the sponge is shown on the left. A detailed ESI-MS and UV-Vis analysis of each highlighted compound is given in Supplementary Information, Supplementary Figs. 12−18. Source data are provided as a Source Data file.

We hypothesized that the collagen fraction of spongin is crosslinked by halogenated dityrosines, which may escape the common AA analysis due to their high hydrophobicity. To check our hypothesis, we analyzed spongin samples fully hydrolyzed with HCL with an HPLC-MS analysis optimized for terpenes (see Supplementary Figs. 12–20). Our analysis reveals that tyrosine derivatives can be easily separated from other amino acids (Fig. 5). The main tyrosine moieties of spongin were mono-chlorotyrosine, bromotyrosine and dibromotyrosine, consistent with the previously reported analyses. However, there was also revealed a significant presence of dibromo-dityrosine, the previously unreported tribromo-dityrosine, and their unknown iodo- and chloro-derivatives. Our analysis revealed that overall, about 10–15% of all tyrosine moieties in spongin exist as halogenated dityrosines. Some of them, including 5-bromo-3,3’-dityrosine, 5,5′-dibromo-3,3’-dityrosine, and 4,6,5′-tribromo-2,3’-dityrosine (see Supplementary Figs. 13, 15, 18) have not been previously reported in spongin. To the best of our knowledge, among the discovered halogenated di- and tri-tyrosines, only 3-bromo-dityrosine, 3-3’-dibromo dityrosine and 3-bromo-trityrosine have been reported, each on a single occasion, for example, in the cuticle of common brown crab (*Cancer pagurus*) as a side product of a sclerotization process^37^ without obvious evolutionary benefit for the species. In contrast, the halogenated di- and tri-tyrosines in spongin are at least partly the reason for the superior stability and elasticity of spongin as found only in bath sponges^38^.

The elastic modulus of spongin from bath sponges ranges from 121.8 to 838.7 kPa^38^, while for naturally hydrated resilin, a di- and tri-tyrosine-crosslinked bioelastomer, this value is reported as 600–700 kPa^39^. The possible role of the di- and tri-bromotyrosines in shaping the mechanical properties of spongin as a bioelastomer of poriferan origin remains an open question and should be studied in detail.

This discovery clearly explains why the protein component of spongin was never identified previously, as di-tyrosine crosslinking prevents its analysis by common protein analytical methods. On the other hand, di-tyrosine in sponge exists exclusively in halogenated forms, which could not be revealed in common AA methods of analysis.

Di-tyrosines are less common in crosslinking proteins, which are formed by active oxidative moieties’ interaction with two tyrosines that are sterically close to each other. Similarly to collagen I–III, spongin has a fibrous triple helical-based structure, thus, the tyrosines from different tropocollagens can be sterically close to each other. Its oxidation will lead to the formation of intermolecular crosslinking, which cannot be reduced. Phenylalanine does not form dimers, therefore, the formation of the di-tyrosines can occur at any stage following the first oxidation of the phenylalanine to tyrosine, as shown in Supplementary Figs. 19, 20. Only meta-tyrosine can form tri-bromo-di-tyrosines (Fig. 5f), revealing a significant presence of meta-tyrosine and proving the oxidative (not biosynthetic) nature of most tyrosine residues in spongin.

### Molecular dynamics investigations

**Fig. 6:**
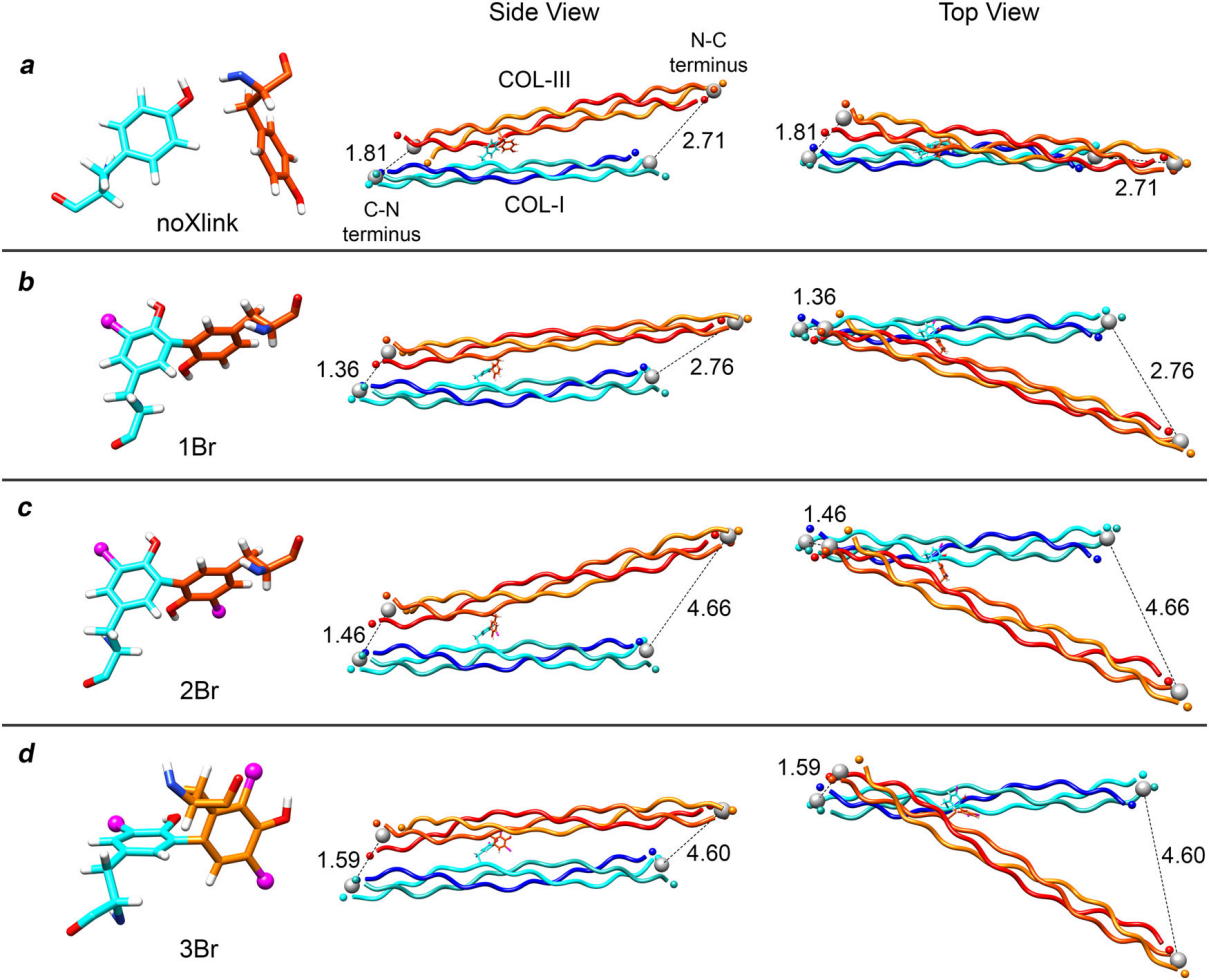
Molecular models of crosslinked collagens type I and III. Each row displays a different system: **a** noXlink, **b** 1Br, **c** 2Br, and **d** 3Br. The column on the left shows 3D visualizations of the crosslinks (bromide atoms in magenta). The middle and right columns show the side and top view, respectively, of measured terminal distances (nm) at the C-N and N-C ends (left and right terminals of the molecules, respectively). COL-I is displayed in blue/cyan, and COL-III in orange/red.

To further understand the impacts and possible mechanisms underlying the bromide crosslinks shown in Fig. 5, we explored simplified all-atom models of spongin by means of molecular dynamics (see Fig. 6). We solvated the crystal structures of collagen mimetic peptides (CMP) for type I (COL-I) and type III (COL-III) collagens in water, modeling four different systems: three crosslinks (1Br, 2Br, and 3Br crosslinks based on the structures described in Fig. 5 a, c and f, respectively) and one without a crosslink (noXlink). All systems were simulated for 200 ns. Additional details regarding our models and simulations are provided in Supplementary Note 4 and in the “Methods” section.

We calculated various parameters, such as RMSD, radius of gyration, collagen width, and terminal distances, to investigate the effects of different crosslinks on our models (see Supplementary Note 4, Supplementary Fig. 21, Supplementary Tables 4–6). We highlight the behavior of the terminal distances, as shown in Fig. 6. Our analysis shows that the crosslinks affect both terminal ends. When no crosslink is present (noXlink), the C-N distance is relatively large, and the N-C distances are smaller. However, as we introduce crosslinks (1Br, 2Br, and 3Br), the C-N distances decrease and the N-C distances increase. This observation is particularly intriguing, as it provides insights into how the positioning of the crosslinks may impact the behavior of the collagen’s terminal regions. This result may be related to reported metal-ion-mediated self-assembly in CMPs^40^. The use of both metal-ligand and hydrophobic interactions along the peptide backbone to form higher-order structures offers a compelling parallel to the influence of bromide crosslinks^40^.

Our simulations demonstrate that the modeled crosslinks induce structural changes, affecting the dynamics and flexibility of the collagen molecules. Notably, COL-I and COL-III exhibit different responses to crosslinking, suggesting that their distinct structural properties influence how they interact with bromine atoms^41^. These findings emphasize the complex interplay between the collagen structures and crosslinks, raising intriguing questions about the molecular mechanisms underlying collagen chemistry and mechanics. While our study sheds light on the immediate consequences of crosslinking for such simplified systems (short CMPs instead of fibrillar structures), further investigations are essential to comprehensively understand the molecular impacts of such crosslinks, not only in spongin structures but also in the structure of bones and tendons^42^. This knowledge will advance our understanding of collagen-based biocomposites and enable the development of tailored materials for a wide range of biomedical and engineering applications.

## Discussion

Therefore, our discoveries suggest that the main collagen structural domains (COL-I and COL-III) of mammals, including humans, were already present in the spongin-based skeletons of the first multicellular animals, such as marine keratosan demosponges. In vertebrates, collagen I is responsible for forming a matrix of diverse connective tissues, and collagen III represents a major structural component in such organs as the bowel, uterus, and blood vessels^43^. In light of these findings, it is reasonable to conclude that the presence of these fundamental collagens in sponges reflects a shared evolutionary origin with other animals. Halogenation of the collagen domains that we discovered in spongin, through the formation of specific crosslinks, led to the formation of both mechanically strong and elastic fibers that contributed to the formation of a 3D microporous skeletal structure optimized for water filtering and expressed in the physical form that we call “sponge.” As was recently reported^44^, chloride ions play a crucial role in scaffold assembly and stabilization of the hexamer structure in collagen IV. Whether such halogen-dependent mechanisms of collagen self-assembly during spongin formation are of fundamental importance is still unknown. The possible role of brominated compounds within spongin in the exceptional preservation of this biocomposite in fossilized demosponges^1^ remains an open question. Future work could explore Bromine NMR, leveraging brominated Tyrosine as a spectrally uncrowded probe for studying molecular configurations and dynamics of collagens. Bromine’s two stable isotopes, Br-79 and Br-81, with their distinct yet complementary NMR properties, may offer a novel means to analyze collagen crosslink geometries, molecular dynamics, and identification with high specificity. Also, for the future, LC-MS/MS (with the characteristic isotope patterns of both halogens in the MS) remains the method of choice for the separation and identification of the observed compounds, where possible, supported by 1H, 13C and maybe 15N NMR.

The conserved chemistry of sponge skeletons suggests that this is an ancient role that collagens I and III have played for at least 800 million years. A remarkable finding is that especially nanofibrillar collagen I, having arisen in the skeletons of horny sponges as the first metazoans, is to be found in the spongy (cancellous) bones of mammals. The intriguing question: “*Maybe we are all sponge to some degree?*” is no longer so absurd.

## Methods

### Ethics statement

Dried samples of spongin from the sponge *Hippospongia communis* were obtained in an industrially harvested form intended for cosmetic use and purchased from INTIB GBMH; therefore, their use does not require ethical approval or a permit for animal experimentation according to current legislation.

### Second harmonic generation (SHG) imaging

SHG imaging was performed on a laser scanning microscope with excitation at a wavelength of 780 nm. The laser was focalized with a 20×/NA = 1.00 objective, and the SHG signal was collected in the forward direction. All details about the microscope are reported elsewhere^45^.

### Comparative TEM imaging

Purified, acellular, mineral- and pigment-free skeletal spongin fibers of *Hippospongia communis* demosponge were placed in 30% ethanol for one day at room temperature (RT). They were then dehydrated at RT in an ethanol series (ethanol 30% 2 × 20 min; 50% 2 × 15 min; 70% 3 × 15 min; 95% 3 × 15 min; 100% 3 × 20 min). Araldite inclusion: LMRSOL3® (LaboModderne, France) 2 × 20 min; LMRSOL3®: Araldite epoxy embedding media (Spi Supplies, USA) (1:1) for 1 h; Araldite (Sigma‐Aldrich) 2 × 2 h according to the manufacturer’s instructions. Ultrathin sections (60–80 nm) were cut with an Ultramicrotome PowerTome XL, equipped with a Drukkert 45° diamond knife, and contrasted with UranyLess (EMS) solution and lead citrate. TEM analysis of the ultrathin sections was carried out by the FEI Tecnai F30-G2 transmission electron microscope. The TEM works with Super-Twin lenses (ThermoFisher, Eindhoven, NL) and with a field emission gun at an acceleration voltage of 300 kV. The point resolution amounts to 2.0 Å, and the information limit amounts to about 1.2 Å. The microscope is equipped with a wide-angle slow scan CCD camera (MultiScan, 2k × 2k pixels; Gatan Inc., Pleasanton, CA, USA). The analyses of the TEM images were realized by means of the Digital Micrograph software (Gatan, USA). Selected TEM images (Fig. 1e, f) were processed by Bragg filtering of the Fourier transform of high-resolution images. (For details see Supplementary Figs. 2, 3).

### Solid state ^13^C NMR spectroscopy

Selected spongin samples (see Supplementary Figs. 7–9) for comparative analytics using Solid State ^13^C NMR, FTIR (Supplementary Fig. 5), and Raman (Supplementary Fig. 6, Supplementary Table 1) spectroscopy were treated separately using 10% KOH and 35% H_2_O_2_ for 5 h at room temperature. Samples were then rinsed with deionized water to pH 6.5, dried at 37 °C for 24 h, and used for analytical investigations.

^13^C cross-polarization NMR spectra were recorded on a Bruker Avance III HD 400 MHz WB spectrometer operating at a frequency of 400.30 MHz for ^1^H and 100.67 MHz for ^13^C, using a 4 mm triple resonance VT CP MAS probe (Bruker) and a spinning frequency of 10 kHz. CP experiments were carried out with a 1 ms contact time and a 70% CP ramp. The recycle delay was set to 5 s. Tppm15 decoupling was used during the acquisition time. In each case, 2048 scans were taken. The chemical shifts are reported relative to tetramethylsilane (TMS).

### FTIR spectroscopy

Infrared spectroscopy was used for the characterization of spongin after various treatments as well as collagen type I and type III standards. The presence of the expected functional group was confirmed by ATR–FTIR (attenuated total reflectance–Fourier transform infrared spectroscopy) and verified using a Nicolet 210c spectrometer (Thermo Scientific, Waltham, USA). The samples were analyzed using the ATR system with resolution equals 4 cm ^−1^ (For details see Supplementary Fig. 5).

### Raman spectroscopy of spongin

Raman spectra were recorded using a Raman spectrometer (RamanRxn1, Kaiser Optical Systems Inc., Ann Arbor, USA) coupled to a light microscope (DM2500 P, Leica Microsystems GmbH, Wetzlar, Germany). The excitation was obtained with a diode laser emitting at a wavelength of 785 nm focused on the samples with a 100×/0.75 microscope objective. The spectral resolution was 4 cm^−^^1^.

Spectroscopic data were analyzed and displayed with MATLAB (MathWorks Inc., Natick, USA). A baseline correction was performed with the function “msbackadj” and spectra were then normalized to the maximum intensity of the amide I band.

Reference Raman spectra of human collagen type I, III, and IV were collected from standard materials (p. n. C7774, C4407, and C5533, respectively, purchased from Sigma-Aldrich).

In the analysis of the amide I band in Raman spectra of individual spongin fibers, measurements were carried out using an inVia Renishaw system with a laser emitting at a wavelength of 785 nm, focused on the samples with a 50×/0.75 microscope objective. The spectral data were calibrated using the Raman band at 520.7 cm^−1^ of a silicon internal reference sample. Changes in the secondary structure of proteins were presented using Raman maps. These maps were collected on the surface of the spongin, within a rectangular area of 60 × 20 µm, with a single analysis performed at each data point (steps of 2 µm in the x direction and 4 µm in the y direction) (For details and references see Supplementary Fig. 6, Supplementary Table 1).

### Methods for collagen extraction from spongin

Acellular, mineral- and natural pigment-free grated samples of skeletal spongin isolated from the marine demosponge *H. communis* (Lamarck, 1814) (Supplementary Fig. 7) were used for the extraction of collagens (For details see Supplementary Figs. 8, 9).

#### Method A

In a standard procedure, the IL-assisted isolation of collagen from spongin involved two primary steps: the dissolution of spongin 1-butyl-methylimidazolium acetate and the subsequent regeneration of collagen using a precipitator (propan-1-ol) (Supplementary Fig. 8).

The dissolution stage was performed at an EasyMax 102 station (Mettler Toledo, Switzerland). A 20 ml flask was filled with [BMIM] acetate (10 g) and heated to 50 °C. 25 mg of spongin powder (Supplementary Fig. 7) was added incrementally every 3 h to achieve a final concentration of 1%. Dissolution was performed at 50 °C for 48 h.

The regeneration stage was performed by mixing 10 ml of the obtained spongin/IL solution with 50 ml of cold (4 °C) propan-1-ol and shaking vigorously for 1 min. The decanted precipitate was washed several times with fresh propan-1-ol to remove the ionic liquids. It was then air-dried and used for further analyses (proteomics).

#### Method B

An amount of 2 g of grated spongin was treated with 50 mL of Tris-HCl buffer (pH 7.1), and the content was incubated for 3 days at room temperature (RT) with simultaneous shaking. The aqueous layer was then left for 24 h at 4 °C, followed by obtaining a centrifugation supernatant by adding NaCl to reach a concentration of 2.6 M. The salted supernatant was incubated at 4 °C for 3 days. The procedure was performed for four samples. The precipitate protein was obtained by centrifugation (6000×*g*, at 4 °C, for 1 h), and all four precipitated proteins were combined and stored at −20 °C prior to SDS-PAGE analysis.

#### SDS-PAGE and staining procedures

The precipitated proteins from *H. communis* spongin extract were solubilized using 100 µL of 4x-concentrated protein loading buffer (Roti® Load 1) and mixed on a vortex for 15 min. The extract was then heated at 95 °C for 5 min (on a thermo block). After centrifugation (1680×*g* for 5 min) the supernatant was collected. A 50 µL aliquot of the sample for Coomassie blue stain and 20 µL for silver stain was electrophoresed. Electrophoresis was carried out on polyacrylamide gel: on 4% stacking gel (7.8 mL dH_2_O; 1 mL 1 M Tris-HCl pH 6.8; 1 mL 40% ROTIPHORESE®Gel 40 (37.5:1); 200 µL 20% SDS; 32 µL 30% APS; 16 µL TEMED) and 7.5% resolving gel (7.1 mL dH_2_O; 300 µL glycerine; 5.2 mL 1.5 M Tris-HCl pH 8.8; 3 mL 40% ROTIPHORESE®Gel 40 (37.5:1); 400 µL 20% SDS; 40 µL 30% APS; 16 µL TEMED). The gel was run using Tris-Glycine/SDS running buffer at 90 V (stacking gel) and 160 V (resolving gel). To control protein separation, the marker (Roti® Mark Tricolor) was simultaneously run on SDS-PAGE with the samples. After electrophoresis, the gel was divided into two parts: one was stained with Colloidal Coomassie CBBG-250, and the second was used for silver staining using Roti® Black-NSeq kit.

After the electrophoresis, the gel was placed in a 100 mL fixing solution (79 mL H_2_O + 1 mL phosphoric acid 85% + 20 mL MeOH) for 3 h. After this time, Roti® Blue solution (20 mL) was mixed with 60 mL H_2_O and 20 mL MeOH, and the gel was incubated in Roti® Blue solution overnight. The gel was destained with a mixture of 25 mL MeOH and 75 mL distilled H_2_O. This step was repeated three times. Finally, the gel was placed in 100 mL of drying solution (10 mL glycerin, 20 mL EtOH, 70 mL H_2_O).

After electrophoresis, a Roti® Black NSeq silver staining kit (L533.1/2) was used for staining following the procedure supplied with the kit.

#### Method C

Spongin was cut into small pieces, inserted into a grinding vial, and pre-cooled in liquid nitrogen. After 10 min, chilled samples were pulverized in a cryogenic laboratory mill (6875 Freezer/Mill, SPEX) with a magnetically driven impactor while continuously immersed in liquid nitrogen. Grinding time was three cycles of cryo-milling: 3 min per grinding cycle at 15 cps, with a 2-min intercool.

The pulverized spongin was subjected to an extraction process with the use of 0.1 M lactic acid (10 g of spongin powder + 100 mL of lactic acid) for 12 h at RT on a magnetic stirrer. The resulting suspension was centrifuged, the precipitate was discarded, and the supernatant underwent a filtration process on a syringe filter (0.8 μm). Subsequently, the filtrate was concentrated 10 times on Amicon Ultra 50 MWCO 100 kDa centrifugal filters (Millipore).

Next, proteins were separated in 1D protein electrophoresis with the use of 4–20% gradient Mini-PROTEAN® Precast Gel (Bio-rad) for 15 min at 300 V. The gel was stained with Coomassie Brilliant Blue G solution. The visualized bands of interest (see Fig. 4 in the main text) were excised with a scalpel, frozen separately, and used for proteomics (see Supplementary data for Detailed protein report).

### Proteomic analysis

#### Analysis of tryptic digests with LC-MS/MS

The protein digest was analyzed by the nano-HPLC apparatus Proxeon Easy-nLC (Proxeon, Odense, Denmark) coupled by a nanoelectrosprayer to ultrahigh-resolution quadrupole-time of flight mass spectrometer MaXis Q-TOF (Bruker Daltonics, Bremen, Germany). The software packages used for controlling the instruments were HyStar 3.2 and micrOTOF-control 3.0, and ProteinScape 3.0 and DataAnalysis 4.0 for data collection and manipulation (Bruker Daltonics).

The samples (5 μL) were injected into precolumn (trap column) NS-MP-10 Biosphere C18 (particle size: 5 μm, pore size: 12 nm, length: 20 mm, inner diameter: 100 μm) and column NS-AC-12dp3-C18 Biosphere C18 (particle size: 3 μm, pore size: 12 nm, length: 200 mm, inner diameter: 75 μm) both prepared by NanoSeparations (Nieuwkoop, Holland).

The separation was done by a linear gradient between water (phase A) and acetonitrile (phase B) both containing 0.1% (v/v) formic acid. The elution started by mobile phase consists with 5% B, next 5 min followed by a gradient elution to 7% B, after by gradient elution 30% B at 180 min. Next 10 min the column was eluted by a gradient to 50% B, and the last 10 min eluted by a gradient to 100% B. Finally, the column was washed with 100% B for 20 min. The column was equilibrated between runs by 5% B for 10 min. The temperature of separation was an ambient temperature (25 °C) when the flow rate was 0.20 μL/min.

On-line nano-electrospray ionization (easy nano-ESI) was used in positive mode. The ESI voltage was set to +4.5 kV, scan time 3 Hz. The drying gas was nitrogen: flow rate 4 L/min and temperature 180 °C; The nebulizer pressure was set as 100 kPa. The masses were scanned in the range from 50 to 2200 m/z. As the internal mass lock was used a monocharged ion of C_24_H_19_F_36_N_3_O_6_P_3_ (m/z 1221.9906). To enable an accurate molecular mass determination the mass spectra corresponding to each signal from the total ion current chromatogram were averaged.

#### Database searching

The software used for data processing was ProteinScape software v. 3.0.0.446 (Bruker Daltonics, Bremen, Germany). The database for protein identification was extracted for collagen from the NCBI database (downloaded on July 4, 2020; 133,126 sequences; 105,045,692 residues) using the MASCOT search engine v. 2.3.0 (http://www.matrixscience.com). The searching on the SwissProt database (downloaded on July 4, 2020; 562,755 sequences; 202,599,198 residues) was used as a control. The setup for all these searches was: trypsin as the enzyme, three missed cleavages, mass tolerance for MS was ±10.0 ppm and ±0.03 Da for MS/MS analysis. There were selected variable modifications: hydroxylation of proline and lysine, oxidation of methionine, and deamidation of asparagine and glutamine. The monoisotopic peptide charge was set to 1+, 2+ and 3+. To remove false positive results the Peptide Decoy option was set up. For the determination of significant hits, the MASCOT score ≥80 for proteins and ≥20 for peptides was selected. In addition, all peptides and proteins were manually validated.

### Western blot analysis

#### Western blot of low molecular weight proteins (<100 kDa)

The proteins were transferred (20 min; 6 mA/1 cm^2^) with the specific transfer buffers (anode buffer: 60 mM Tris, 40 mM CAPS, pH 9.6 15% MeOH; cathode Buffer: 60 mM Tris, 40 mM CAPS, pH 9.6 0.1% SDS) to PVDF 0.45 um membrane and then blocked in 3% non-fat dry milk in 1 x PBS containing 0.1% Tween 20 for 1 h. They were then incubated in the primary antibody (collagen I and collagen III) at dilution 1:1000 overnight (4 °C) The membrane was then washed (3 × 10 min) in PBS-T (PBS 0.1% Tween 20) and incubated with HRP-conjugated anti-rabbit secondary antibody for 1 h. The membrane was washed (3 × 10 min) and incubated (1 min) in WESTAR ANTARES substrate and imaged in a G-box imaging system.

#### Western blot of high molecular weight proteins (>100 kDa)

The proteins were transferred (60 min; 8 mA/1 cm^2^) with the specific transfer buffers (anode buffer: 60 mM Tris, 40 mM CAPS, pH 9.6 15% MeOH; cathode Buffer: 60 mM Tris, 40 mM CAPS, pH 9.6 0.1% SDS) to PVDF 0.45 um membrane and then blocked in 3% non-fat dry milk in 1 x PBS containing 0.1% Tween 20 for 1 h. They were then incubated in the primary antibody (collagen I and collagen III) at dilution 1:1000 overnight (4 °C) The membrane was then washed (3 × 10 min) in PBS-T (PBS 0.1% Tween 20) and incubated with HRP-conjugated anti-rabbit secondary antibody for 1 h. The membrane was washed (3 × 10 min) and incubated (1 min) in WESTAR ANTARES substrate and imaged in a G-box imaging system.

#### Collagen I polyclonal antibody

A synthetic peptide corresponding to a sequence at the C-terminus of mouse collagen I, identical to the related rat sequence and different from the related human sequence by two amino acids (Thermofisher #PA5-95137).

#### Collagen III polyclonal antibody

Recombinant protein fragment corresponding to a region within amino acids 1180 and 1444 of human COL3A1 (Thermofisher #PA5-27828).

### Search for collagen (I) alpha-1,2 and collagen (III) alpha-1 in Porifera genomes

Genomes from six demosponge species (*Amphimedon queenslandica*, *Aplysina aerophoba*, *Chondrosia reniformis*, *Ephydatia muelleri*, *Halichondria panicea*, *Petrosia ficiformis*) and one Homoscleromorpha species (*Oscarella lobularis*) were searched using the BLAST-2.15.0+ toolkit (https://blast.ncbi.nlm.nih.gov/doc/blast-help/downloadblastdata.html^46^) to identify sequences similar to the proteins identified in spongin by LC-MS/MS or nucleotide sequences encoding them (see Fig. 4, Table 1 in the main text).

Sequences found by this method were checked with BLAST BlastX and tBLASTx to find the most similar sequences recorded in the NCBI database^47^. If a sequence was found with an evalue rating below e-25 in both the sequenced genome and BlastX or tBLASTx against the collagen sequence of *Sus Scrofa*, collagen was considered to be successfully identified. The general search principle is demonstrated in Supplementary Fig. 10.

### Identification of halogenated di- and tri-tyrosines within spongin fibers

#### Reagents

All solvents and reagents for the analyses were purchased from IRIS Biotech GmbH (Marktredwitz, Germany) or Sigma-Aldrich (St. Louis, USA). All reagents were used without purification.

#### Sample preparation

Samples of *H. communis* spongin were hydrolyzed in 6 M HCl for 24 h at 37 °C. Hydrolyzed samples were freeze-dried overnight to remove excess HCl. The solid remainder was dissolved in dd H_2_O for chromatography and mass spectroscopy analyses.

#### Analytical high-performance liquid chromatography (HPLC)

Analytical HPLC was performed on a Phenomenex Luna 5u C-18 column (5 μM particle size, 250 × 3 mm; Phenomenex, Torrance, USA) for 40 min with a flow rate of 0.5 mL/min. A linear gradient of water/acetonitrile containing 0.1% (v/v) trifluoroacetic acid was used as the mobile phase. For HPLC analysis, the monitoring wavelengths were set at a wavelength range of 210–278 nm. A two-pump system (Agilent Technologies 1100 Series) equipped with a UV/Vis detector/spectrophotometer with a 1 cm path length cell was used.

#### Electrospray ionization mass spectrometry (ESI-MS)

ESI-MS measurements were performed on an Agilent Technologies 6230 TOF LC/MS spectrometer (Applied Biosystems, USA). Nitrogen was used as a nebulizing and desolvation gas.

### Computational modeling

We obtained triple helices from the crystal structures of collagen mimetic peptides (CMPs) for type I (COL-I) and type III (COL-III) collagens, available under PDB codes 7CWK^48^ and 8HHI^49^, respectively. Subsequently, we capped the N- and C-termini with acetyl and amide groups and replaced PHE residues with TYR where the crosslinks were to be introduced. The crosslinks were strategically inserted at TYR10 of COL-III and TYR16 of COL-I, resulting in three types of crosslinks: 1Br, 2Br, and 3Br.

We solvated the structure using the TIP3P water model in a rectangular box with an edge length 0.3 nm larger than the largest axis of the model. After solvation, the systems underwent energy minimization using the steepest descent algorithm. Temperature equilibration at 300 K was achieved over the first 5 ns, followed by an equilibration MD run at 300 K using a 2 fs time step, lasting 150 ns for the system without crosslink. We then mutated PHE into TYR and their respective modifications to build the crosslinked models. Finally, the production MD run was performed with the same parameters for 51 ns. These extended simulations did not result in qualitative changes in most analyses, such as the radius of gyration (Rg) and RMSD (see Supplementary Fig. 21 and Supplementary Table 4) All MD simulations were executed using the NAMD v3b software package with the CHARMM36m forcefield^50–55^.

### Reporting summary

Further information on research design is available in the Nature Portfolio Reporting Summary linked to this article.

## Supplementary information

Supplementary Information

Description of Additional Supplementary Files

Supplementary Data 1

Reporting Summary

Transparent Peer Review file

## Source data

Source Data

## Data Availability

The molecular dynamics data generated in this study have been deposited in the github.com database [https://github.com/albertomds/collagen]. The proteomics LC-MS data generated in this study have been deposited in the ProteomeXchange partner repository MassIVE under accession number MSV000097124 [10.25345/C58P5VN6J], ProteomeXchange under accession number PXD060839, and to the Zenodo database [10.5281/zenodo.14677365]. The ESI-MS, HPLC, UV-VIS data are available in the Zenodo database [10.5281/zenodo.14741873]. The Solid State ^13^C NMR data, FTIR and Raman spectroscopy of spongin data generated for this study, as well as the unprocessed scans of SDS-PAAG and Western blots, are provided in the Source Data file. Detailed protein report (Mascot search results and LC-MS/MS analysis) is available in the Supplementary Data file. The genomes used for BLAST search in this study are available in the NCBI database under the following accession codes: GCA_000090795.2 [https://www.ncbi.nlm.nih.gov/datasets/genome/GCF_000090795.2/] (*Amphimedon queenslandica*), GCA_949841015.1 [https://www.ncbi.nlm.nih.gov/datasets/genome/GCA_949841015.1/] (*Aplysina aerophoba)*, GCA_947172415.1 [https://www.ncbi.nlm.nih.gov/datasets/genome/GCA_947172415.1/] (*Chondrosia reniformis*), GCA_013339895.1 [https://www.ncbi.nlm.nih.gov/datasets/genome/GCA_013339895.1/] (*Ephydatia muelleri*), GCA_020423275.1 [https://www.ncbi.nlm.nih.gov/datasets/genome/GCA_020423275.1/] (*Halichondria panicea*), GCA_947507565.1 [https://www.ncbi.nlm.nih.gov/datasets/genome/GCA_947507565.1/] (*Oscarella lobularis)*, GCA_947044365.1 [https://www.ncbi.nlm.nih.gov/datasets/genome/GCA_947044365.1/] (*Petrosia ficiformis*). The reference protein sequences used in this study for the Mascot search and BLAST analysis are available in the NCBI database under accession codes: XP_020922812.1 [https://www.ncbi.nlm.nih.gov/protein/XP_020922812.1] (Collagen I alpha-1 chain isoform X1 [*Sus scrofa*]), BAX02569.1 [https://www.ncbi.nlm.nih.gov/protein/BAX02569.1] (Alpha 2 chain of type I collagen [*Sus scrofa domesticus*]), XP_020922812.1 [https://www.ncbi.nlm.nih.gov/protein/XP_020922812.1] (collagen alpha-1 (I) chain isoform X1 [*Sus scrofa*]), NP_001230226.1 [https://www.ncbi.nlm.nih.gov/protein/NP_001230226.1] (Collagen alpha-1 (III) chain precursor *[Sus scrofa*]), NP_001230584.1 [https://www.ncbi.nlm.nih.gov/protein/NP_001230584.1] (collagen alpha-2(I) chain precursor [*Sus scrofa*]), XP_020922812.1 [https://www.ncbi.nlm.nih.gov/protein/XP_020922812.1] (Collagen alpha-1(I) chain isoform X1 [*Sus scrofa*]), CAQ63561.1 [https://www.ncbi.nlm.nih.gov/protein/CAQ63561.1] (fibrillar collagen COL5alpha, partial [*Amphimedon queenslandica*]), CAQ63562.1 [https://www.ncbi.nlm.nih.gov/protein/CAQ63562.1] (fibrillar collagen COL6alpha, partial [*Amphimedon queenslandica*]), XP_052314686.1 [https://www.ncbi.nlm.nih.gov/protein/XP_052314686.1] (Collagen alpha-1(I) chain-like [*Oncorhynchus keta*]), KAJ7374653.1 [https://www.ncbi.nlm.nih.gov/protein/KAJ7374653.1] (Kinesin-like protein kif15 [*Desmophyllum pertusum*]), XP_034534652.1 [https://www.ncbi.nlm.nih.gov/protein/XP_034534652.1] (LOW QUALITY PROTEIN: collagen alpha-1(IX) chain-like [*Notolabrus celidotus*]), P18503.1 [https://www.ncbi.nlm.nih.gov/protein/P18503.1] (short-chain collagen C4 [*Ephydatia muelleri*]), XP_019854257.1 [https://www.ncbi.nlm.nih.gov/protein/XP_019854257.1] (collagen alpha-1(I) chain [*Amphimedon queenslandica*]), XP_020906601.1 [https://www.ncbi.nlm.nih.gov/protein/XP_020906601.1] (Collagen alpha-1(I) chain [*Exaiptasia diaphana*]), CAI8027724.1 [https://www.ncbi.nlm.nih.gov/protein/CAI8027724.1] (Collagen alpha-1(XXIV) chain [*Geodia barretti*]). Unless otherwise stated, all data supporting the results of this study can be found in the article, supplementary, and source data files. Source data are provided with this paper.
