# Supplementary material for: Discovery of mammalian collagens I and III within ancient poriferan biopolymer spongin
Source: Nat Commun. 2025 Mar 13;16:2515. doi: 10.1038/s41467-025-57460-y (PMC11906918; doi:10.1038/s41467-025-57460-y)
Supplement: Supplementary file 1 — Supplementary Information [file 41467_2025_57460_MOESM1_ESM.pdf]

Discovery of mammalian collagens I and III within ancient poriferan biopolymer spongin

Hermann Ehrlich[1,2*], Ivan Miksik[3], Mikhail V. Tsurkan[4], Paul Simon[5], Filip Porzucek[1,6], Jakub Dalibor Rybka[1,6], Monika Mankowska[1,6], Roberta Galli[7], Christine Viehweger[8], Erica Brendler[9], Alona Voronkina[10,11], Martyna Pajewska-Szmyt[1], Aleksei Tabachnik[12], Konstantin R. Tabachnick[13], Carla Vogt[9], Marcin Wysokowski[2], Teofil Jesionowski[2], Tomasz Buchwald[14], Miroslaw Szybowicz[14], Kinga Skieresz-Szewczyk[15], Hanna Jackowiak[15], Alexander Ereskovsky[16], Amadeus C. S. de Alcântara[17,18,19], Alberto M. dos Santos[19,20], Clauber H. S. da Costa[19,20], Sofia E. Arevalo[17,21], Munir S. Skaf[19,20], Markus J. Buehler[17,21,22*]

1. Center for Advanced Technology, Adam Mickiewicz University, 61614 Poznan, Poland
2. Institute of Chemical Technology and Engineering, Faculty of Chemical Technology, Poznan University of Technology, 60965 Poznan, Poland
3. Department of Analytical Chemistry, Faculty of Chemical Technology, University of Pardubice, 53210 Pardubice, Czech Republic
4. Leibniz Institute of Polymer Research Dresden, 01069 Dresden, Germany
5. Max Planck Institute for Chemical Physics of Solids, 01187 Dresden, Germany
6. NanoBioMedical Centre, Adam Mickiewicz University in Poznań, 61-614 Poznań, Poland
7. Department of Medical Physics and Biomedical Engineering, Faculty of Medicine Carl Gustav Carus, TechnischeUniversität Dresden, 01307 Dresden, Germany
8. Institute of Geology, Chair of Hydrogeology and Hydrochemistry, TU Bergakademie Freiberg, 09599 Freiberg, Germany
9. Institute of Analytical Chemistry, TU Bergakademie Freiberg, 09599 Freiberg, Germany
10. Institute for Nanoscale and Biobased Materials, TU Bergakademie Freiberg, 09599 Freiberg, Germany
11. Department of Pharmacy, National Pirogov Memorial Medical University, Vinnytsya, 21018 Vinnytsia, Ukraine
12. Department of Marine Biology, Leon H. Charney School of Marine Sciences, University of Haifa, Haifa 31905, Israel
13. International Institute of Biomineralogy GmbH, 09599 Freiberg, Germany;
14. Institute of Materials Research and Quantum Engineering, Faculty of Materials Engineering and Technical Physics, Poznan University of Technology, 60965 Poznan, Poland
15. Department of histology and embryology, Poznań University of Life Sciences, 60625 Poznan, Poland
16. Aix Marseille University, Avignon Université, CNRS, IRD, IMBE, Marseille, France
17. Laboratory for Atomistic and Molecular Mechanics (LAMM), Massachusetts Institute of Technology, Cambridge, Massachusetts, USA
18. Department of Computational Mechanics, School of Mechanical Engineering, Universidade Estadual de Campinas (UNICAMP), Campinas, Sao paulo, Brazil
19. Center for Computing in Engineering & Sciences (CCES), Universidade Estadual de Campinas (UNICAMP), Campinas, Sao paulo, Brazil
20. Institute of Chemistry, Universidade Estadual de Campinas (UNICAMP), Campinas, Sao Paulo, Brazil
21. Department of Civil and Environmental Engineering, Massachusetts Institute of Technology, Cambridge, Massachusetts, USA
22. Center for Computational Science and Engineering, Schwarzman College of Computing, Massachusetts Institute of Technology, Cambridge, Massachusetts, USA

Supplementary Figures

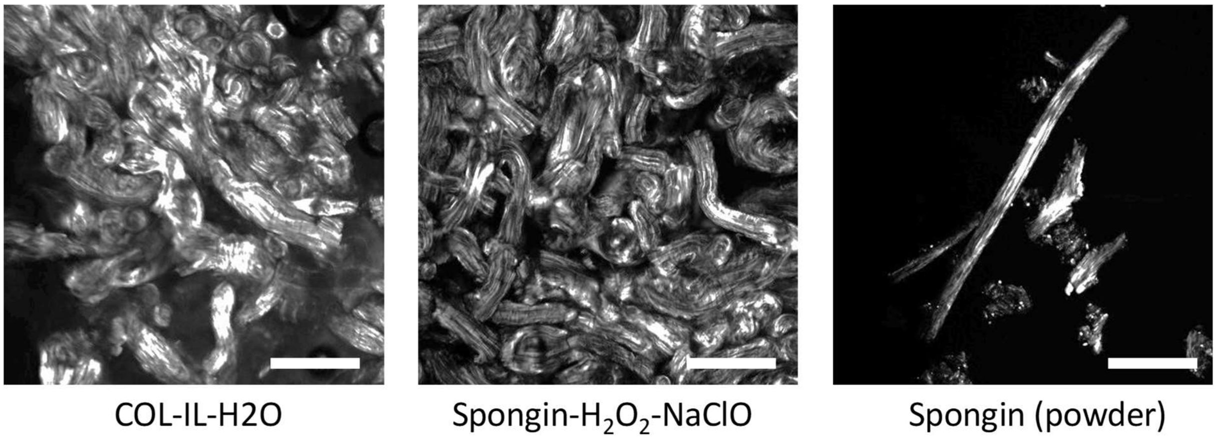

Supplementary Figure 1 | SHG images of collagen and spongin samples under study. Scale bars: 100 µm.

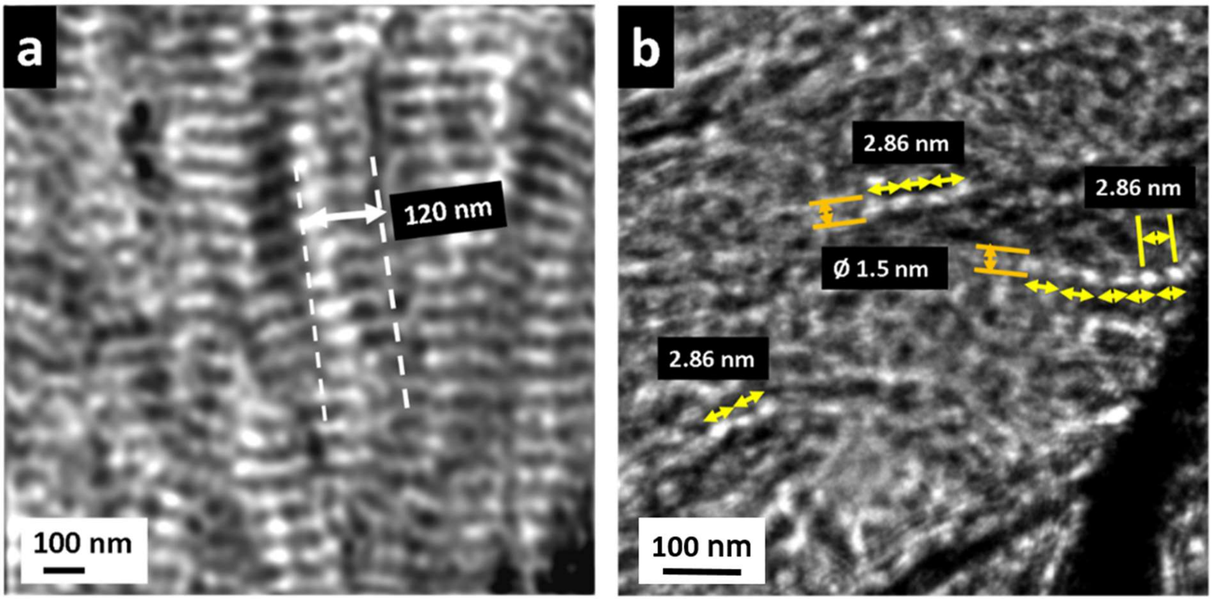

Supplementary Figure 2 | TEM micrographs of ultramicrotomy of non-stained collagen I fiber structure in bone. (a) Striation of 67 nm within 120 diameter collagen I fibril of rabbit femur (for details see[1]). (b) A periodicity of 2.86 nm was detected along triple helices with 1.5 nm diameter in the human femur (for details see[2]). The measured spacings were confirmed by repeating the measurements at least at 3 different regions of the sample.

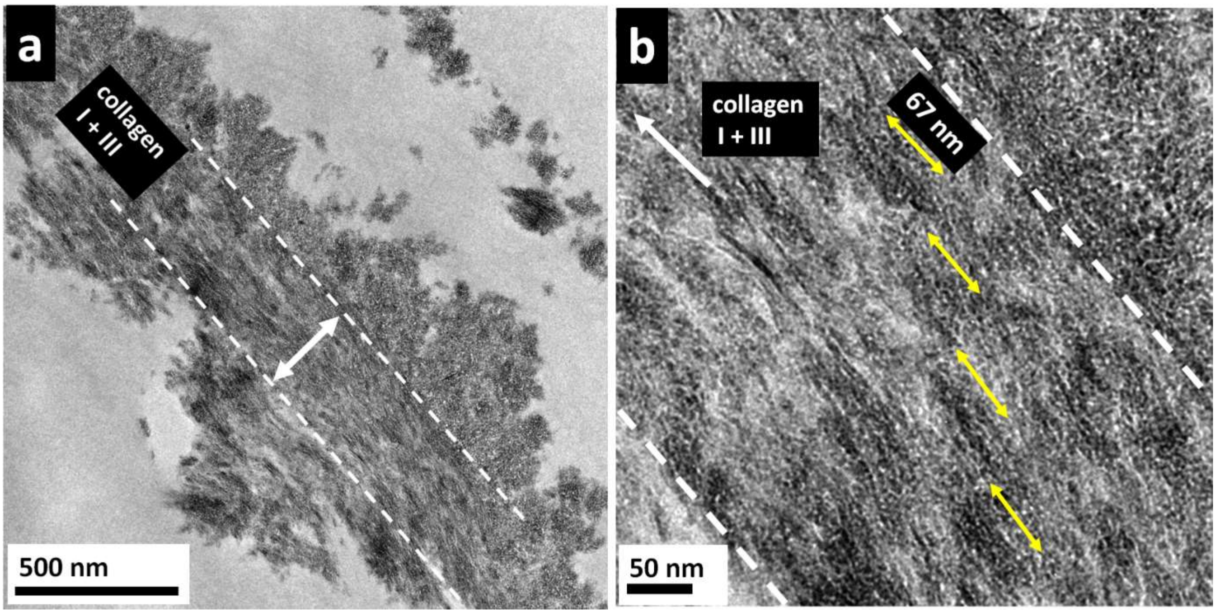

Supplementary Figure 3 | TEM micrographs of ultramicrotomy of uranyl stained spongin fiber. (a) Overview of spongin nano-fibre-containing collagen I and III (b) At higher magnification, the indication of the 67 nm striation of collagen I is observed; see arrows. The nanostructural parameters of these nanofibrils correspond nearly excellently to that observed using the same method for collagen I in rat tibia (for details see[3]). The measured spacings were confirmed by repeating the measurements at least at 3 different regions of the sample.

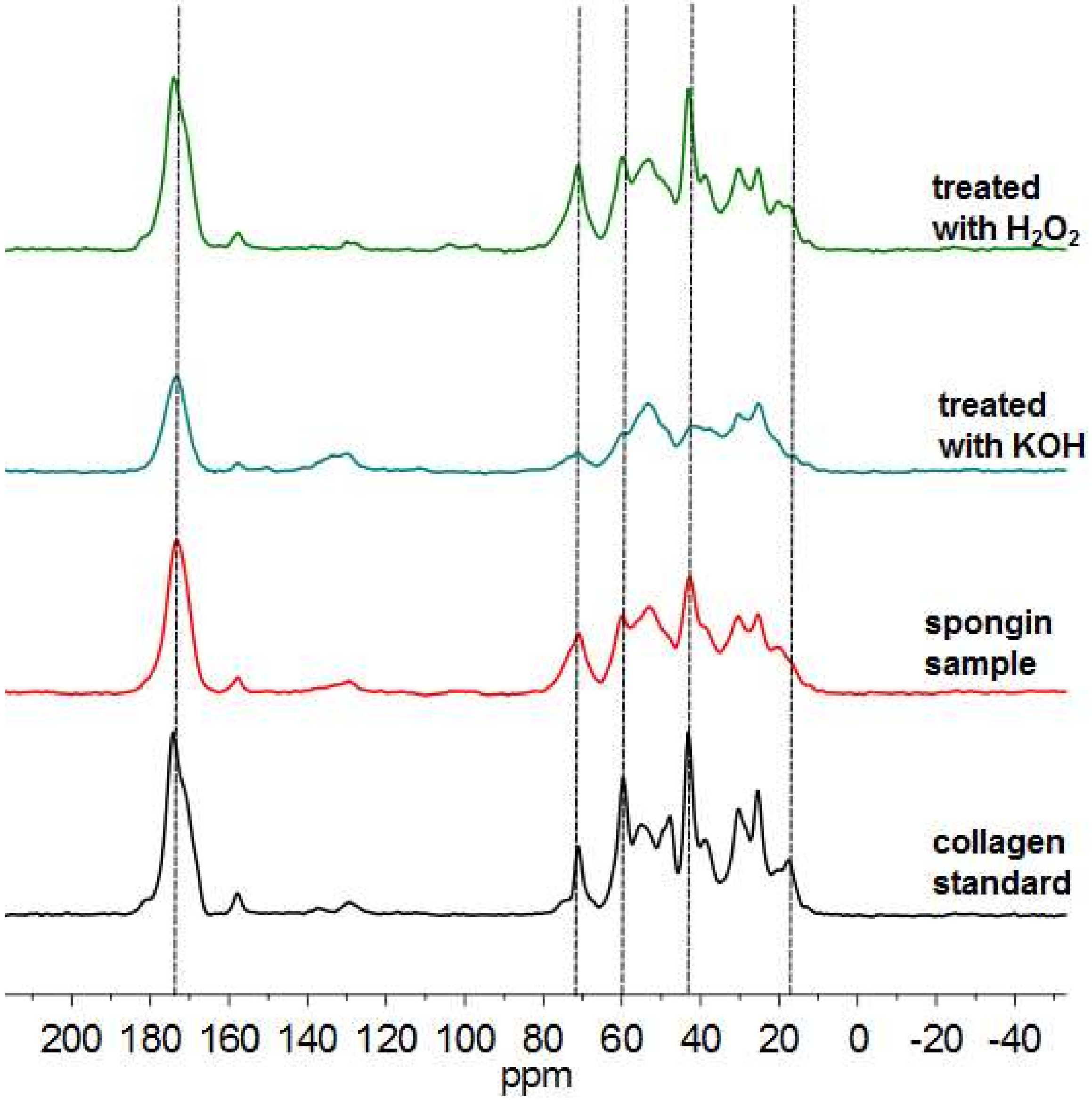

Supplementary Figure 4 | Comparison of the ^{13}C-CP-MAS NMR spectra of collagen standard (black line), the spongin sample (red line), the spongin sample after treatment with KOH (blue line), and after the treatment with H_2O_2 (green line). Source data are provided as a Source Data file.

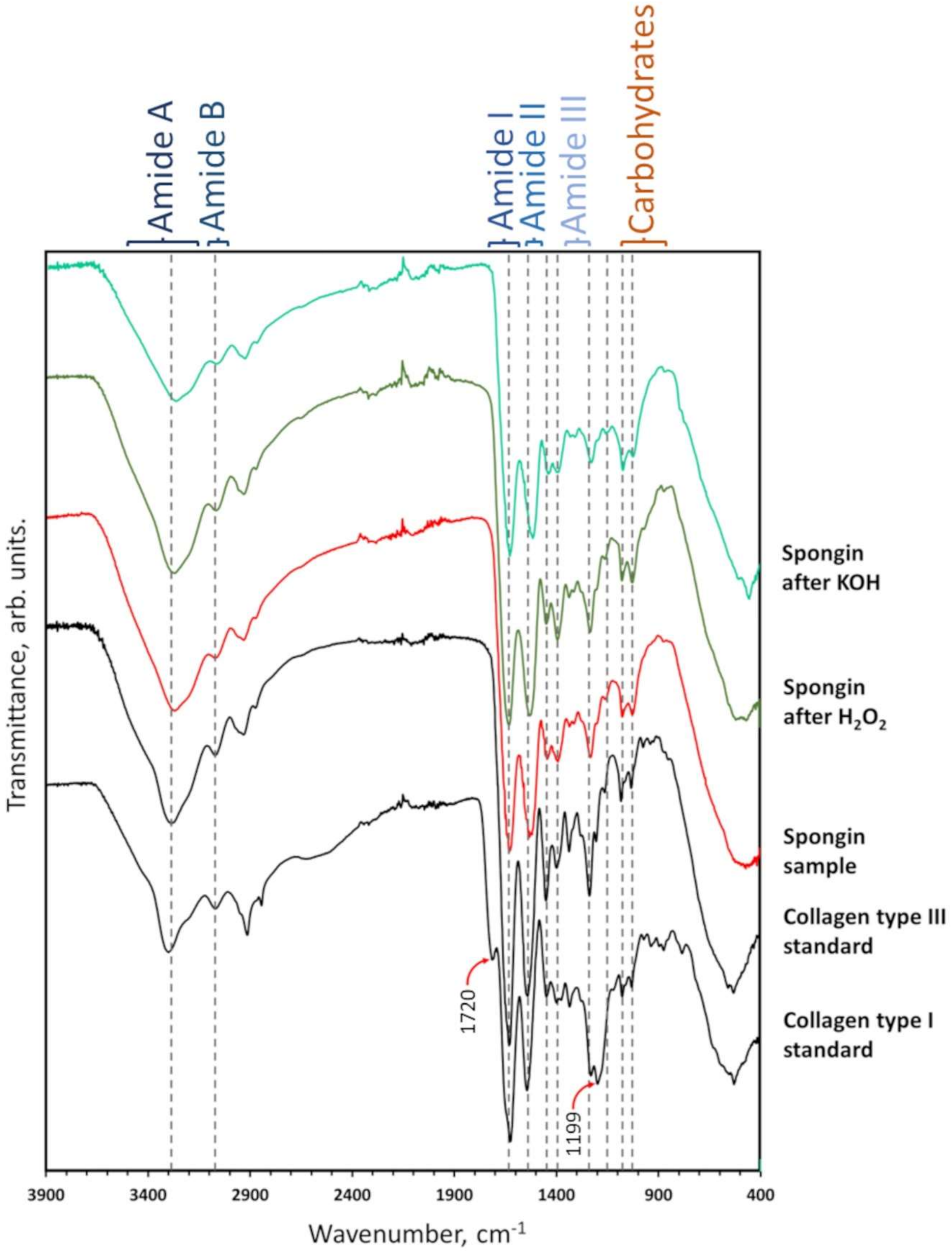

Supplementary Figure 5 | ATR-FTIR spectra registered for collagen standards (black lines), the spongin sample (red line), the spongin sample treated with KOH (cyan line), and the spongin sample treated with H_2O_2 (green line). Source data are provided as a Source Data file.

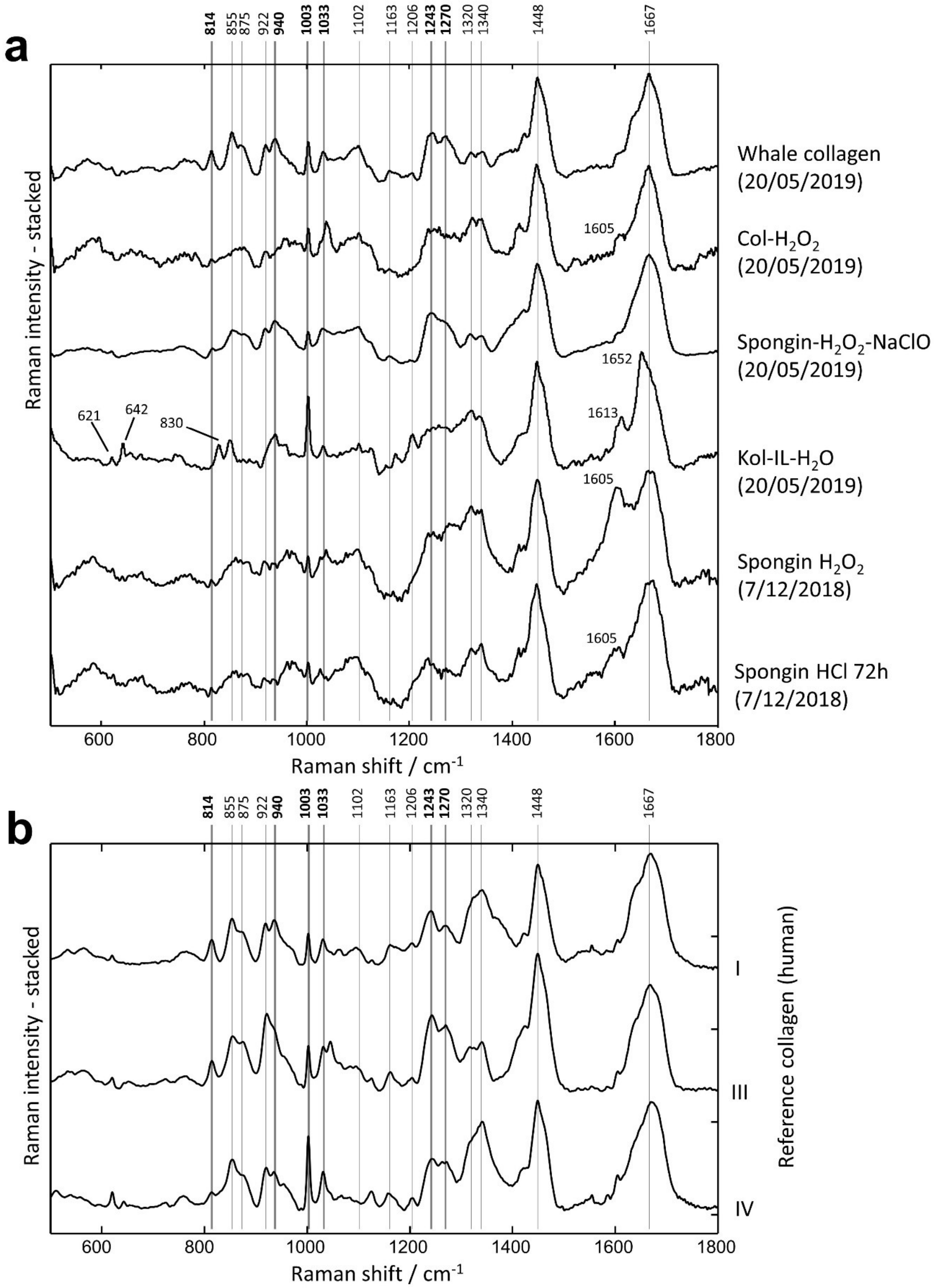

Supplementary Figure 6 | Raman spectroscopy. **a**: Spectra of spongin and collagen samples. **b**: Reference spectra of human collagen type I, III, and IV. Band positions are indicated, and bands useful for collagen type identification, as described in the text, are highlighted in bold. Source data are provided as a Source Data file.

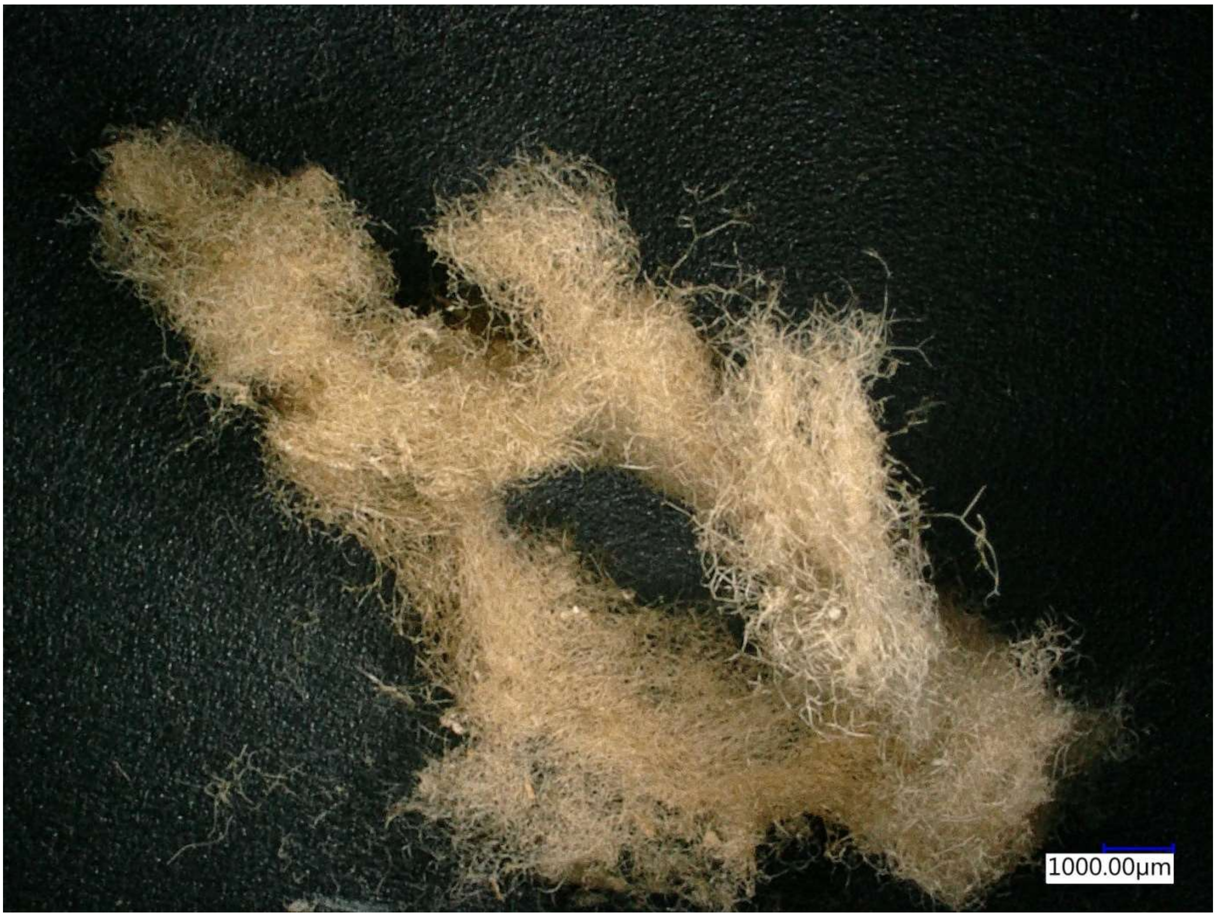

Supplementary Figure 7 | Digital microscopy image of the microfragment of *H. communis* spongin used in the study.

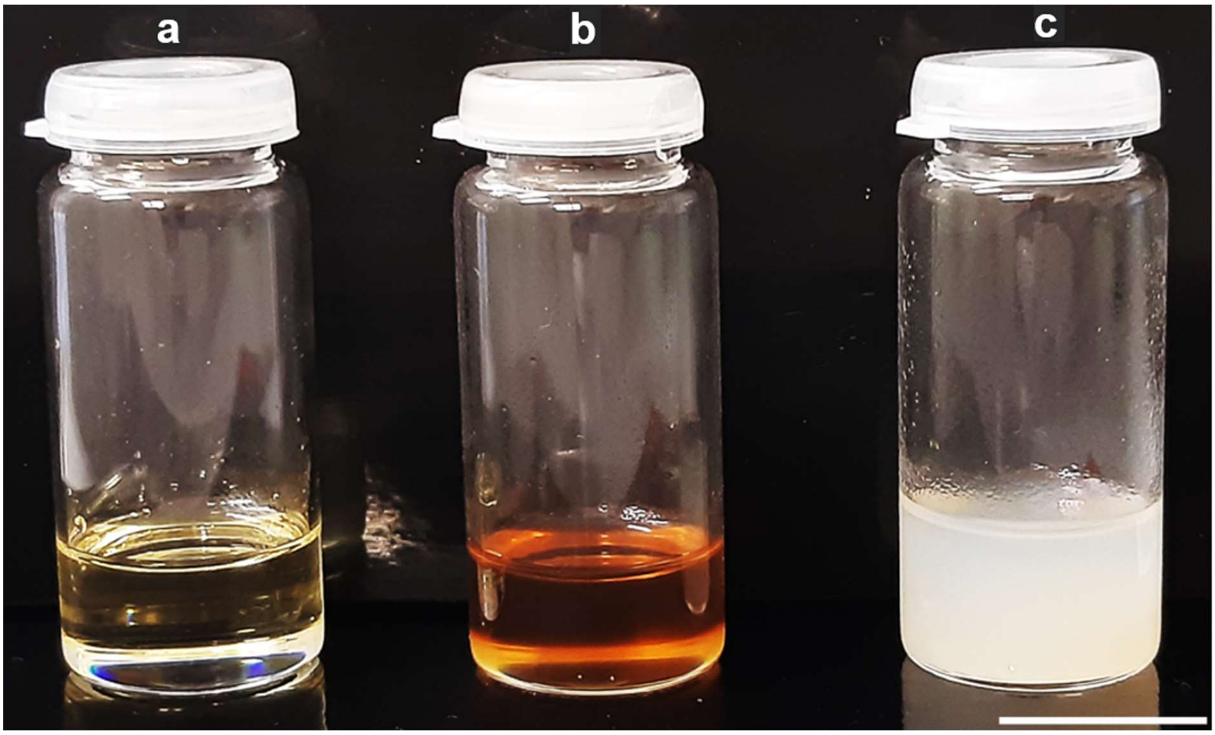

Supplementary Figure 8 | Spongin extracts. a: 1-butyl-methylimidazolium acetate; **b**: spongin dissolved in 1-butyl-methylimidazolium acetate; and **c**: precipitate formed after mixing of solution B with propan-2-ol where Collagen type III has been identified using proteomics. The scale is 1 cm.

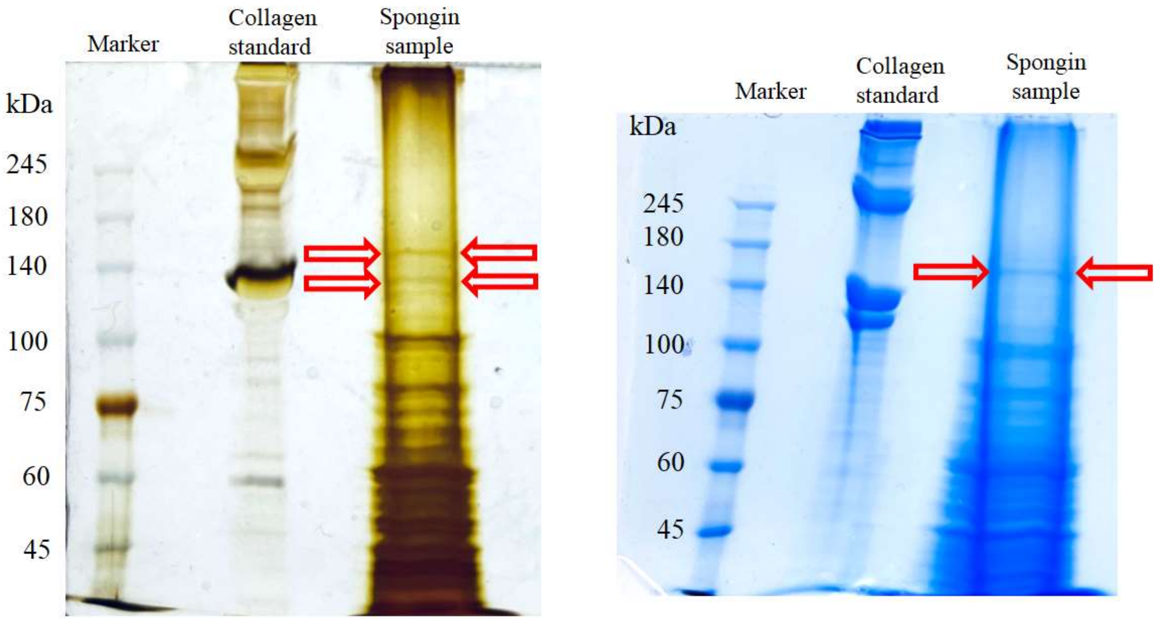

Supplementary Figure 9 | SDS-PAGe imagery of proteins isolated from spongin using NaCL extraction. Both Silverstain – (left image) and Coomassie blue – (right image) based techniques show existence of some still not identified proteins/peptides additionally to typical collagen subunits bands (arrows). Source data are provided as a Source Data file.

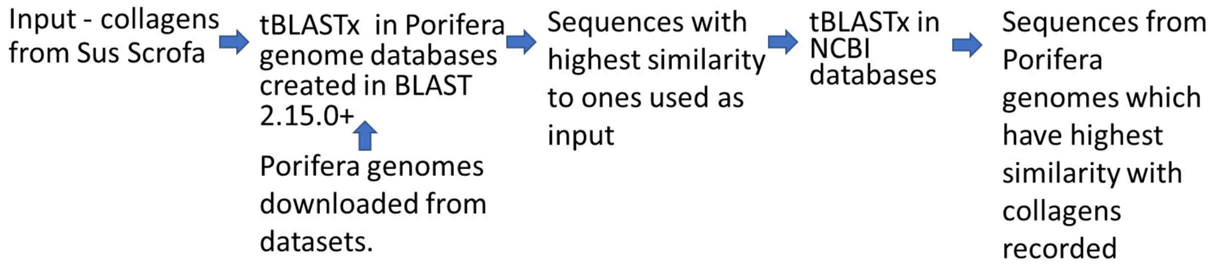

Supplementary Figure 10 | Search principle used for the search for Collagen (I) alpha-1,2 and Collagen (III) alpha-1 in Porifera genomes

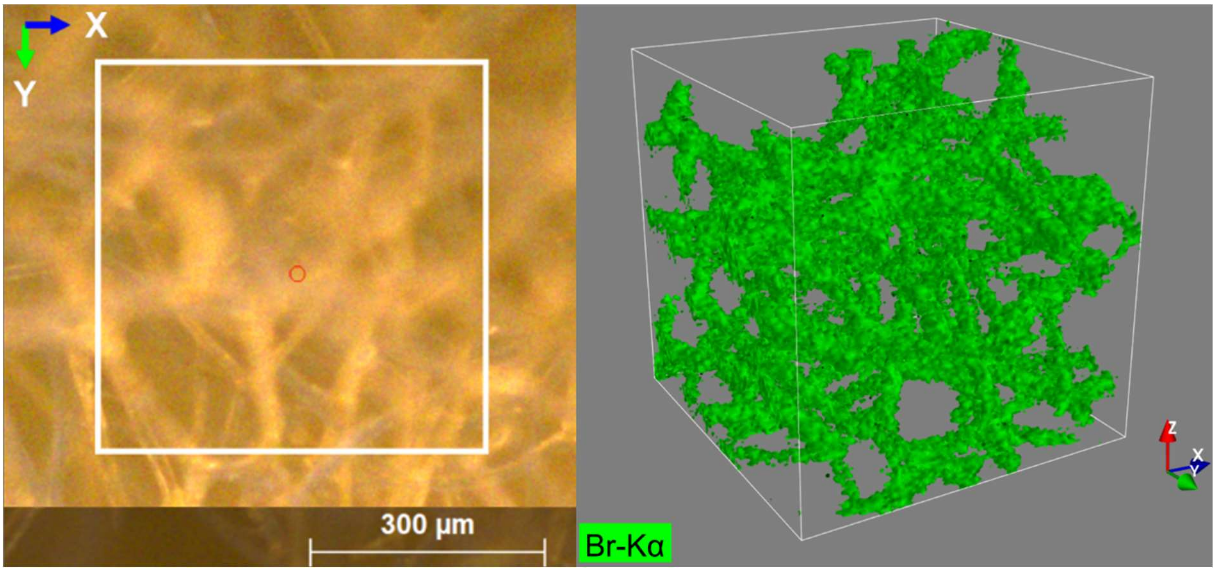

Supplementary Figure 11 | CMXRF analytics. 3D microstructure of spongin vs the model of Br(Kα) distribution in the *H. communis* spongin sample under study.

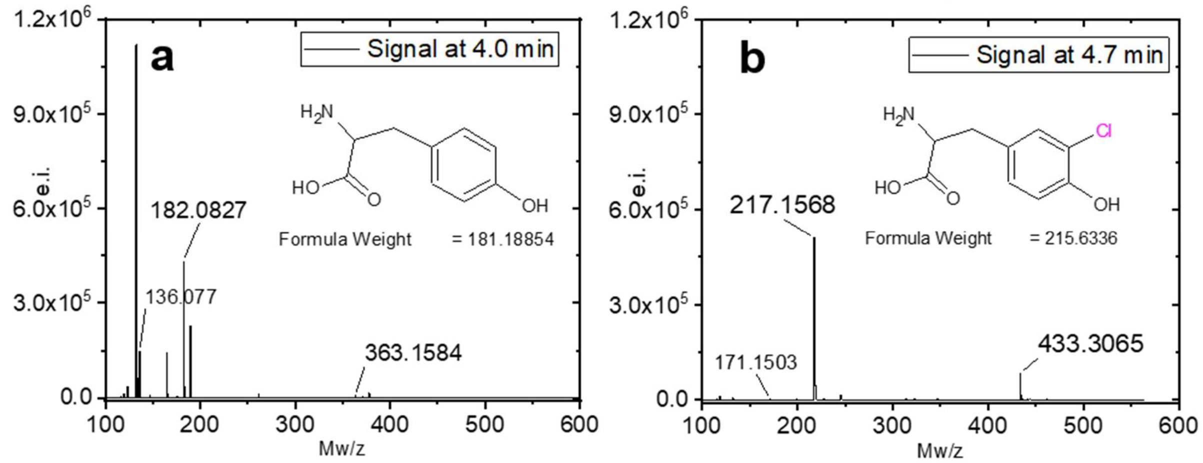

Supplementary Figure 12 | ESI-MS analysis of tyrosine and 3-Cloro-tyrosine compounds that elute in HPLC before 5 min (see Figure 5 in the main text). A: ESI-MS spectrum at 4.0 min. Although the signals are poorly resolved and eluted as a mixture of several compounds, a Tyrosine MS signal can be seen at 4.00 min elution time, indicating its native presence. An even molecular weight signal $[M+H]^+$ in the MS (m/z=182.08) indicates that the molecule contains one amine. A characteristic for amino acids signal of the moiety $[M+H-H2O-CO]^+$ signal at m/z= 136.0 and dimeric moiety $[2M+H]^+$ signal (mz= =363.159) were expected for amino acid ESI-MS at the low pH of the HPLC analysis. B: ESI-MS spectrum of the compound at 4.7 min. The mass spectrum signal of 3-chloro-Tyrosine can be observed in HPLC analysis at 4.7 min eluting in a mixture of other amino acid moieties. Halogenated tyrosines are more hydrophobic and eluted at a longer time in RP-HPLC. Similarly to the Tyrosine molecule, the MS spectra of 3-chlor-tyrosine contain molecular ion signal $[M+H]^+$ (m/z=217.15), and the corresponding signals of decarboxylated $[M+H- H_2CO_2]^+$ (m/z=171.15) and dimeric $[2M+H]^+$ (m/z=433.20) moieties. The presence of both tyrosine and 3-cloro-tyrosine amino acid residues in spongin was known. Source data are provided as a Source Data file.

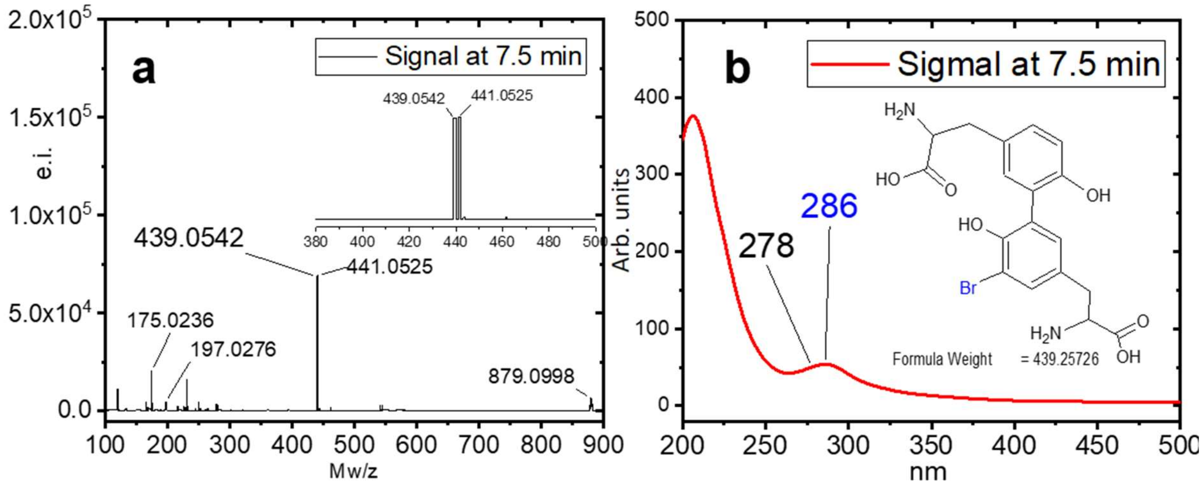

Supplementary Figure 13 | ESI-MS and UV-Vis analysis of compound that elutes et 7.5 minutes in the HPLC analysis (see Figure 5 in the main text). **a**: ESI-MS spectrum. The compound was identified by the characteristic duplet splitting of the molecular ion signal $[M+H]^+$ (m/z= 439.05 and 441.05), revealing the presence of one Br atom in the molecule. The molecule has $[2M+2H-H_2CO_2]^{+2}$ (m/z= 197.02) and $[2M+2H-2H_2CO_2]^{+2}$ (m/z= 175.02), which indicates the molecule contains two amino acid groups. Similarly to the tyrosine spectra, the MS spectrum of this molecule has a characteristic signal of dimeric moiety $[2M+H]^+$ signal (m/z=879.1) is clearly seen in the MS with characteristic triplet splitting, which is characteristic of the two Br atoms in the dimer noncovalent dimer of the molecular ion. **b**: The UV-Vis spectrum has a slight red shift band (286 nm) of the characteristic Tyrosine moiety absorbance (278 nm), which is expected for halogenated tyrosine. Together, the HPLC-MS analysis and characteristic UV-Vis spectrum allowed us to confirm the chemical structure of the 5-bromo-3,3'-dityrosine, which had previously never been reported in sponges. Source data are provided as a Source Data file.

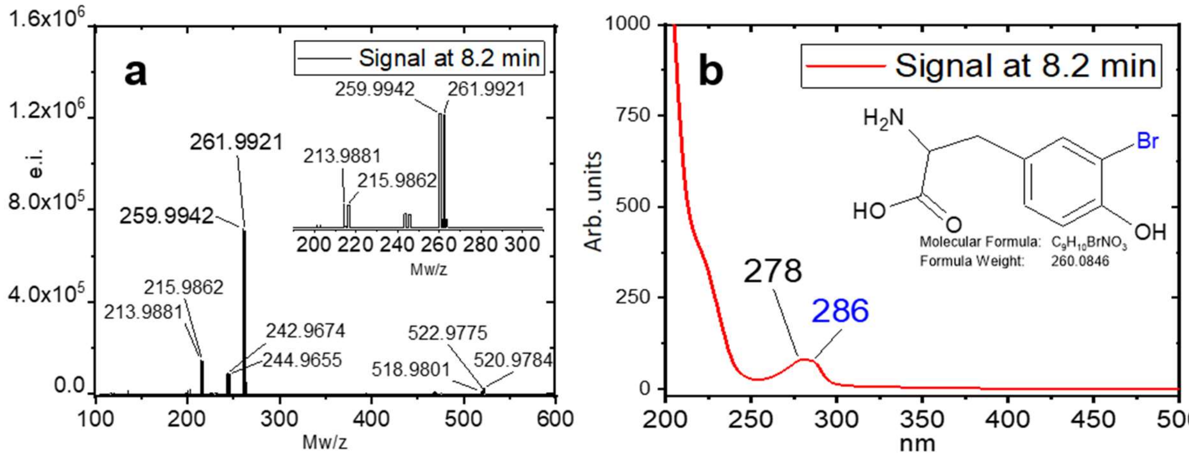

Supplementary Figure 14 | ESI-MS and UV-Vis analysis of compound that elutes at 8.2 minutes in the HPLC analysis (see Figure 5 in the main text). **a**: ESI-MS spectrum. The compound was identified by the characteristic duplet splitting of the molecular ion signal $[M+H]^{+}$ (m/z= 259.99 and 261.99), revealing the presence of one Br atom in the molecule. The spectrum has $[M+2H-H_2CO_2]^{+2}$ signal (m/z= 213.9 and 215.9), which indicates that the molecule contains one amino acid group. Similarly to the tyrosine spectrum, the MS spectrum of this molecule has a characteristic signal of dimeric moiety $[2M+H\ ]^{+}$ signal (m/z=520.97) with a triplet splitting, which is characteristic of the two Br atoms in the dimer noncovalent dimer of the molecular ion. **b**: The UV-Vis spectrum has a slight red shift band (286 nm) of the characteristic Tyrosine moiety absorbance (278 nm), which is expected for halogenated tyrosine. Together, the HPLC-MS analysis and characteristic UV-Vis spectrum confirmed the chemical structure of the 3-Bromo-tyrosin, which was known to be present in spongin. Source data are provided as a Source Data file.

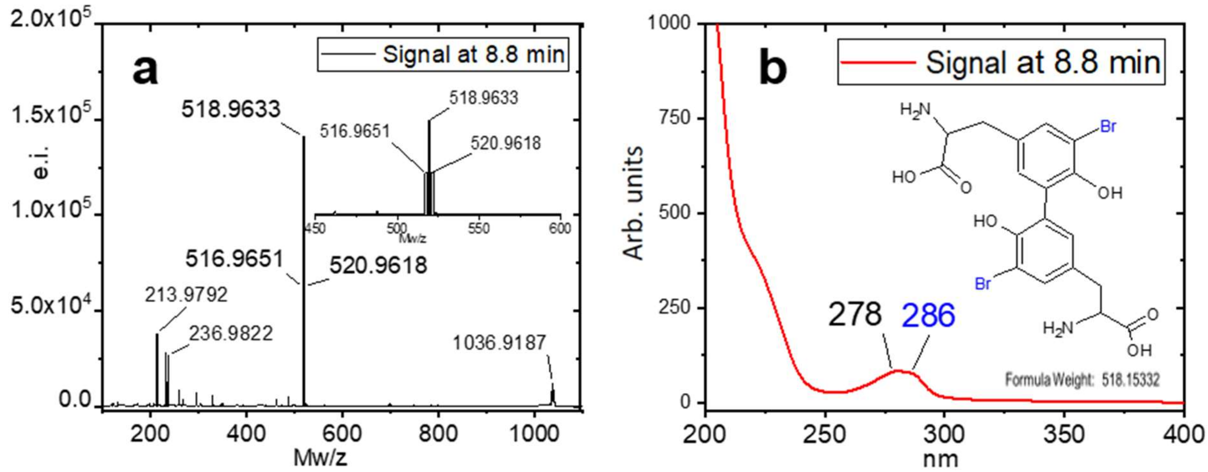

Supplementary Figure 15 | ESI-MS and UV-Vis analysis of compound that elutes at 8.8 minutes in the HPLC analysis (see Figure 5 in the main text). **a**: ESI-MS spectrum. The compound was identified by the characteristic triplet splitting of the molecular ion signal $[M+H]^+$ (m/z= 516.97 518.96 and 520.96), revealing the presence of two Br atoms in the molecule. The molecule has $[M+2H-2H_2CO_2]^{+2}$ (m/z= 213.9) and $[M+2H-H_2CO_2]^{+2,}$ which indicates the molecule contains two amino acid groups. Similarly to the tyrosine spectra, the MS spectrum of this molecule has a characteristic signal of dimeric moiety $[2M+H]^+$ signal (m/z=1036.91) is clearly seen in the MS with multi-splitting, which is indicative of more than 3 Br atoms in the dimer noncovalent dimer of the molecular ion. **b**: The UV-Vis spectrum has a slight red shift band (286 nm) of the characteristic Tyrosine moiety absorbance (278 nm), which is expected for halogenated tyrosine. The HPLC-MS analysis and characteristic UV-Vis spectrum confirmed the chemical structure of the 5,5′-dibromo-3,3'-dityrosine, which had previously never been reported in sponges. Source data are provided as a Source Data file.

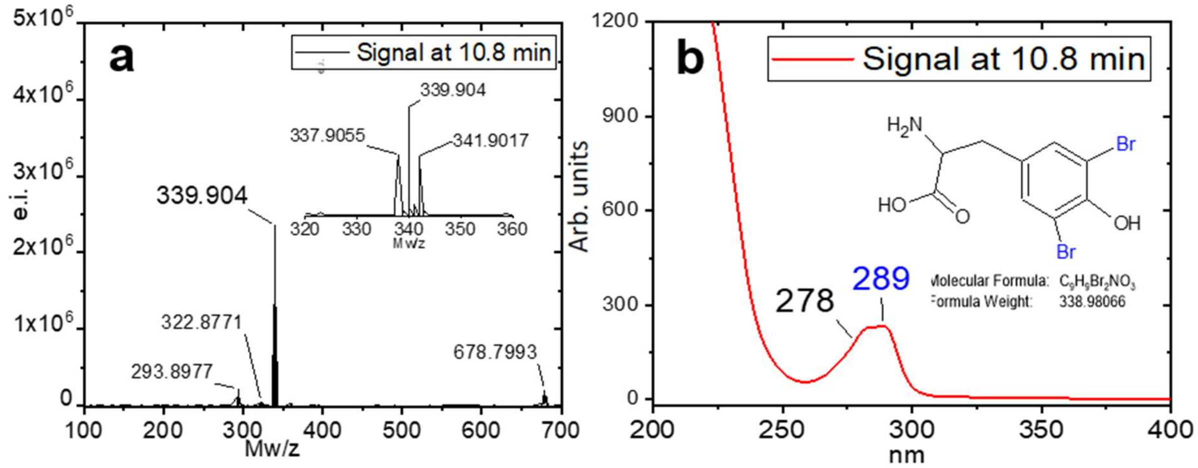

Supplementary Figure 16 | ESI-MS and UV-Vis analysis of compound that elutes at 10.8 minutes in the HPLC analysis (see Figure 5 in the main text). **a**: MS spectrum. The compound was identified by the characteristic triplet splitting of the molecular ion signal $[M+H]^+$ (m/z= 337.91; 339.90 and 341.90), revealing the presence of two Br atoms in the molecule. The molecule has $[M+2H-H_2CO_2]^{+2}$ (m/z= 293,9), which indicates the molecule contains one amino acid group. Similarly to the tyrosine spectra, the MS spectrum of this molecule has a characteristic signal of dimeric moiety $[2M+H]^+$ signal (m/z=678.8) is clearly seen in the MS with multi-splitting, which is characteristic of the multiple Br atoms in the dimer noncovalent dimer of the molecular ion. **b**: The UV-Vis spectrum has a slight red shift band (289 nm) of the characteristic Tyrosine moiety absorbance (278 nm), which is expected for halogenated tyrosine. Together, the HPLC-MS analysis and characteristic UV-Vis spectrum allowed us to confirm the chemical structure of the 3,5-diBromo-tyrosin, which was known to be present in spongin. Source data are provided as a Source Data file.

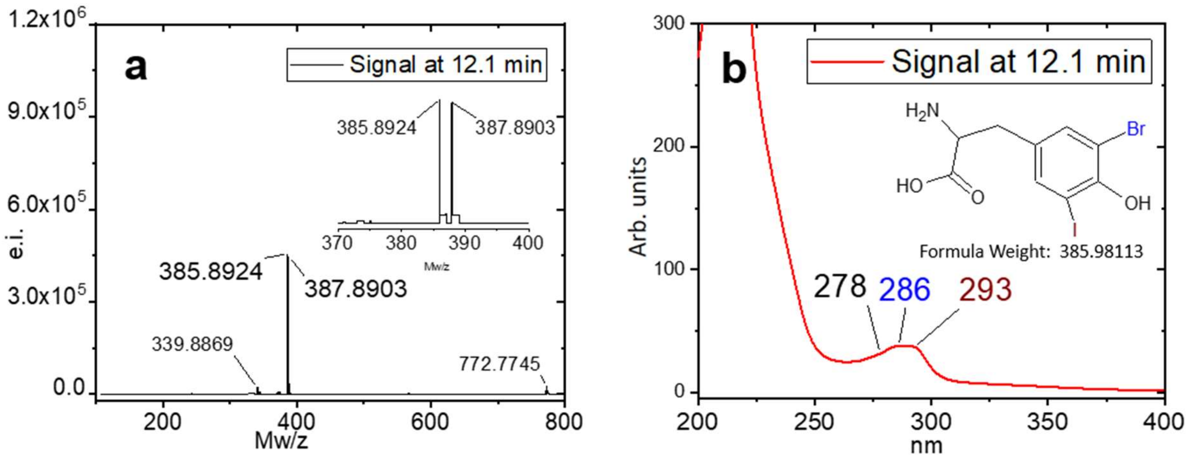

Supplementary Figure 17 | ESI-MS and UV-Vis analysis of compound that elutes at 12.1 minutes in the HPLC analysis (see Figure 5 in the main text). **a**: MS spectrum. The compound was identified by the characteristic duplet splitting of the molecular ion signal $[M+H]^+$ (m/z= 385.89 and 341.89), revealing the presence of one Br atom in the molecule. The molecule has $[M+2H-H_2CO_2]^{+2}$ (m/z= 339.9 and 215.9), which indicates the molecule contains one amino acid group. Similarly to the tyrosine spectra, the MS spectrum of this molecule has a characteristic signal of dimeric moiety $[2M+H\,]^+$ signal (m/z=772.77) is seen in the MS with characteristic triplet splitting, which is characteristic of the two Br atoms in the dimer noncovalent dimer of the molecular ion. **b**: The UV-Vis spectrum has a slight red shift band (286 nm) and another band at 293 nm of the characteristic Tyrosine moiety absorbance (278 nm). The presence of two bands in the UV spectrum indicates two different substitutions in the tyrosine's *para*-cresol ring. Lower energy (293 nm) in the second band suggests it is an iodine atom that fits perfectly to the molecule's molecular weight. Together, the HPLC-MS analysis and characteristic UV-Vis spectrum allowed us to confirm the chemical structure of the 3-bromo-5-iodotyrosine, which was known to be present in spongin. Source data are provided as a Source Data file.

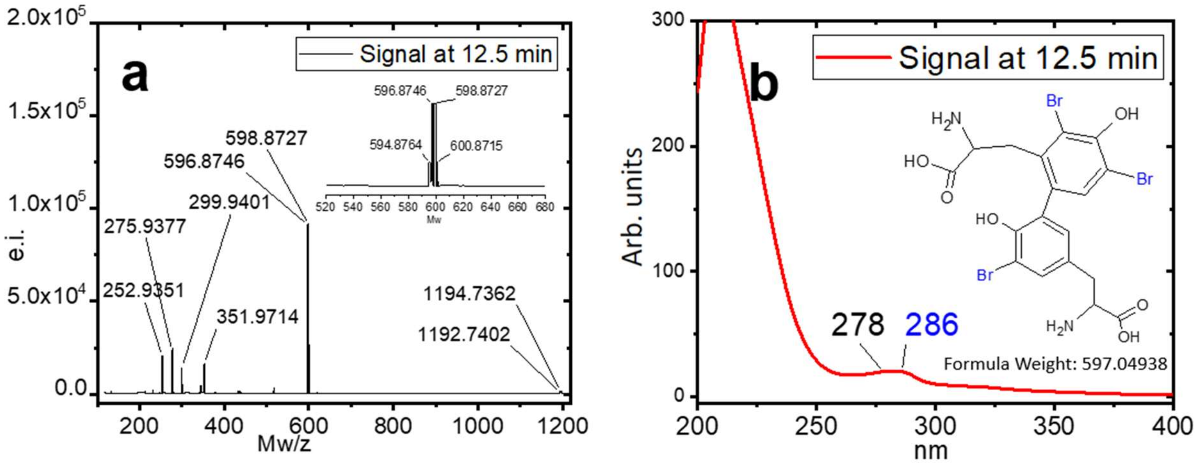

Supplementary Figure 18 | ESI-MS and UV-Vis analysis of compound that elutes at 10.8 minutes in the HPLC analysis (see Figure 5 in the main text). **a**: MS spectrum. The compound was identified by the characteristic quartet splitting of the molecular ion signal $[M+H]^+$ (m/z= 594.87; 596.87; 598.87 and 600.87), revealing the presence of three Br atoms in the molecule. The molecule has $[M+2H-2H_2CO_2]^{+2}$ (m/z= 252.93) and $[M+2H-H_2CO_2]^{+2}$ (m/z= 275.93), which indicates the molecule contains two amino acid groups. Similarly to the tyrosine spectra, the MS spectrum of this molecule has a characteristic signal of dimeric moiety $[2M+H\,]^+$ signal (m/z=1192.74) is clearly seen in the MS with multi-splitting, which is indicative of multiple atoms in the dimer noncovalent dimer of the molecular ion. **b**: The UV-Vis spectrum has a slight red shift band (286 nm) of the characteristic Tyrosine moiety absorbance (278 nm), which is expected for halogenated tyrosine. Although all *para*, *ortho,* and *meta* tyrosines can be formed by unspecific oxidation of the phenylalanine, following the formation of the Brominated tyrosines and dityrosine can go only to the *ortho*- and *para*- position to the -OH group of the formed tyrosine. Hence, it allowed defining the compound's structure by the exclusion method. Therefore, one of the tyrosines should be a meta substitute to include three atoms of brom in one dityrosine molecule per the above-described rule. The HPLC-MS analysis and characteristic UV-Vis spectrum confirmed the chemical structure of the 4,6,5′-tribromo-2,3'-dityrosine, which had previously never been reported in sponges. Source data are provided as a Source Data file.

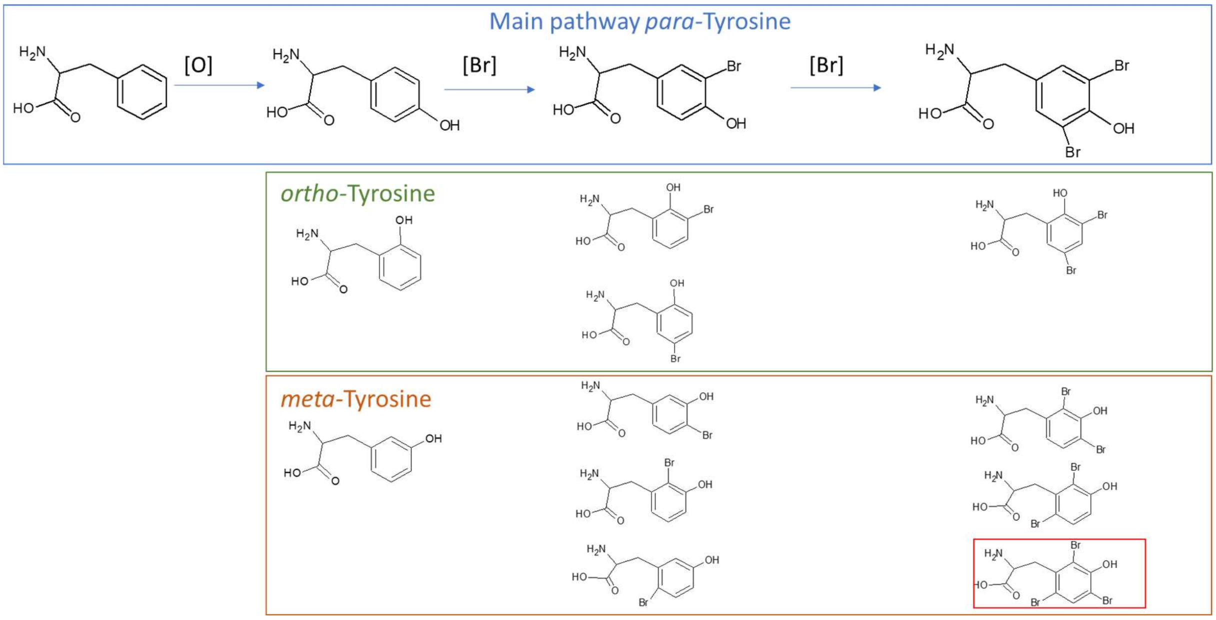

Supplementary Figure 19 | The mechanism of the formation of tyrosine and its bromine derivatives.

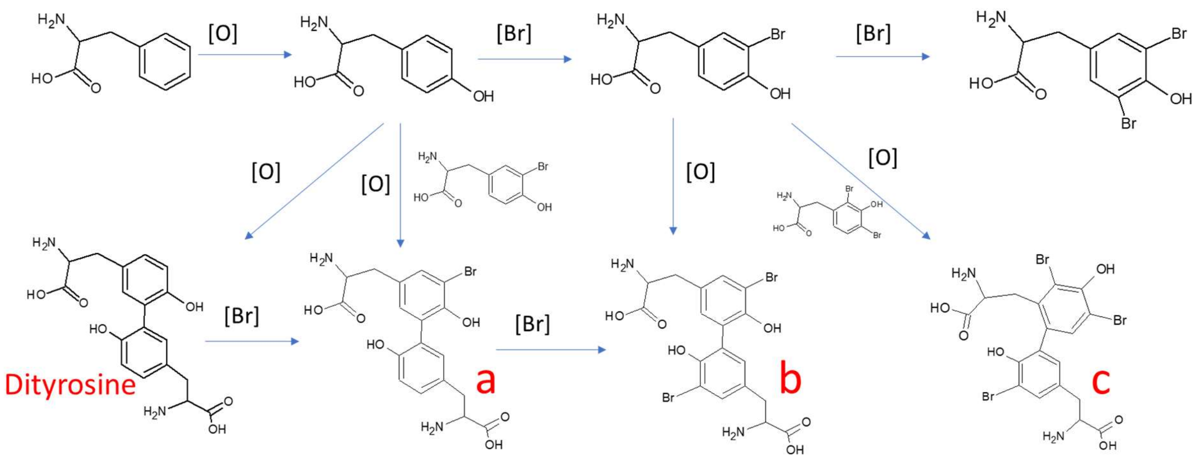

Supplementary Figure 20 | The mechanism of the formation of dityrosine and its bromine derivatives.

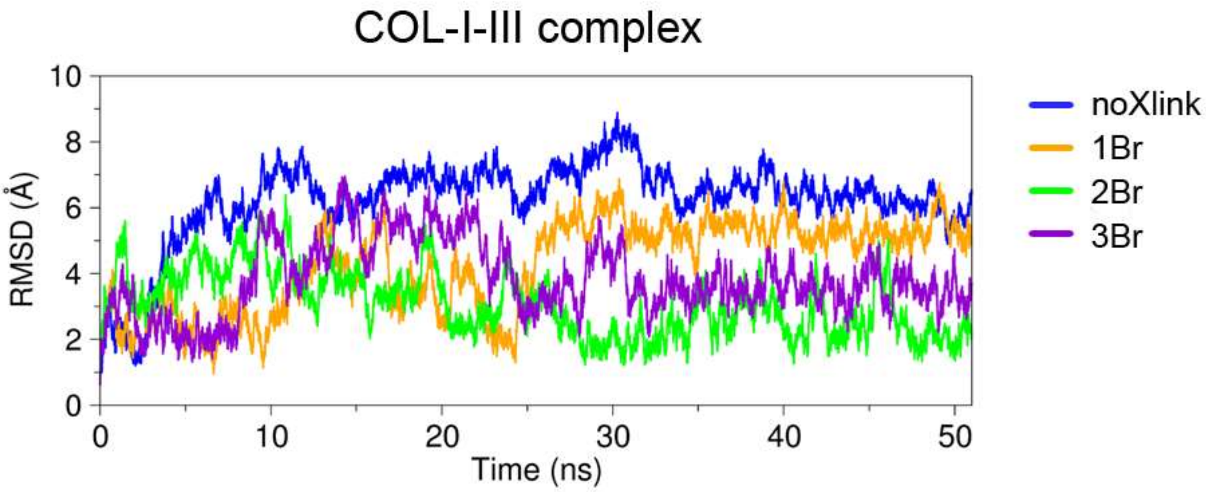

Supplementary Figure 21 | Root Mean Squared Deviation (RMSD) of combined collagen structures for all systems.

Supplementary Tables

Supplementary Table 1 | Band assignment to molecular vibration in collagen standards and spongin samples under study

Raman shift / cm^{-1}	***Band assignment to molecular vibration***	***Reference***
621	C-C deformation in phenylalanine	4
642	C-C deformation in tyrosine	4
**814**	C-O-C stretching of collagen crosslink, or backbone C-C stretching	5-7
830, 855	doublet of tyrosyl residues	5
875	C-C stretching in hydroxyproline	5
922	C-C stretching in proline ring	5, 6
**940**	C-C stretching in proline backbone	6, 7
**1003**	C-C stretching in phenylalanine ring	4-7
**1033**	C-H deformation in phenylalanine, C-N stretching in proline	5-7
1102	C-C and C-N stretching	4
1163	CH_2 deformation	7
1206	Amide III of hydroxyproline, tyrosine	4
**1243**	Amide III (C-N deformation, related to proline content in collagens)	5, 6
**1270**	Amide III (N-H deformation, related to proline content in collagens)	5, 6
1320	C-H deformation	6, 7
1340	CH_2 deformation	7
1448	CH_3 deformation	7
1605	C=C deformation in phenylalanine and tyrosine	4
1667 (1652)	Amide I	4, 5

Supplementary Table 2

Sequences found in Porifera genomes similar to Sus scrofa COL1A1, COL1A2 and COL3A1.

**Gene from Table 1**	**Sponge species**	**e-value**	**Similarity score (bits)**	**BLASTX results - identified sequences with highest similarity value**
Collagen I alpha-1 chain isoform X1 [*Sus scrofa*]	*Amphimedon queenslandica* (Demospongiae)	3e-25	77.1	COL1A1 - XP_019854254.1<br>COL5A1 - CAQ63561.1
Collagen I alpha-2 chain [*Sus scrofa domesticus*]	*Amphimedon queenslandica* (Demospongiae)	2e-58	82.2	COL1A1 - XP_019854254.1<br>COL6 - CAQ63562.1
Collagen III alpha-1 chain precursor [*Sus scrofa*]	*Amphimedon queenslandica* (Demospongiae)	8e-39	79.9	Same sequence as after COL1A1 search has the highest match value
Collagen I alpha-1 chain isoform X1 [*Sus scrofa*]	*Aplysina aerophoba* (Demospongiae)	3e-26	83.1	No results with any annotated proteins
Collagen I alpha-2 chain [*Sus scrofa domesticus*]	*Aplysina aerophoba* (Demospongiae)	2e-54	110	COL1A1 - XP_052314686.1
Collagen III alpha-1 chain precursor [*Sus scrofa*]	*Aplysina aerophoba* (Demospongiae)	1e-43	97.3	Negative results (Kinesin-like protein) KAJ7374653.1
Collagen III alpha-1 chain precursor [*Sus scrofa*]	*Chondrosia reniformis* (Demospongiae)	4e-31	64.3	LOW QUALITY PROTEIN: collagen alpha-1(IX) chain-like [Notolabrus celidotus - XP_034534652.1
Collagen I alpha-1 chain isoform X1 [*Sus scrofa*]	*Ephydatia muelleri* (Demospongiae)	4e-31	68.4	No results with any annotated proteins
Collagen I alpha-2 chain [*Sus scrofa domesticus*]	*Ephydatia muelleri* (Demospongiae)	2e-34	72.1	No results with any annotated proteins
Collagen III alpha-1 chain precursor [*Sus scrofa*]	*Ephydatia muelleri* (Demospongiae)	2e-30	58.3	Short-chain collagen C4 [Ephydatia muelleri - P18503.1
Collagen I alpha-1 chain isoform X1 [*Sus scrofa*]	*Halichondria panicea* (Demospongiae)	4e-36	55.1	COL1A1 - XP_019854257.1<br>XP_020906601.1
Collagen I alpha-2 chain [*Sus scrofa domesticus*]	*Halichondria panicea* (Demospongiae)	3e-62	71.2	Collagen alpha-1(XXIV) chain [Geodia barretti] CAI8027724.1
Collagen III alpha-1 chain precursor [*Sus scrofa*]	*Halichondria panicea* (Demospongiae)	2e-47	57.0	Collagen alpha-1(XXIV) chain [Geodia barretti] CAI8027724.1
Any collagens used in query	*Oscarella lobularis* (Homoscleromorpha)	No results with significant similarity		
Any collagens used in query	*Petrosia ficiformis* (Demospongiae)	No results with significant similarity		

Supplementary Table 3 | Aminoacid composition of Spongin vs Collagens (% of residues)

Aminoacids	*Spongia graminae*[8]		*H. communis*[9]	Collagens[10]		
	Spongin A	Spongin B		Rat	Bovine	Codfish
3-hydroxyproline	10.8	9.4	1	9.61	7.84	3.96
4- hydroxyproline			8.7			
Aspartic acid	9.2	9.7	9.4	4.53	3.67	3.88
Threonine	4.3	2.7	2.6	1.88	1.32	1.69
Serine	3.8	2.4	2.5	4.27	3.20	5.39
Glutamic acid	9.5	8.6	7.9	7.33	5.94	5.60
Proline	7.8	7.3	6.7	10.92	8.99	6.27
Glycine	31.5	32.3	31.9	33.31	29.64	26.61
Alanine	5.6	9.4	8.4	11.1	10.2	9.15
Valine	2.9	2.4	3.0	1.71	1.29	1.20
Methionine	0.47	0.31	traces	0.80	0.78	1.50
Isoleucine	2.4	1.7	2.1	0.74	0.67	0.56
Leucine	2.8	2.4	2.7	2.33	1.75	1.65
Tyrosine	0.47	0.4	0.2	0.38	0.15	0.23
Phenylalanine			1	1.46	1.16	1.27
Lysine	0.9	2.4	3.4	2.71	2.22	1.96
Histidine	0.39	0.32	0.4	0.36	0.31	0.50
Arginine	4.7	4.3	4.5	4.22	3.28	3.05
Hydroxylysine	1.2	2.4	2.9	0.93	0.89	0.67
Cystine	0.33	0.6	0.7	0.09	0.12	0.13

Supplementary Table 4 | Average radius of gyration in nm (standard deviation) for simulated models.

	COL-I	COL-III
noXlink	2.46 (0.02)	2.17 (0.02)
1Br	2.46 (0.02)	2.15 (0.01)
2Br	2.44 (0.02)	2.14 (0.02)
3Br	2.45 (0.02)	2.17 (0.01)

Supplementary Table 5 | Average width measurements in nm (standard deviation) for the triple helices of the collagen molecules.

	COL-I	COL-III
noXlink	0.69 (0.08)	0.87 (0.03)
1Br	0.71 (0.09)	0.87 (0.03)
2Br	0.75 (0.10)	0.87 (0.03)
3Br	0.71 (0.08)	0.87 (0.03)

Supplementary Table 6 | Terminal distances between collagen I and III (nm)

System	C-N end	N-C end
noXlink	1.81 (0.19)	2.71 (0.19)
1Br	1.36 (0.12)	2.76 (0.23)
2Br	1.46 (0.10)	4.66 (0.61)
3Br	1.59 (0.14)	4.60 (0.44)

Supplementary Notes

Note 1. Enigmatic collagen microfibrils inside of the skeletal spongin in Demospongiae

In all four sponge classes, the collagenous organic skeleton in the form of the fibrillar collagen is located throughout the mesohyl[11,12]. In contrast to fibrillar collagen, which shows more or less the same structure in all sponges, the spongin skeletal structures are diverse but occur only in demosponges. Spongin is recognized as some kind of collagenous protein[13-15] and in some demosponges (e.g., orders Dictyoceratida, Dendroceratida), it appears as fibers, reaching a thickness of several millimeters.

In previous papers, precise descriptions of the ultrastructural organization of skeletal spongin fibers have already been reported. For example, microfibrils included in spongin were described in *Spongia graminea*[16], *Haliclona rosea*[17], *Ircinia variabilis, Hippospongia communis,* and *Cacospongia scalaris*[18]. The skeletal fibers in *I. variabilis, H. communis,* and *C. scalaris* are composed of fine microfibrils having less than 8 nm diameter and periodic striation with a period of approximately 55 nm[18]. These fibers are comparable to those Gross et al.[16] described in bath sponge *Spongia graminea* with periodic striation of approximately 65 nm. The microfibrils 6-7 nm in diameter with periodical striation approximately 60-65 nm were found enclosed within the perispicular spongin of some Haplosclerida[16,17,19,20]. According to the authors, such microfibrils represent spongin.

In all papers listed above, the authors believed that these skeletal fibers are of collagen origin. For example, Junqua et al.[18] emphasized that the amino acid composition and the glucosylgalactosyl-hydroxylysine content confirm the collagenous nature of these fibers and their high similarity with the collagens from higher vertebrates. However, none of these studies identified the type of collagen to which these collagen fibers or microfibrils could be attributed. Rober Garrone has noted this challenging task as follow: "*It would be of great interest to determine exactly where collagen is located in spongin structures and whether all the spongin assemblies are equivalent.*"[11].

Note 2. Search for Collagen (I) alpha-1,2 and Collagen (III) alpha-1 in Porifera genomes

The translated nucleotide BLAST method was able to identify several sequences in the genomes of wild pig (*Sus scrofa*) and demosponges and homoscleromorph species listed above with significant similarity, which are recorded in Supplementary Table 2.

Summary:

We found COL(I) (alpha-1) homologs in *Amphimedon queenslandica* and *Halichondria panicea* genomes. These homologs were identified as collagens, indicating a close amino acid sequence, but not nucleotide similarity, which is possibly why it was not previously identified.

Genomes used:

Amphimedon queenslandica GCA_000090795.2
https://www.ncbi.nlm.nih.gov/datasets/genome/GCF_000090795.2/
Aplysina aerophoba GCA_949841015.1
https://www.ncbi.nlm.nih.gov/datasets/genome/GCA_949841015.1/
Chondrosia reniformis GCA_947172415.1
https://www.ncbi.nlm.nih.gov/datasets/genome/GCA_947172415.1/
Ephydatia muelleri GCA_013339895.1
https://www.ncbi.nlm.nih.gov/datasets/genome/GCA_013339895.1/
Halichondria panicea GCA_020423275.1
https://www.ncbi.nlm.nih.gov/datasets/genome/GCA_020423275.1/
Oscarella lobularis GCA_947507565.1
https://www.ncbi.nlm.nih.gov/datasets/genome/GCA_947507565.1/
Petrosia ficiformis GCA_947044365.1
https://www.ncbi.nlm.nih.gov/datasets/genome/GCA_947044365.1/

Note 3: Confocal Micro X-ray Fluorescence (CMXRF) measurements of spongin with respect to bromine distribution

The structural properties of spongin allowed providing of the qualitative analysis by CMXRF method in depth of 500 µm, (for details see [21]) especially for the heavier elements like bromine. As it was recently described by us in [21], bromine can be assigned to the spongin structure without doubts. Moreover, the 3D distribution image of bromine (Supplementary Figure 11) provides the most representative reconstruction of the spongin sample.

Note 4. Computational Modeling

Here, we present details of our molecular dynamics (MD) simulation study focused on investigating different dityrosine crosslinks between Collagen type I and type III, involving bromine atoms.

Our simulation aimed to investigate the molecular mechanisms behind the effects of the experimentally determined crosslinkins on the structural integrity of spongin. We modeled spongin as a complex involving Collagen I and III. To address the limitations associated with simulating collagen triple helices, which may not fully capture their interactions within the complex environment or with neighboring helices, we opted to model small crosslink assemblies of collagen. This approach has been shown to provide a valuable perspective on how collagen behaves under various conditions, reflecting the significant role that small assemblies and crosslinks play in determining the mechanical integrity and deformation behavior of collagen fibrils, as previously showed[22,23].

We initiated our study by constructing the collagen types I and III complex, capitalizing on the pre-existing hydrophobic groups. Specifically, we leveraged phenylalanine residues (later replaced with TYR) as interaction points within the sequences. Our computational investigation included 51 ns of MD simulations across four distinct systems: no crosslink, 1Br, 2Br, and 3Br crosslinks. For these simulations, we used the TIP3P water model, chosen for its computational efficiency and accuracy in capturing the relevant collagen protein dynamics, as supported by recent studies on similar systems[24,25].

We observed minimal fluctuations in the radius of gyration values across the different systems, indicating that our MD simulations were highly stable. The Rg values for both COL-I and COL-III remained fairly consistent, with no significant deviations (see Supplementary Table 4). This consistency indicates well-modeled systems with similar results to previous computational and experimental data[26].

Supplementary Table 5 shows the computed average width of the collagen molecules.

While COL-III retains its width relatively well, the introduction of bromide crosslinks changes the width of COL-I. This could be related to the stability of the collagen fibrils[27]. This disparity in response between collagens I and III suggests that bromide crosslinks have a more substantial effect on the structural integrity of collagen I, potentially leading to increased structural stability.

Supplementary Table 6 and Figure 5 in the main text display the terminal distances for the different systems. These distances reflect the spatial arrangement of the collagen triple helices and offer valuable information about their overall structural changes.

The differences in the N- and C-terminal distances between the two triple helices indicate that the presence of bromide crosslinks significantly alters the spatial arrangement of COL-I and COL-III during assembly. This could have consequences for the overall stability and mechanical properties of the collagen-based biocomposite, potentially leading to enhanced load-bearing capabilities. Understanding these region-specific effects can aid in the design of collagen-based materials with tailored properties for various applications.

Our MD simulations align with the notion that bromine, when strategically positioned within the collagen chain, increases its flexibility, with a more pronounced effect on COL-I. This insight suggests that in fibrils primarily composed of COL-I, bromide crosslinks would be highly effective in stiffening the structure. However, further exploration is warranted to elucidate potential variations in the impact of crosslink positioning within the collagen chains, especially at the termini. Our study underscores the significant increase in flexibility observed in the complex, surpassing the combined impact of individual collagen molecules without crosslinks.

While our simulations offer insights into the distinctions between collagen I and III, particularly with regard to their response to bromine crosslinks, these findings must be contextualized within the broader landscape of experimental observations. Real collagen molecules are found in bundles, i.e., in a fibrillar structure, a structure not explored in our simplified model.

Supplementary References.

1. Grüner, D. *et al.* Irregular Shaped, Assumably Semi-Crystalline Calciumphosphate Platelet Deposition at the Mineralization Front of Rabbit Femur Osteotomy: A HR-TEM Study. *Scanning* **35**, 169–182 (2013). https://doi.org/10.1002/sca.21043.

2. Simon, P. *et al.* First evidence of octacalcium phosphate@osteocalcin nanocomplex as skeletal bone component directing collagen triple-helix nanofibril mineralization. *Sci. Rep.* **8**, 13696 (2018). https://doi.org/10.1038/s41598-018-31983-5.

3. Simon, P. *et al.* Podosome-Driven Defect Development in Lamellar Bone under the Conditions of Senile Osteoporosis Observed at the Nanometer Scale. *ACS Biomater. Sci. Eng.* **7**, 2255–2267 (2021). https://doi.org/10.1021/acsbiomaterials.0c01493.

4. Talari, A. C. S., Movasaghi, Z., Rehman, S. & Rehman, I. U. Raman Spectroscopy of Biological Tissues. *Appl. Spectrosc. Rev.* **50**, 46–111 (2015). https://doi.org/10.1080/05704928.2014.923902.

5. Nguyen, T. T. *et al.* Characterization of type I and IV collagens by Raman microspectroscopy: Identification of spectral markers of the dermo-epidermal junction. *Spectrosc. (New York)* **27**, 421–427 (2012). https://doi.org/10.1155/2012/686183.

6. Martinez, M. G., Bullock, A. J., MacNeil, S. & Rehman, I. U. Characterisation of structural changes in collagen with Raman spectroscopy. *Appl. Spectrosc. Rev.* **54**, 509–542 (2019). https://doi.org/10.1080/05704928.2018.1506799.

7. Cárcamo, J. J., Aliaga, A. E., Clavijo, R. E., Brañes, M. R. & Campos-Vallette, M. M. Raman study of the shockwave effect on collagens. *Spectrochim. Acta. A. Mol. Biomol. Spectrosc.* **86**, 360–365 (2012). https://doi.org/10.1016/j.saa.2011.10.049.

8. Plez. K. A. & Gross. J. The amino acid composition and morphology of some invertebrate and vertebrate collagens. Biochim. Biophys. Acta 34. 24–39 (1959). https://doi.org/10.1016/0006-3002(59)90229-x.

9. Junqua. S.. Robert. L.. Garrone. R.. De Ceccatty. M. P. & Vacelet. J. Biochemical and Morphological Studies on Collagens of Horny Sponges. Ircinia Filaments Compared to Spongines. Connect. Tissue Res. 2. 193–203 (1974). https://doi.org/10.3109/03008207409152244.

10. Carvalho. A. M.. Marques. A. P.. Silva. T. H. & Reis. R. L. Evaluation of the Potential of Collagen from Codfish Skin as a Biomaterial for Biomedical Applications. *Mar. Drugs 2018. Vol. 16. Page 495* **16**. 495 (2018). https://doi.org/10.3390/md16120495.

11. Garrone, R. The Collagen of the Porifera. in *Biology of Invertebrate and Lower Vertebrate Collagens* (1985). https://doi.org/10.1007/978-1-4684-7636-1_12.

12. Simpson, T. L. *The Cell Biology of Sponges. The Cell Biology of Sponges* (Springer Verlag, 1984). https://doi.org/10.1007/978-1-4612-5214-6.

13. Exposito, J. Y., Cluzel, C., Garrone, R. & Lethias, C. Evolution of collagens. *Anat. Rec.* **268**, 302–316 (2002). https://doi.org/10.1002/ar.10162.

14. Jesionowski, T. *et al.* Marine spongin: Naturally prefabricated 3D scaffold-based biomaterial. *Mar. Drugs* **16**, (2018). https://doi.org/10.3390/md16030088.

15. Ehrlich, H. *Marine Biological Materials of Invertebrate Origin.* (Springer International Publishing, 2019). https://doi.org/10.1007/978-3-319-92483-0.

16. Gross, J., Sokal, Z. & Rougvie, M. Structural and chemical studies on the connective tissue of marine sponges. *J.Histochem.Cytochem.* **4**, 227-246 (1956). https://doi.org/10.1177/4.3.227.

17. Garrone, R. Collagène, spongine et squelette minéral chez l'éponge Haliclonarosea (O.S.) (Démosponge, Haploscléride). *J. Microsc.* **8** : 581-598 (1969).

18. Junqua, S., Robert, L., Garrone, R., De Ceccatty, M. P. & Vacelet, J. Biochemical and Morphological Studies on Collagens of Horny Sponges. Ircinia Filaments Compared to Spongines. *Connect. Tissue Res.* **2**, 193–203 (1974). https://doi.org/10.3109/03008207409152244.

19. Garrone, R. *Phylogenesis of connective tissue.* (Karger, Basel, 1978).

20. Garrone, R., & Pottu, J. Collagen biosynthesis in sponges: Elaboration of spongin by spongocytes. *J. Submicros. Cytol.* **5** : 199-218. (1973).

21. Kubiak, A. et al. Spongin as a Unique 3D Template for the Development of Functional Iron-Based Composites Using Biomimetic Approach In Vitro. *Mar. Drugs.* **21**, 460 (2023). https://doi.org/10.3390/md21090460.

22. Hu, J. *et al.* Design of synthetic collagens that assemble into supramolecular banded fibers as a functional biomaterial testbed. *Nat. Commun.* **13**, 6761 (2022). https://doi.org/10.1038/s41467-022-34127-6.

23. Bourne, J. W. & Torzilli, P. A. Molecular simulations predict novel collagen conformations during cross-link loading. *Matrix Biol.* **30**, 356–360 (2011). https://doi.org/10.1016/j.matbio.2011.03.010.

24. Vassaux, M. Heterogeneous Structure and Dynamics of Water in a Hydrated Collagen Microfibril *Biomacromolecules*, **25**, 4809–4818 (2024). https://doi.org/10.1021/acs.biomac.4c00183.

25. de Alcântara, A. C. S. et al. The Role of the Extrafibrillar Volume on the Mechanical Properties of Molecular Models of Mineralized Bone Microfibrils. *ACS Biomater Sci Eng.* **9**, 230-245. (2023). https://doi.org/10.1021/acsbiomaterials.2c00728.

26. Walker, K. T. *et al.* Non-linearity of the collagen triple helix in solution and implications for collagen function. *Biochem. J.* **474**. 2203–2217 (2017). https://doi.org/10.1042/BCJ20170217.

27. Raman, S. S.. Parthasarathi, R., Subramanian, Y. & Ramasami, T. Role of length-dependent stability of collagen-like peptides. *J. Phys. Chem. B* **112**, 1533–1539 (2008) https://doi.org/10.1021/jp0728297.
