# Supplementary material for: Discovery of mammalian collagens I and III within ancient poriferan biopolymer spongin
Source: Nat Commun. 2025 Mar 13;16:2515. doi: 10.1038/s41467-025-57460-y (PMC11906918; doi:10.1038/s41467-025-57460-y)
Supplement: Supplementary file 2 — Description of Additional Supplementary Files [file 41467_2025_57460_MOESM2_ESM.pdf]

Description of Additional Supplementary Files

File Name: Supplementary Data 1

Description: Detailed protein report: MASCOT search and LCMS/MS analysis results
