# Supplementary material for: Discovery of mammalian collagens I and III within ancient poriferan biopolymer spongin
Source: Nat Commun. 2025 Mar 13;16:2515. doi: 10.1038/s41467-025-57460-y (PMC11906918; doi:10.1038/s41467-025-57460-y)
Supplement: Supplementary file 3 — Supplementary Data 1 [file 41467_2025_57460_MOESM3_ESM.pdf]

Detailed Protein Report

Mascot search results for SDS-PAAG samples 210723 A, 210723 B, 210723 C

(see also Figure 3 in main text)

Detailed Protein Report

Project Info

**Name:**	Spongin	**Date:**	February 26, 2018

Sample Info &Protocols | **Date:** September 24, 2021

**Name:**	210723 A

SearchResult Info

SearchResult	Location	Search Engine	Database	Ident. Compounds
NCBI-collagen-2020_2021-09-25 07:55:31	/Spongin/SponginA 2021/210723 A_C4_01_5080.d	Mascot, 2.3.0	NCBI_Collagen_2020, NCBI_Collagen_D20200704_collagenonly_final.fasta	47/4066

Detailed Protein Report

**Protein 1:**	collagen alpha-1(I) chain isoform X1 [Sus scrofa]		
**Accession:**	XP_020922812.1	**Score:**	1557.46
**Database:**	NCBI_Collagen_2020	**MW [kDa]:**	139.20
**Seq. Coverage [%]:**	23.70 %	**pI:**	5.50
		**No. of Peptides:**	23
**Modification(s):**	Oxidation, Deamidated		

10	20	30	40	50	60	70	80	90	100	110	120
MFSFVDLRLL	LLLAATALLT	HGQEEGQEEG	QQGQEEDIPP	VTCVQNGLRY	HDRDVWKPVP	CQICVCDNGN	VLCDDVICDE	IKNCPSARVP	AGECCPVCPE	GEVSPTDQET	TGVEGPKGDT
130	140	150	160	170	180	190	200	210	220	230	240
GPRGPRGPSG	PPGRDGIPGQ	PGLPGPPGPP	GPPGPPGLGG	NFAPQLSYGY	DEKSAGISVP	GPMGPSGPRG	LPGPPGAPGP	QGFQGPPGEP	GEPGASGPMG	PRGPPGPPGK	NGDDGEAGKP
250	260	270	280	290	300	310	320	330	340	350	360
GRPGERGPPG	PQGARGLPGT	AGLPGMKGHR	GFSGLDGAKG	DAGPAGPKGE	PGSPGENGAP	GQMGPRGLPG	ERGRPGPPGP	AGARGNDGAT	GAAGPPGPTG	PAGPPGFPGA	VGAKGEAGPQ
370	380	390	400	410	420	430	440	450	460	470	480
GARGSEGPQG	VRGEPGPPGP	AGAAGPAGNP	GADGQPGGKG	ANGAPGIAGA	PGFPGARGPS	GPQGPSGPPG	PKGNSGEPGA	PGSKGDTGAK	GEPGPTGVQG	PPGPAGEEGK	RGARGEPGPA
490	500	510	520	530	540	550	560	570	580	590	600
GLPGPPGERG	GPGSRGFPGA	DGVAGPKGPA	GERGSPGPAG	PKGSPGEAGR	PGEAGLPGAK	GLTGSPGSPG	PDGKTGPPGP	AGQDGRPGPP	GPPGARGQAG	VMGFPGPKGA	AGEPGKAGER
610	620	630	640	650	660	670	680	690	700	710	720
GVPGPPGAVG	PAGKDGEAGA	QGPPGPAGPA	GERGEQGPAG	SPGFQGLPGP	AGPPGEAGKP	GEQGVPGDLG	APGPSGARGE	RGFPGERGVQ	GPPGPAGPRG	ANGAPGNDGA	KGDAGAPGAP
730	740	750	760	770	780	790	800	810	820	830	840
GSQGAPGLQG	MPGERGAAGL	PGPKGDRGDA	GPKGADGAPG	KDGVRGLTGP	IGPPGPAGAP	GDKGETGPSG	PAGPTGARGA	PGDRGEPGPP	GPAGFAGPPG	ADGQPGAKGE	PGDAGAKGDA
850	860	870	880	890	900	910	920	930	940	950	960
GPPGPAGPTG	PPGPIGSVGA	PGPKGARGSA	GPPGATGFPG	AAGRVGPPGP	SGNAGPPGPP	GPAGKEGSKG	PRGETGPAGR	PGEAGPPGPP	GPAGEKGSPG	ADGPAGAPGT	PGPQGIAGQR
970	980	990	1000	1010	1020	1030	1040	1050	1060	1070	1080
GVVGLPGQRG	ERGFPGLPGP	SGEPGKQGPS	GPSGERGPPG	PMGPPGLAGP	PGESGREGAP	GAEGSPGRDG	APGPKGDRGE	SGPAGPPGAP	GAPGAPGPVG	PAGKSGDRGE	TGPAGPAGPV
1090	1100	1110	1120	1130	1140	1150	1160	1170	1180	1190	1200
GPVGARGPAG	PQGPRGDKGE	TGEQGDRGIK	GHRGFSGLQG	PPGPPGSPGE	QGPSGASGPA	GPRGPPGSAG	APGKDGLNGL	PGPIGPPGPR	GRTGDAGPVG	PPGPPGPPGP	PGPPSGGFDF
1210	1220	1230	1240	1250	1260	1270	1280	1290	1300	1310	1320
SFLPQPPQEK	AHDGGRYYRA	DDANVVRDRD	LEVDTTLKSL	SQQIENIRSP	EGSRKNPART	CRDLKMCHSD	WKSGEYWIDP	NQGCNLDAIK	VFCNMETGET	CVYPTQPSVP	QKNWYISKNP
1330	1340	1350	1360	1370	1380	1390	1400	1410	1420	1430	1440
KDKRHVWYGE	SMTDGFQFEY	GGEGSDPADV	AIQLTFLRLM	STEASQNITY	HCKNSVAYMD	QQTGNLKKAL	LLQGSNEIEI	RAEGNSRFTY	SVIYDGCTSH	TGAWGKTVIE	YKTTKTSRLP
1450	1460	1470									
IIDVAPLDVG	APDQEFGIDL	SPVCFL									

Detailed Protein Report

Cmpd.	No. of Cmpds.	m/z meas.	Δ m/z [ppm]	z	Rt [min]	Score	P	Range	Sequence	Modification
206	2	880.3650	2.23	2	19.2	49.2	0	289-306	K.GEPGSPGENGAPGQMGPR.G	Oxidation: 3, 6, 12, 17; Deamidated: 9
124	2	374.5333	3.73	3	17.0	39.6	0	313-324	R.GRPGPPGPAGAR.G	Oxidation: 3, 6
1371	2	793.8823	1.00	2	39.0	50.4	0	400-417	K.GANGAPGIAGAPGFPGAR.G	Oxidation: 6, 12, 15; Deamidated: 3
412	2	666.8324	2.45	2	23.3	110.8	0	418-432	R.GPSGPQGPSGPPGPK.G	Oxidation: 12
930	2	718.3460	2.46	2	31.9	63.2	0	475-489	R.GEPGPAGLPGPPGER.G	Oxidation: 3, 9, 12
1063	1	544.7729	1.31	2	33.8	67.8	0	496-507	R.GFPGADGVAGPK.G	Oxidation: 3
354	2	552.6057	2.61	3	22.3	49.4	0	523-540	K.GSPGEAGRPGEAGLPGAK.G	Oxidation: 3, 9, 15
274	3	629.8005	2.15	2	20.7	88.7	0	541-554	K.GLTGSPGSPGPDGK.T	Oxidation: 6, 9
487	2	686.3330	2.45	3	24.5	27.4	0	555-576	K.TGPPGPAGQDGRPGPPGPPGAR.G	Oxidation: 3, 16, 18, 19
627	2	596.8207	1.68	2	26.8	64.1	0	601-614	R.GVPGPPGAVGPAGK.D	Oxidation: 3, 5
451	2	553.2920	1.69	2	24.0	31.4	0	688-699	R.GVQGPPGPAGPR.G	Oxidation: 8
445	2	553.2920	1.75	2	23.9	44.4	0	688-699	R.GVQGPPGPAGPR.G	Oxidation: 6
658	1	738.9964	2.97	3	27.3	59.4	0	712-735	K.GDAGAPGAPGSQGAPGLQGMPGER.G	Oxidation: 6, 9, 15, 20, 21
1396	1	961.8145	1.97	3	39.4	41.5	1	766-798	R.GLTGPIGPPGPAGAPGDKGETGPSGPAGPTGAR.G	Oxidation: 11, 15
417	2	656.3194	2.14	2	23.4	83.6	0	784-798	K.GETGPSGPAGPTGAR.G	
962	2	730.3507	1.14	2	32.3	113.9	0	868-884	R.GSAGPPGATGFPGAAGR.V	Oxidation: 6, 12
839	2	449.7597	1.85	2	30.2	39.6	0	961-969	R.GVVGLPGQR.G	Oxidation: 6
873	1	724.3531	3.80	3	30.7	43.0	0	1039-1064	R.GESGPAGPPGAPGAPGAPGPVGPAGK.S	Oxidation: 9, 12, 15, 18
975	2	654.6659	2.28	3	32.5	78.6	1	1065-1086	K.SGDRGETGPAGPAGPVGPVGAR.G	
1150	2	773.9033	1.37	2	35.3	97.3	0	1069-1086	R.GETGPAGPAGPVGPVGAR.G	
1593	1	902.4263	3.41	3	42.7	88.5	0	1114-1143	R.GFSGLQGPPGPPGSPGEQGPSGASGPAGPR.G	Oxidation: 11, 12, 15
1688	1	781.4027	0.80	2	44.3	43.4	0	1155-1170	K.DGLNGLPGPIGPPGPR.G	Oxidation: 7, 13, 15
1690	1	781.8954	1.69	2	44.4	57.5	0	1155-1170	K.DGLNGLPGPIGPPGPR.G	Oxidation: 7, 13, 15; Deamidated: 4

Detailed Protein Report

Protein 2: alpha2 chain of type I collagen [Sus scrofa domesticus]

**Accession:**	BAX02569.1	**Score:**	640.97
**Database:**	NCBI_Collagen_2020	**MW [kDa]:**	129.10
**Seq. Coverage [%]:**	9.80 %	**pI:**	9.83
		**No. of Peptides:**	9
**Modification(s):**	Oxidation		

10	20	30	40	50	60	70	80	90	100	110	120
MLSFVDTRTL	LLLAVTSCLA	TCQSLQEATA	RKGPTGDRGP	RGERGPPGPP	GRDGDDGIPG	PPGPPGPPGP	PGLGGNFAAQ	YDGKGVGAGP	GPMGLMGPRG	PPGAVGAPGP	QGFQGPAGEP
130	140	150	160	170	180	190	200	210	220	230	240
GEPGQTGPAG	ARGPPGPPGK	AGEDGHPGKP	GRPGERGVVG	PQGARGFPGT	PGLPGFKGIR	GHNGLDGLKG	QPGAPGVKGE	PGAPGENGTP	GQTGARGLPG	ERGRVGAPGP	AGARGNDGSV
250	260	270	280	290	300	310	320	330	340	350	360
GPVGPAGPIG	SAGPPGFPGA	PGPKGELGPV	GNPGPAGPAG	PRGEVGLPGV	SGPVGPPGNP	GANGLPGAKG	AAGLPGVAGA	PGLPGPRGIP	GPAGAAGATG	ARGLVGEPGP	AGSKGESGNK
370	380	390	400	410	420	430	440	450	460	470	480
GEPGAAGPQG	PPGPSGEEGK	RGPNGEVGSA	GPPGPPGLRG	NPGSRGLPGA	DGRAGVMGPP	GSRGPTGPAG	VRGPNGDSGR	PGEPGLMGPR	GFPGSPGNVG	PAGKEGPAGL	PGIDGRPGPI
490	500	510	520	530	540	550	560	570	580	590	600
GPAGARGEPG	NIGFPGPKGP	TGDPGKNGEK	GHAGLAGARG	APGPDGNNGA	QGPPGPQGVQ	GGKGEQGPAG	PPGFQGLPGP	AGTAGEVGKP	GERGIPGEFG	LPGPAGPRGE	RGPPGESGAA
610	620	630	640	650	660	670	680	690	700	710	720
GPAGPIGSRG	PSGPPGPDGN	KGEPGVLGAP	GTAGPSGPSG	LPGERGAAGI	PGGKGEKGET	GLRGDVGSPG	RDGARGAPGA	VGAPGPAGAN	GDRGEAGPAG	PAGPAGPRGS	PGERGEVGPA
730	740	750	760	770	780	790	800	810	820	830	840
GPNGFAGPAG	AAGQPGAKGE	RGTKGPKGEN	GPVGPTGPVG	AAGPAGPNGP	PGPAGSRGDG	GPPGATGFPG	AAGRIGPPGP	SGISGPPGPP	GPAGKEGLRG	PRGDQGPVGR	TGETGASGPP
850	860	870	880	890	900	910	920	930	940	950	960
GFAGEKGPSG	EPGTAGPPGT	PGPQGILGAP	GFLGLPGSRG	ERGLPGVAGS	VGEPGPLGIA	GPPGARGPPG	AVGNPGVNGA	PGEAGRDGNP	GSDGPPGRDG	QAGHKGERGY	PGNPGPAGAA
970	980	990	1000	1010	1020	1030	1040	1050	1060	1070	1080
GAPGPQGAVG	PAGKHGNRGE	PGPAGSVGPA	GAVGPRGPSG	PQGIRGEKGE	PGDKGPRGLP	GLKGHNGLQG	LPGLAGHHGD	QGAPGPVGPA	GPRGPAGPSG	PAGKDGRTGQ	PGAVGPAGIR
1090	1100	1110	1120	1130	1140	1150	1160	1170	1180	1190	1200
GSQGSQGPAG	PPGPPGPPGP	PGPSGGGYDF	GYEGDFYRAD	QPRSPPSLRP	KDYEVDATLK	SLNNQIETLL	TPEGSRKNPA	RTCRDLRLSH	PEWSSGYYWI	DPNQGCTMDA	IKVYCDFSTG
1210	1220	1230	1240	1250	1260	1270	1280	1290	1300	1310	1320
ETCIRAQPEN	IPAKNWYRNS	KVKKHVWLGE	TINGGTQFEY	NMEGVTTKEM	ATQLAFMRLL	ANHASQNITY	HCKNSIAYMD	EETGNLKKAV	ILQGSNDVEL	VAEGNSRFTY	TVLVDGCSKK
1330	1340	1350	1360	1370							
TNEWRKTIIE	YKTNKPSRLP	ILDIAPLDIG	DADQEVSVDV	GPVCFK							

Detailed Protein Report

Cmpd.	No. of Cmpds.	m/z meas.	Δ m/z [ppm]	z	Rt [min]	Score	P	Range	Sequence	Modification
772	2	620.3267	1.73	2	29.1	47.3	0	328-342	R.GIPGPAGAAGATGAR.G	Oxidation: 5
773	2	637.3136	2.26	2	29.1	76.3	0	451-464	R.GFPGSPGNVGPAGK.E	Oxidation: 3, 6
1911	2	727.3766	1.68	2	48.1	31.3	0	574-588	R.GIPGEFGLPGPAGPR.G	Oxidation: 3, 14
848	2	775.8823	1.05	2	30.3	128.0	0	592-609	R.GPPGESGAAGPAGPIGSR.G	Oxidation: 3
568	1	631.3180	0.35	2	25.9	84.0	0	694-708	R.GEAGPAGPAGPAGPR.G	
973	2	737.3406	1.54	2	32.5	66.6	0	778-794	R.GDGGPPGATGFPGAAGR.I	Oxidation: 6, 12
1076	2	774.8930	1.48	2	34.1	60.0	0	979-996	R.GEPGPAGSVGPAGAVGPR.G	Oxidation: 5
376	2	434.7366	2.71	2	22.7	43.9	0	997-1005	R.GPSGPQGIR.G	
782	1	598.8229	0.31	2	29.3	34.5	0	1068-1080	R.TGQPGAVGPAGIR.G	Oxidation: 4

Detailed Protein Report

ProjectInfo

**Name:**	Spongin	**Date:**	February26,2018

SampleInfo&Protocols

		**Date:**	September24,2021
**Name:**	210723 B		

SearchResultInfo

SearchResult		Location	SearchEngine	Database	Ident.Compounds
NCBI-collagen-2020_2021-09-25	19:53:39	/Spongin/SponginB2021/210723B_C5_01_5081.d	Mascot,2.3.0	NCBI_Collagen_2020, NCBI_Collagen_D202007	267/3744

Protein1: collagenalpha-1(I)chainisoformX1[Susscrofa]

**Accession:**	XP_020922812.1	**Score:**	6511.94
**Database:**	NCBI_Collagen_2020	**MW[kDa]:**	139.20
**Seq.Coverage[%]:**	50.20%	**pI:**	5.50
		**No.ofPeptides:**	112
**Modification(s):**	Oxidation,Deamidated		

Detailed Protein Report

10	20	30	40	50	60	70	80	90	100	110	120
MFSFVDLRLL	LLLAATALLT	HGQEEGQEEG	QQGQEEDIPP	VTCVQNGLRY	HDRDVWKPVP	CQICVCDNGN	VLCDDVICDE	IKNCPSARVP	AGECCPVCPE	GEVSPTDQET	TGVEGPKGDT
130	140	150	160	170	180	190	200	210	220	230	240
GPRGPRGPSG	PPGRDGIPGQ	PGLPGPPGPP	GPPGPPGLGG	NFAPQLSYGY	DEKSAGISVP	GPMGPSGPRG	LPGPPGAPGP	QGFQGPPGEP	GEPGASGPMG	PRGPPGPPGK	NGDDGEAGKP
250	260	270	280	290	300	310	320	330	340	350	360
GRPGERGPPG	PQGARGLPGT	AGLPGMKGHR	GFSGLDGAKG	DAGPAGPKGE	PGSPGENGAP	GQMGPRGLPG	ERGRPGPPGP	AGARGNDGAT	GAAGPPGPTG	PAGPPGFPGA	VGAKGEAGPQ
370	380	390	400	410	420	430	440	450	460	470	480
GARGSEGPQG	VRGEPGPPGP	AGAAGPAGNP	GADGQPGGKG	ANGAPGIAGA	PGFPGARGPS	GPQGPSGPPG	PKGNSGEPGA	PGSKGDTGAK	GEPGPTGVQG	PPGPAGEEGK	RGARGEPGPA
490	500	510	520	530	540	550	560	570	580	590	600
GLPGPPGERG	GPGSRGFPGA	DGVAGPKGPA	GERGSPGPAG	PKGSPGEAGR	PGEAGLPGAK	GLTGSPGSPG	PDGKTGPPGP	AGQDGRPGPP	GPPGARGQAG	VMGFPGPKGA	AGEPGKAGER
610	620	630	640	650	660	670	680	690	700	710	720
GVPGPPGAVG	PAGKDGEAGA	QGPPGPAGPA	GERGEQGPAG	SPGFQGLPGP	AGPPGEAGKP	GEQGVPGDLG	APGPSGARGE	RGFPGERGVQ	GPPGPAGPRG	ANGAPGNDGA	KGDAGAPGAP
730	740	750	760	770	780	790	800	810	820	830	840
GSQGAPGLQG	MPGERGAAGL	PGPKGDRGDA	GPKGADGAPG	KDGVRGLTGP	IGPPGPAGAP	GDKGETGPSG	PAGPTGARGA	PGDRGEPGPP	GPAGFAGPPG	ADGQPGAKGE	PGDAGAKGDA
850	860	870	880	890	900	910	920	930	940	950	960
GPPGPAGPTG	PPGPIGSVGA	PGPKGARGSA	GPPGATGFPG	AAGRVGPPGP	SGNAGPPGPP	GPAGKEGSKG	PRGETGPAGR	PGEAGPPGPP	GPAGEKGSPG	ADGPAGAPGT	PGPQGIAGQR
970	980	990	1000	1010	1020	1030	1040	1050	1060	1070	1080
GVVGLPGQRG	ERGFPGLPGP	SGEPGKQGPS	GPSGERGPPG	PMGPPGLAGP	PGESGREGAP	GAEGSPGRDG	APGPKGDRGE	SGPAGPPGAP	GAPGAPGPVG	PAGKSGDRGE	TGPAGPAGPV
1090	1100	1110	1120	1130	1140	1150	1160	1170	1180	1190	1200
GPVGARGPAG	PQGPRGDKGE	TGEQGDRGIK	GHRGFSGLQG	PPGPPGSPGE	QGPSGASGPA	GPRGPPGSAG	APGKDGLNGL	PGPIGPPGPR	GRTGDAGPVG	PPGPPGPPGP	PGPPSGGFDF
1210	1220	1230	1240	1250	1260	1270	1280	1290	1300	1310	1320
SFLPQPPQEK	AHDGGRYYRA	DDANVVRDRD	LEVDTTLKSL	SQQIENIRSP	EGSRKNPART	CRDLKMCHSD	WKSGEYWIDP	NQGCNLDAIK	VFCNMETGET	CVYPTQPSVP	QKNWYISKNP
1330	1340	1350	1360	1370	1380	1390	1400	1410	1420	1430	1440
KDKRHVWYGE	SMTDGFQFEY	GGEGSDPADV	AIQLTFLRLM	STEASQNITY	HCKNSVAYMD	QQTGNLKKAL	LLQGSNEIEI	RAEGNSRFTY	SVIYDGCTSH	TGAWGKTVIE	YKTTKTSRLP
1450	1460	1470									

Detailed Protein Report

Cmpd.	No. ofCmpds.	m/zmeas.	Δ m/z[ppm]	z	Rt[min]	Score	P	Range	Sequence	Modification
874	2	581.7944	3.61	2	32.9	58.5	0	256-267	R.GLPGTAGLPGMK.G	Oxidation:3,9,11,12
864	2	434.2148	2.00	2	32.8	49.3	0	271-279	R.GFSGLDGAK.G	Oxidation:9
912	2	426.2174	1.96	2	33.6	47.1	0	271-279	R.GFSGLDGAK.G	
958	2	539.9350	2.32	3	34.5	97.6	1	271-288	R.GFSGLDGAKGDAGPAGPK.G	Oxidation:9
325	3	842.3730	3.46	3	22.7	60.5	1	280-306	K.GDAGPAGPKGEPGSPGENGAPGQMGPR.G	Oxidation:9,12,15,21,24
158	2	879.8721	1.18	2	18.9	70.6	0	289-306	K.GEPGSPGENGAPGQMGPR.G	Oxidation:3,6,12,15
195	2	880.3642	1.29	2	19.8	57.3	0	289-306	K.GEPGSPGENGAPGQMGPR.G	Oxidation:3,6,12,17; Deamidated:9
172	2	587.2463	3.09	3	19.2	69.5	0	289-306	K.GEPGSPGENGAPGQMGPR.G	Oxidation:3,6,12,15; Deamidated:9
200	2	880.3644	1.54	2	19.9	51.1	0	289-306	K.GEPGSPGENGAPGQMGPR.G	Oxidation:3,6,15,17; Deamidated:9
93	2	561.2951	1.72	2	16.9	43.4	0	313-324	R.GRPGPPGPAGAR.G	Oxidation:3,6
73	4	374.5323	1.30	3	16.2	36.8	0	313-324	R.GRPGPPGPAGAR.G	Oxidation:5,6
101	4	374.5330	2.96	3	17.3	32.7	0	313-324	R.GRPGPPGPAGAR.G	Oxidation:3,5
1334	2	850.0758	2.56	3	41.8	38.4	0	325-354	R.GNDGATGAAGPPGPTGPAGPPGFPGAVGAK .G	Oxidation:11,24,30
1340	2	850.0752	1.88	3	41.9	20.4	0	325-354	R.GNDGATGAAGPPGPTGPAGPPGFPGAVGAK .G	Oxidation:17,20,21
1303	3	855.4079	3.16	3	41.2	90.2	0	325-354	R.GNDGATGAAGPPGPTGPAGPPGFPGAVGAK .G	Oxidation:11,21,24,30
598	1	762.6865	2.97	3	27.6	35.0	0	373-399	R.GEPGPPGPAGAAGPAGNPGADGQPGGK.G	Oxidation:3,5,27
575	3	768.0181	2.88	3	27.1	45.8	0	373-399	R.GEPGPPGPAGAAGPAGNPGADGQPGGK.G	Oxidation:3,5,24,27
524	2	768.0179	2.62	3	26.2	27.2	0	373-399	R.GEPGPPGPAGAAGPAGNPGADGQPGGK.G	Oxidation:6,8,14,18
430	2	773.3504	3.73	3	24.6	62.4	0	373-399	R.GEPGPPGPAGAAGPAGNPGADGQPGGK.G	Oxidation:6,8,14,18,24
1188	4	793.8832	2.08	2	38.9	68.5	0	400-417	K.GANGAPGIAGAPGFPGAR.G	Oxidation:6,12,15; Deamidated:3
1146	2	793.3904	1.15	2	38.1	37.1	0	400-417	K.GANGAPGIAGAPGFPGAR.G	Oxidation:6,12,15
397	4	666.8321	2.03	2	24.0	115.2	0	418-432	R.GPSGPQGPSGPPGPK.G	Oxidation:12

Detailed Protein Report

Cmpd.	No. ofCmpds.	m/zmeas.	Δ m/z[ppm]	z	Rt[min]	Score	P	Range	Sequence	Modification
352	4	674.8299	2.42	2	23.2	126.3	0	418-432	R.GPSGPQGPSGPPGPK.G	Oxidation:12,14
219	5	674.8294	1.79	2	20.3	105.0	0	418-432	R.GPSGPQGPSGPPGPK.G	Oxidation:8,11
301	2	674.8291	1.34	2	22.1	83.4	0	418-432	R.GPSGPQGPSGPPGPK.G	Oxidation:2,15
281	1	682.8271	2.03	2	21.7	53.0	0	418-432	R.GPSGPQGPSGPPGPK.G	Oxidation:2,14,15
587	2	799.0406	2.70	3	27.4	74.9	1	445-470	K.GDTGAKGEPGPTGVQGPPGPAGEEGK.R	Oxidation:6,9,26
586	2	799.0410	3.14	3	27.3	48.7	1	445-470	K.GDTGAKGEPGPTGVQGPPGPAGEEGK.R	Oxidation:9,11,17
267	1	634.7978	-8.45	4	21.3	24.5	2	445-471	K.GDTGAKGEPGPTGVQGPPGPAGEEGKR.G	Oxidation:18,20;Deamidated: 15
512	2	638.5586	4.24	4	26.0	79.1	2	445-471	K.GDTGAKGEPGPTGVQGPPGPAGEEGKR.G	Oxidation:9,11,17
679	3	925.4332	1.52	2	29.3	84.0	0	451-470	K.GEPGPTGVQGPPGPAGEEGK.R	Oxidation:3,12
693	2	925.4328	1.09	2	29.5	42.6	0	451-470	K.GEPGPTGVQGPPGPAGEEGK.R	Oxidation:5,14
790	1	917.4353	1.11	2	31.5	51.4	0	451-470	K.GEPGPTGVQGPPGPAGEEGK.R	Oxidation:12
665	2	663.9946	3.46	3	29.0	84.6	1	451-471	K.GEPGPTGVQGPPGPAGEEGKR.G	Oxidation:12
572	4	669.3257	2.61	3	27.1	72.9	1	451-471	K.GEPGPTGVQGPPGPAGEEGKR.G	Oxidation:5,12
777	4	718.3453	1.42	2	31.2	76.4	0	475-489	R.GEPGPAGLPGPPGER.G	Oxidation:3,9,12
822	4	718.3455	1.81	2	32.1	51.1	0	475-489	R.GEPGPAGLPGPPGER.G	Oxidation:3,11,12
903	2	552.7710	2.41	2	33.4	56.4	0	496-507	R.GFPGADGVAGPK.G	Oxidation:3,12
856	6	544.7739	3.14	2	32.6	75.4	0	496-507	R.GFPGADGVAGPK.G	Oxidation:3
327	4	552.6063	3.55	3	22.7	61.9	0	523-540	K.GSPGEAGRPGEAGLPGAK.G	Oxidation:3,9,15
294	2	557.9374	2.67	3	22.0	22.6	0	523-540	K.GSPGEAGRPGEAGLPGAK.G	Oxidation:3,9,15,18
248	6	629.8007	2.51	2	21.0	89.2	0	541-554	K.GLTGSPGSPGPDGK.T	Oxidation:6,9
447	4	686.3333	2.85	3	24.9	71.6	0	555-576	K.TGPPGPAGQDGRPGPPGPPGAR.G	Oxidation:3,16,18,19
868	4	597.2860	2.26	2	32.8	75.8	0	577-588	R.GQAGVMGFPGPK.G	Oxidation:6,9,12
1325	2	589.2882	1.72	2	41.6	36.2	0	577-588	R.GQAGVMGFPGPK.G	Oxidation:11,12
871	3	589.2886	2.39	2	32.8	51.2	0	577-588	R.GQAGVMGFPGPK.G	Oxidation:6,12
670	2	588.8237	2.55	2	29.1	37.5	0	601-614	R.GVPGPPGAVGPAGK.D	Oxidation:3
672	2	588.8241	3.28	2	29.2	55.8	0	601-614	R.GVPGPPGAVGPAGK.D	Oxidation:5
562	6	596.8212	2.63	2	26.9	55.8	0	601-614	R.GVPGPPGAVGPAGK.D	Oxidation:3,5
543	4	853.8916	1.79	2	26.6	97.5	0	615-633	K.DGEAGAQGPPGPAGPAGER.G	Oxidation:10
649	2	845.8942	1.87	2	28.8	111.1	0	615-633	K.DGEAGAQGPPGPAGPAGER.G	

Detailed Protein Report

Cmpd.	No. ofCmpds.	m/zmeas.	Δ m/z[ppm]	z	Rt[min]	Score	P	Range	Sequence	Modification
1370	1	1032.9872	3.35	4	42.6	36.0	0	634-678	R.GEQGPAGSPGFQGLPGPAGPPGEAGKPGE QGVPGDLGAPGPSGAR.G	Oxidation:9,15,17,20,21,26, 27
427	4	553.2924	2.31	2	24.6	33.6	0	688-699	R.GVQGPPGPAGPR.G	Oxidation:5
422	4	553.2923	2.28	2	24.5	50.6	0	688-699	R.GVQGPPGPAGPR.G	Oxidation:6
567	2	815.1074	2.57	4	27.0	46.3	1	700-735	R.GANGAPGNDGAKGDAGAPGAPGSQGAPGL QGMPGER.G	Oxidation:6,12,18,21,27,32, 33;Deamidated:3
576	2	738.9965	3.07	3	27.1	74.5	0	712-735	K.GDAGAPGAPGSQGAPGLQGMPGER.G	Oxidation:6,9,15,20,21
970	1	733.6654	3.90	3	34.7	51.1	0	712-735	K.GDAGAPGAPGSQGAPGLQGMPGER.G	Oxidation:6,9,15,21
974	2	1099.9919	1.59	2	34.8	35.5	0	712-735	K.GDAGAPGAPGSQGAPGLQGMPGER.G	Oxidation:6,9,20,21
277	4	392.2222	1.51	2	21.6	29.6	0	736-744	R.GAAGLPGPK.G	Oxidation:9
273	2	400.2199	2.05	2	21.5	41.2	0	736-744	R.GAAGLPGPK.G	Oxidation:6,9
194	2	376.5323	1.79	3	19.7	66.2	1	736-747	R.GAAGLPGPKGDR.G	Oxidation:6,9
197	1	376.5331	3.87	3	19.8	31.7	1	736-747	R.GAAGLPGPKGDR.G	Oxidation:8,9
1135	2	795.9111	1.70	2	37.9	75.6	0	766-783	R.GLTGPIGPPGPAGAPGDK.G	Oxidation:9,15
1232	2	721.6141	3.92	4	39.8	142.1	1	766-798	R.GLTGPIGPPGPAGAPGDKGETGPSGPAGPT GAR.G	Oxidation:11,15
1228	1	721.6136	3.28	4	39.7	88.2	1	766-798	R.GLTGPIGPPGPAGAPGDKGETGPSGPAGPT GAR.G	Oxidation:15,18
1214	2	725.6119	2.60	4	39.5	46.4	1	766-798	R.GLTGPIGPPGPAGAPGDKGETGPSGPAGPT GAR.G	Oxidation:9,11,15
372	2	656.3195	2.20	2	23.5	92.6	0	784-798	K.GETGPSGPAGPTGAR.G	
1147	1	712.0017	3.24	3	38.1	34.4	0	805-828	R.GEPGPPGPAGFAGPPGADGQPGAK.G	Oxidation:3,5,24
1137	1	717.3386	10.60	3	37.9	22.0	0	805-828	R.GEPGPPGPAGFAGPPGADGQPGAK.G	Oxidation:3,5,6,24
1084	2	717.3350	5.53	3	36.8	38.9	0	805-828	R.GEPGPPGPAGFAGPPGADGQPGAK.G	Oxidation:3,5,21,24
1098	2	785.8778	4.02	4	37.1	34.5	1	829-864	K.GEPGDAGAKGDAGPPGPAGPTGPPGPIGSV GAPGPK.G	Oxidation:3,9,14,15,35,36
1208	2	776.0509	3.02	3	39.4	48.1	0	838-864	K.GDAGPPGPAGPTGPPGPIGSVGAPGPK.G	Oxidation:5,24,26,27

DetailedProteinReport

Cmpd.	No. ofCmpds .	m/zmeas.	Δ m/z[ppm]	z	Rt[min]	Score	P	Range	Sequence	Modification
1240	2	770.7190	2.74	3	39.9	50.5	0	838-864	K.GDAGPPGPAGPTGPPGPIGSVGAPGPK.G	Oxidation:15,17,24
863	4	730.3514	2.08	2	32.8	98.5	0	868-884	R.GSAGPPGATGFPGAAGR.V	Oxidation:6,12
657	1	604.9691	2.86	3	28.9	27.2	0	885-905	R.VGPPGPSGNAGPPGPPGPAGK.E	Oxidation:3,18,21
558	2	914.9465	1.86	2	26.8	48.6	0	885-905	R.VGPPGPSGNAGPPGPPGPAGK.E	Oxidation:3,4,18,21
458	2	730.0159	3.07	3	25.1	40.8	0	913-936	R.GETGPAGRPGEAGPPGPPGPAGEK.G	Oxidation:14,15,17
433	2	735.3474	2.77	3	24.6	34.5	0	913-936	R.GETGPAGRPGEAGPPGPPGPAGEK.G	Oxidation:14,15,17,18
429	2	735.3478	3.32	3	24.6	29.3	0	913-936	R.GETGPAGRPGEAGPPGPPGPAGEK.G	Oxidation:5,18,20,24
456	2	730.0161	3.26	3	25.0	36.4	0	913-936	R.GETGPAGRPGEAGPPGPPGPAGEK.G	Oxidation:15,17,18
899	2	1077.2580	2.57	4	33.4	40.1	1	913-960	R.GETGPAGRPGEAGPPGPPGPAGEKGSPGADGP AGAPGTPGPQGIAGQR .G	Oxidation:18,20,24,27,32,36, 39
963	2	1073.2604	3.60	4	34.6	43.4	1	913-960	R.GETGPAGRPGEAGPPGPPGPAGEKGSPGADGP AGAPGTPGPQGIAGQR .G	Oxidation:18,20,24,27,32,36
989	1	697.0089	3.06	3	35.0	51.0	0	937-960	K.GSPGADGPAGAPGTPGPQGIAGQR.G	Oxidation:12
921	2	702.3408	3.44	3	33.8	77.4	0	937-960	K.GSPGADGPAGAPGTPGPQGIAGQR.G	Oxidation:3,12
925	2	702.3407	3.33	3	33.9	68.1	0	937-960	K.GSPGADGPAGAPGTPGPQGIAGQR.G	Oxidation:8,12
806	2	1061.0032	1.71	2	31.8	59.5	0	937-960	K.GSPGADGPAGAPGTPGPQGIAGQR.G	Oxidation:3,12,17
807	2	707.6723	3.21	3	31.8	98.4	0	937-960	K.GSPGADGPAGAPGTPGPQGIAGQR.G	Oxidation:3,12,15
804	2	707.6727	3.71	3	31.8	84.0	0	937-960	K.GSPGADGPAGAPGTPGPQGIAGQR.G	Oxidation:8,12,15
715	3	449.7596	1.59	2	30.0	40.7	0	961-969	R.GVVGLPGQR.G	Oxidation:6
1363	3	664.8291	2.07	2	42.4	75.0	0	973-986	R.GFPGLPGPSGEPGK.Q	Oxidation:3,6
1421	1	664.8291	2.14	2	43.6	21.9	0	973-986	R.GFPGLPGPSGEPGK.Q	Oxidation:3,14
1327	6	672.8265	1.95	2	41.7	63.8	0	973-986	R.GFPGLPGPSGEPGK.Q	Oxidation:3,6,14
1322	1	672.8267	2.35	2	41.6	42.0	0	973-986	R.GFPGLPGPSGEPGK.Q	Oxidation:3,6,8

Detailed Protein Report

Cmpd.	No. ofCmpds.	m/zmeas.	Δ m/z[ppm]	z	Rt[min]	Score	P	Range	Sequence	Modification
1070	3	611.6267	3.25	3	36.6	78.5	0	997-1016	R.GPPGPMGPPGLAGPPGESGR.E	Oxidation:2,6,15
1169	2	916.9349	1.65	2	38.6	54.2	0	997-1016	R.GPPGPMGPPGLAGPPGESGR.E	Oxidation:6,9,14
942	4	616.9584	3.37	3	34.2	61.8	0	997-1016	R.GPPGPMGPPGLAGPPGESGR.E	Oxidation:2,3,6,15
980	2	924.9324	1.62	2	34.9	54.0	0	997-1016	R.GPPGPMGPPGLAGPPGESGR.E	Oxidation:6,8,9,14
730	2	828.4042	2.86	3	30.3	57.1	1	1036-1064	K.GDRGESGPAGPPGAPGAPGAPGPVGPAGK.S	Oxidation:12,15,18
725	2	828.4045	3.25	3	30.2	26.6	1	1036-1064	K.GDRGESGPAGPPGAPGAPGAPGPVGPAGK.S	Oxidation:15,18,21
632	2	833.7363	3.41	3	28.4	111.8	1	1036-1064	K.GDRGESGPAGPPGAPGAPGAPGPVGPAGK.S	Oxidation:12,15,18,21
737	2	724.3521	2.50	3	30.4	69.8	0	1039-1064	R.GESGPAGPPGAPGAPGAPGPVGPAGK.S	Oxidation:9,12,15,18
857	2	654.6665	3.14	3	32.7	87.8	1	1065-1086	K.SGDRGETGPAGPAGPVGPVGAR.G	
1012	4	773.9033	1.41	2	35.4	112.3	0	1069-1086	R.GETGPAGPAGPVGPVGAR.G	
105	2	418.7225	0.40	2	17.4	30.1	0	1087-1095	R.GPAGPQGPR.G	
1289	2	907.7572	2.58	3	40.9	109.5	0	1114-1143	R.GFSGLQGPPGPPGSPGEQGPSGASGPAGPR.G	Oxidation:9,11,12,15
1435	2	897.0942	2.93	3	43.9	113.6	0	1114-1143	R.GFSGLQGPPGPPGSPGEQGPSGASGPAGPR.G	Oxidation:12,15
1438	2	897.0942	2.92	3	43.9	104.9	0	1114-1143	R.GFSGLQGPPGPPGSPGEQGPSGASGPAGPR.G	Oxidation:9,11
1392	4	902.4258	2.92	3	43.1	139.9	0	1114-1143	R.GFSGLQGPPGPPGSPGEQGPSGASGPAGPR.G	Oxidation:11,12,15
1443	2	781.4030	1.15	2	44.0	62.4	0	1155-1170	K.DGLNGLPGPIGPPGPR.G	Oxidation:7,13,15
1468	2	521.2717	2.39	3	44.5	62.9	0	1155-1170	K.DGLNGLPGPIGPPGPR.G	Oxidation:7,12,15
1477	2	781.8963	2.76	2	44.7	44.3	0	1155-1170	K.DGLNGLPGPIGPPGPR.G	Oxidation:9,12,13; Deamidated:4
1482	4	781.8957	2.03	2	44.8	77.9	0	1155-1170	K.DGLNGLPGPIGPPGPR.G	Oxidation:7,13,15; Deamidated:4

Detailed Protein Report

Protein2: Collagenalpha-1(III)chainprecursor[Susscrofa]

**Accession:**	NP_001230226.1	**Score:**	1677.83
**Database:**	NCBI_Collagen_2020	**MW[kDa]:**	138.50
**Seq.Coverage[%]:**	24.50%	**pI:**	6.33
		**No.ofPeptides:**	33

Modification(s): Oxidation,Deamidated

10	20	30	40	50	60	70	80	90	100	110	120
MTSFVQKGTW	LLFALLHPTV	ILAQQQEAIE	GGCSHLGQSY	ADRDVWKPEP	CQICVCDSGS	VLCDDIICDD	QELDCPNPEI	PFGECCAVCP	QPPTAPTRPP	NGHGPQGPKG	DPGPPGIPGR
130	140	150	160	170	180	190	200	210	220	230	240
NGDPGLPGQP	GSPGSPGPPG	ICESCPTGGQ	NYSPQYESYD	VKAGVAGGGI	GGYPGPAGPP	GPPGPPGVSG	HPGAPGSPGY	QGPPGEPGQA	GPAGPPGPPG	AIGPSGPAGK	DGESGRPGRP
250	260	270	280	290	300	310	320	330	340	350	360
GERGLPGPPG	LKGPAGMPGF	PGMKGHRGFD	GRNGEKGDTG	APGLKGENGL	PGENGAPGPM	GPRGAPGERG	RPGLPGAAGA	RGNDGARGSD	GQPGPPGPPG	TAGFPGSPGA	KGEVGPAGSP
370	380	390	400	410	420	430	440	450	460	470	480
GPSGSPGQRG	EPGPQGHAGA	AGPPGPPGSN	GSPGGKGEMG	PAGIPGAPGL	MGARGPPGPP	GTNGAPGQRG	AAGEPGKNGA	KGEPGPRGER	GEAGSPGIPG	PKGEDGKDGS	PGEPGANGLP
490	500	510	520	530	540	550	560	570	580	590	600
GAAGERGMPG	FRGAPGANGL	PGEKGPAGER	GGPGPAGPRG	VAGEPGRDGV	PGGPGLRGMP	GSPGGPGSDG	KPGPPGSQGE	SGRPGPPGSP	GPRGQPGVMG	FPGPKGNDGA	PGKNGERGGP
610	620	630	640	650	660	670	680	690	700	710	720
GGPGLPGPPG	KNGETGPQGP	PGPTGPGGDK	GDTGPPGQQG	LQGLPGTSGP	PGENGKPGEP	GPKGEAGAPG	IPGGKGDSGA	PGERGPPGAV	GPSGPRGGAG	PPGPEGGKGP	AGPPGPPGAA
730	740	750	760	770	780	790	800	810	820	830	840
GTPGLQGMPG	ERGGSGGPGP	KGDKGDPGGS	GADGAPGKDG	PRGPTGPIGP	PGPAGQPGDK	GESGAPGLPG	IAGPRGGPGE	RGEHGPPGPA	GFPGAPGQNG	EPGAKGERGA	PGEKGEGGPP
850	860	870	880	890	900	910	920	930	940	950	960
GIAGQPGGTG	PPGPPGPQGV	KGERGSPGGP	GAAGFPGGRG	LPGPPGSNGN	PGPPGSSGPP	GKDGPPGPPG	SSGAPGSPGV	SGPKGDAGQP	GEKGSPGPQG	PPGAPGPGGI	SGITGARGLA
970	980	990	1000	1010	1020	1030	1040	1050	1060	1070	1080
GPPGMPGARG	SPGPQGVKGE	NGKPGPSGLN	GERGPPGPQG	LPGLAGAAGE	PGRDGNPGSD	GLPGRDGAPG	SKGDRGENGS	PGAPGAPGHP	GPPGPVGPAG	KNGDRGETGP	AGPAGAPGPA
1090	1100	1110	1120	1130	1140	1150	1160	1170	1180	1190	1200
GSRGAPGPQG	PRGDKGETGE	RGANGIKGHR	GFPGNPGAPG	SPGPAGHQGA	VGSPGPAGPR	GPVGPSGPPG	KDGASGHPGP	IGPPGPRGNR	GERGSEGSPG	HPGQPGPPGP	PGAPGPCCGG
1210	1220	1230	1240	1250	1260	1270	1280	1290	1300	1310	1320
GAAAIAGVGG	EKAGGFAPYY	GDEPMDFKIN	TDEIMTSLKS	VNGQIESLIS	PDGSRKNPAR	NCRDLKFCHP	ELKSGEYWVD	PNQGCKMDAI	KVFCNMETGE	TCISASPSTV	PRKNWWTDSG
1330	1340	1350	1360	1370	1380	1390	1400	1410	1420	1430	1440
AEKKYVWFGE	SMNGGFQFSY	GNPELPEDVL	DVQLAFLRLL	SSRASQNITY	HCKNSIAYME	HASGNVKKAL	RLMGSNEGEF	KAEGNSKFTY	TVLEDGCTKH	TGEWGKTVFE	YRTRKAVRLP
1450	1460	1470									
IVDIAPYDIG	GPDQEFGADI	GPVCFL									

Detailed Protein Report

Cmpd.	No. ofCmpds.	m/zmeas.	Δ m/z[ppm]	z	Rt[min]	Score	P	Range	Sequence	Modification
246	2	371.2049	3.02	3	20.9	45.6	0	310-321	R.GRPGLPGAAGAR.G	Oxidation:3,6
320	1	813.3804	1.31	2	22.6	64.0	0	352-369	K.GEVGPAGSPGPSGSPGQR.G	Oxidation:9,15
198	3	704.8290	4.03	2	19.8	88.7	0	415-429	R.GPPGPPGTNGAPGQR.G	Oxidation:2,6,12;Deamidated: 9
193	1	704.8267	0.62	2	19.7	32.0	0	415-429	R.GPPGPPGTNGAPGQR.G	Oxidation:2,6,12;Deamidated: 14
371	2	549.7763	2.46	2	23.5	33.8	0	451-462	R.GEAGSPGIPGPK.G	Oxidation:6,12
297	2	534.2551	3.60	3	22.0	36.1	1	451-467	R.GEAGSPGIPGPKGEDGK.D	Oxidation:6,9,12
299	2	534.2546	2.63	3	22.1	23.4	1	451-467	R.GEAGSPGIPGPKGEDGK.D	Oxidation:6,9,17
660	2	878.8920	1.97	2	28.9	32.3	0	468-486	K.DGSPGEPGANGLPGAAGER.G	Oxidation:4,7,13
472	2	478.7445	2.16	2	25.4	30.8	0	528-537	R.DGVPGGPGLR.G	Oxidation:4,7
885	1	610.2948	3.81	2	33.1	44.3	0	574-585	R.GQPGVMGFPGPK.G	Oxidation:3,6,9
841	1	618.2911	1.90	2	32.4	37.6	0	574-585	R.GQPGVMGFPGPK.G	Oxidation:3,6,9,12
388	2	604.8007	2.86	2	23.8	77.8	0	598-611	R.GGPGGPGLPGPPGK.N	Oxidation:3,6,9,14
302	2	521.7620	0.35	2	22.1	70.1	0	664-675	K.GEAGAPGIPGGK.G	Oxidation:6,9
303	2	521.7628	1.80	2	22.2	58.4	0	664-675	K.GEAGAPGIPGGK.G	Oxidation:6,12
333	2	532.7790	2.30	2	22.8	56.2	0	685-696	R.GPPGAVGPSGPR.G	Oxidation:3
834	2	736.0131	2.39	3	32.3	41.1	0	709-732	K.GPAGPPGPPGAAGTPGLQGMPGER.G	Oxidation:8,9,15,20,21
831	2	736.0131	2.51	3	32.2	36.1	0	709-732	K.GPAGPPGPPGAAGTPGLQGMPGER.G	Oxidation:2,5,15,20,21
1194	1	684.3519	3.77	2	39.0	22.9	0	781-795	K.GESGAPGLPGIAGPR.G	Oxidation:6,9
801	1	750.0045	3.94	3	31.7	37.4	0	802-825	R.GEHGPPGPAGFPGAPGQNGEPGAK.G	Oxidation:8,12,15,21; Deamidated:18
785	1	749.6756	2.78	3	31.4	43.7	0	802-825	R.GEHGPPGPAGFPGAPGQNGEPGAK.G	Oxidation:8,12,15,21
467	2	645.2986	2.32	2	25.3	86.2	0	865-879	R.GSPGGPGAAGFPGGR.G	Oxidation:3,6,12
1328	2	697.3486	2.43	3	41.7	28.9	0	934-957	K.GSPGPQGPPGAPGPGGISGITGAR.G	Oxidation:3,12,14
1323	2	697.3481	1.74	3	41.6	42.5	0	934-957	K.GSPGPQGPPGAPGPGGISGITGAR.G	Oxidation:5,8,9
322	2	564.7785	2.68	2	22.6	53.4	0	958-969	R.GLAGPPGMPGAR.G	Oxidation:6,8,9
284	2	496.2324	3.90	3	21.8	83.5	0	979-993	K.GENGKPGPSGLNGER.G	Oxidation:6;Deamidated:3,12
1416	2	601.9725	2.56	3	43.5	58.6	0	994-1013	R.GPPGPQGLPGLAGAAGEPGR.D	Oxidation:5,9,18
1424	2	601.9728	3.01	3	43.7	30.4	0	994-1013	R.GPPGPQGLPGLAGAAGEPGR.D	Oxidation:2,3,18
292	2	587.2608	1.11	2	21.9	51.3	0	1014-1025	R.DGNPGSDGLPGR.D	Oxidation:4,10

[illegible]

Detailed Protein Report

Cmpd.	No. ofCmpds.	m/zmeas.	Δ m/z[ppm]	z	Rt[min]	Score	P	Range	Sequence	Modification
770	2	672.0673	2.76	4	31.0	30.3	0	1111-1140	R.GFPGNPGAPGSPGPAGHQGAVGSPGPAGPR.G	Oxidation:6,9,12,14,24
768	3	895.7537	2.44	3	31.0	70.0	0	1111-1140	R.GFPGNPGAPGSPGPAGHQGAVGSPGPAGPR.G	Oxidation:3,6,9,12,29
361	1	475.2592	0.99	2	23.3	33.2	0	1141-1151	R.GPVGPSGPPGK.D	
210	2	483.2571	1.92	2	20.1	41.4	0	1141-1151	R.GPVGPSGPPGK.D	Oxidation:11
465	2	500.9126	2.91	3	25.2	22.6	0	1152-1167	K.DGASGHPGPIGPPGPR.G	Oxidation:9,13

Detailed Protein Report

Project Info

**Name:**	Spongin	**Date:**	February 26, 2018
**Sample Info &Protocols**		**Date:**	September 24, 2021
**Name:**	210723C		

SearchResult Info

**SearchResult**	**Location**	**Search Engine**	**Database**	**Ident. Compounds**
NCBI-collagen-2020_2021-09-25 16:55:39	/Spongin/Spongin C/210723 C_C6_01_5082.d	Mascot, 2.3.0	NCBI_Collagen_2020, NCBI_Collagen_D20200704_collagenonly_final.fasta	184/3630

7

**Protein 1:**	collagen alpha-2(I) chain precursor [Sus scrofa]		
**Accession:**	NP_001230584.1	**Score:**	4409.82
**Database:**	NCBI_Collagen_2020	**MW [kDa]:**	129.10
**Seq. Coverage [%]:**	50.40 %	**pI:**	9.79
		**No. of Peptides:**	79
**Modification(s):**	Oxidation, Deamidated		

Detailed Protein Report

10	20	30	40	50	60	70	80	90	100	110	120
MLSFVDTRTL	LLLAVTSCLA	TCQSLQEATA	RKGPTGDRGP	RGERGPPGPP	GRDGDDGIPG	PPGPPGPPGP	PGLGGNFAAQ	YDGKGVGAGP	GPMGLMGPRG	PPGAVGAPGP	QGFQGPAGEP

130	140	150	160	170	180	190	200	210	220	230	240
GEPGQTGPAG	ARGPPGPPGK	AGEDGHPGKP	GRPGERGVVG	PQGARGFPGT	PGLPGFKGIR	GHNGLDGLKG	QPGAPGVKGE	PGAPGENGTP	GQTGARGLPG	ERGRVGAPGP	AGARGNDGSV

250	260	270	280	290	300	310	320	330	340	350	360
GPVDPAGPIG	SAGPPGFPGA	PGPKGELGPV	GNPGPAGPAG	PRGEVGLPGV	SGPVGPPGNP	GANGLPGAKG	AAGLPGVAGA	PGLPGPRGIP	GPAGAAGATG	ARGLVGEPGP	AGSKGESGNK

370	380	390	400	410	420	430	440	450	460	470	480
GEPGAAGPQG	PPGPSGEEGK	RGPNGEVGSA	GPPGPPGLRG	NPGSRGLPGA	DGRAGVMGPP	GSRGPTGPAG	VRGPNGDSGR	PGEPGLMGPR	GFPGSPGNVG	PAGKEGPAGL	PGIDGRPGPI

490	500	510	520	530	540	550	560	570	580	590	600
GPAGARGEPG	NIGFPGPKGP	TGDPGKNGEK	GHAGLAGARG	APGPDGNNGA	QGPPGPQGVQ	GGKGEQGPAG	PPGFQGLPGP	AGTAGEVGKP	GERGIPGEFG	LPGPAGPRGE	RGPPGESGAA

610	620	630	640	650	660	670	680	690	700	710	720
GPAGPIGSRG	PSGPPGPDGN	KGEPGVLGAP	GTAGPSGPSG	LPGERGAAGI	PGGKGEKGET	GLRGDVGSPG	RDGARGAPGA	VGAPGPAGAN	GDRGEAGPAG	PAGPAGPRGS	PGERGEVGPA

730	740	750	760	770	780	790	800	810	820	830	840
GPNGFAGPAG	AAGQPGAKGE	RGTKGPKGEN	GPVGPTGPVG	AAGPAGPNGP	PGPAGSRGDG	GPPGATGFPG	AAGRIGPPGP	SGISGPPGPP	GPAGKEGLRG	PRGDQGPVGR	TGETGASGPP

850	860	870	880	890	900	910	920	930	940	950	960
GFAGEKGPSG	EPGTAGPPGT	PGPQGILGAP	GFLGLPGSRG	ERGLPGVAGS	VGEPGPLGIA	GPPGARGPPG	AVGNPGVNGA	PGEAGRDGNP	GSDGPPGRDG	QAGHKGERGY	PGNPGPAGAA

970	980	990	1000	1010	1020	1030	1040	1050	1060	1070	1080
GAPGPQGAVG	PAGKHGNRGE	PGPAGSVGPA	GAVGPRGPSG	PQGIRGEKGE	PGDKGPRGLP	GLKGHNGLQG	LPGLAGHHGD	QGAPGPVGPA	GPRGPAGPSG	PAGKDGRTGQ	PGAVGPAGIR

1090	1100	1110	1120	1130	1140	1150	1160	1170	1180	1190	1200
GSQGSQGPAG	PPGPPGPPGP	PGPSGGGYDF	GYEGDFYRAD	QPRSPPSLRP	KDYEVDATLK	SLNNQIETLL	TPEGSRKNPA	RTCRDLRLSH	PEWSSGYYWI	DPNQGCTMDA	IKVYCDFSTG

1210	1220	1230	1240	1250	1260	1270	1280	1290	1300	1310	1320
ETCIRAQPEN	IPAKNWYRNS	KVKKHVWLGE	TINGGTQFEY	NMEGVTTKEM	ATQLAFMRLL	ANHASQNITY	HCKNSIAYMD	EETGNLKKAV	ILQGSNDVEL	VAEGNSRFTY	TVLVDGCSKK

1330	1340	1350	1360	1370
TNEWRKTIIE	YKTNKPSRLP	ILDIAPLDIG	DADQEVSVDV	GPVCFK

Detailed Protein Report

Cmpd.	No. of Cmpds.	m/z meas.	Δ m/z [ppm]	z	Rt [min]	Score	P	Range	Sequence	Modification
1168	2	996.4699	2.41	3	39.7	29.2	0	100-132	R.GPPGAVGAPGPQGFQGPAGEPGEPGQTG PAGAR.G	Oxidation: 2, 3, 24, 29
1166	2	996.4692	1.79	3	39.6	32.7	0	100-132	R.GPPGAVGAPGPQGFQGPAGEPGEPGQTG PAGAR.G	Oxidation: 11, 17, 21, 24
1175	1	747.6056	4.22	4	39.8	22.0	0	100-132	R.GPPGAVGAPGPQGFQGPAGEPGEPGQTG PAGAR.G	Oxidation: 9, 11, 17, 21
241	2	420.7381	0.38	2	20.6	53.4	0	157-165	R.GVVGPQGAR.G	
1594	2	619.8071	1.48	2	48.5	50.0	0	166-177	R.GFPGTPGLPGFK.G	Oxidation: 3, 6, 9, 12
164	2	850.8806	4.04	2	18.6	41.8	0	199-216	K.GEPGAPGENGTPGQTGAR.G	Oxidation: 3, 6, 12
167	2	567.9183	5.39	3	18.7	60.0	0	199-216	K.GEPGAPGENGTPGQTGAR.G	Oxidation: 3, 6, 12; Deamidated: 9
1067	2	808.4138	1.05	2	37.6	123.7	0	265-282	K.GELGPVGNPGPAGPAGPR.G	Oxidation: 9
1065	1	808.4139	1.08	2	37.5	40.0	0	265-282	K.GELGPVGNPGPAGPAGPR.G	Oxidation: 17
1530	2	811.4079	1.67	3	47.2	39.0	0	283-309	R.GEVGLPGVSGPVGPPGNPGANGLPGAK.G	Oxidation: 6, 15, 18, 24, 27
1355	2	521.6151	1.40	3	43.6	51.9	0	310-327	K.GAAGLPGVAGAPGLPGPR.G	Oxidation: 6, 12, 17
1359	4	781.9189	1.22	2	43.7	39.6	0	310-327	K.GAAGLPGVAGAPGLPGPR.G	Oxidation: 6, 15, 17
472	2	550.7840	2.28	2	25.5	38.0	0	343-354	R.GLVGEPGPAGSK.G	Oxidation: 6, 12
510	2	542.7864	2.12	2	26.1	61.6	0	343-354	R.GLVGEPGPAGSK.G	Oxidation: 6
333	1	558.2773	2.24	3	22.7	70.6	1	343-360	R.GLVGEPGPAGSKGESGNK.G	Oxidation: 6, 12
662	1	866.1592	2.85	4	29.4	25.0	2	343-380	R.GLVGEPGPAGSKGESGNKGEPGAAGPQG PPGPSGEEGK.R	Oxidation: 6, 8, 12, 38
336	1	794.0299	4.32	3	22.7	24.7	1	355-380	K.GESGNKGEPGAAGPQGPPGPSGEEGK.R	Oxidation: 6, 26
321	1	799.3614	4.20	3	22.4	39.6	1	355-380	K.GESGNKGEPGAAGPQGPPGPSGEEGK.R	Oxidation: 6, 9, 26
436	2	904.4096	1.45	2	24.7	97.0	0	361-380	K.GEPGAAGPQGPPGPSGEEGK.R	Oxidation: 3, 12
437	3	603.2766	3.26	3	24.8	39.5	0	361-380	K.GEPGAAGPQGPPGPSGEEGK.R	Oxidation: 8, 12
439	2	904.4099	1.80	2	24.8	70.3	0	361-380	K.GEPGAAGPQGPPGPSGEEGK.R	Oxidation: 3, 14
788	2	601.9767	3.30	3	32.0	59.2	1	381-399	K.RGPNGEVGSAGPPGPPGLR.G	Oxidation: 13, 16
828	2	602.3043	2.66	3	32.8	56.8	1	381-399	K.RGPNGEVGSAGPPGPPGLR.G	Oxidation: 13, 15; Deamidated: 4
1006	2	824.9009	1.21	2	36.2	42.5	0	382-399	R.GPNGEVGSAGPPGPPGLR.G	Oxidation: 14, 15; Deamidated: 3
235	3	406.2259	3.01	2	20.4	44.8	0	424-432	R.GPTGPAGVR.G	
485	2	600.6061	3.34	3	25.7	30.6	0	433-450	R.GPNGDSGRPGEPGLMGPR.G	Oxidation: 9, 12, 17; Deamidated: 3
487	2	600.6059	2.98	3	25.8	50.0	0	433-450	R.GPNGDSGRPGEPGLMGPR.G	Oxidation: 9, 12, 15; Deamidated: 3
655	3	637.3139	2.62	2	29.2	75.5	0	451-464	R.GFPGSPGNVGPAGK.E	Oxidation: 3, 6

Detailed Protein Report

Cmpd.	No. of Cmpds.	m/z meas.	Δ m/z [ppm]	z	Rt [min]	Score	P	Range	Sequence	Modification
1047	4	601.2973	2.06	2	37.2	68.6	0	487-498	R.GEPGNIGFPGPK.G	Oxidation: 3, 11
527	2	716.0047	4.12	3	26.5	57.4	0	520-543	R.GAPGPDGNNGAQGPPGPQGVQGGK.G	Oxidation: 3, 15
575	1	716.3326	3.87	3	27.4	36.3	0	520-543	R.GAPGPDGNNGAQGPPGPQGVQGGK.G	Oxidation: 5, 14; Deamidated: 8
1386	2	940.7919	2.29	3	44.2	105.8	0	544-573	K.GEQGPAGPPGFQGLPGPAGTAGEVGKPGER.G	Oxidation: 9, 15, 17
1394	2	705.8455	1.99	4	44.4	31.0	0	544-573	K.GEQGPAGPPGFQGLPGPAGTAGEVGKPGER.G	Oxidation: 5, 8, 27
1588	2	727.3764	1.44	2	48.4	46.7	0	574-588	R.GIPGEFGLPGPAGPR.G	Oxidation: 3, 14
1596	2	485.2545	3.72	3	48.5	99.2	0	574-588	R.GIPGEFGLPGPAGPR.G	Oxidation: 3, 9
1468	2	735.3741	1.79	2	45.9	60.2	0	574-588	R.GIPGEFGLPGPAGPR.G	Oxidation: 3, 9, 11
1465	2	735.3728	0.04	2	45.8	52.8	0	574-588	R.GIPGEFGLPGPAGPR.G	Oxidation: 3, 9, 14
723	2	775.8828	1.69	2	30.7	136.4	0	592-609	R.GPPGESGAAGPAGPIGSR.G	Oxidation: 3
594	2	783.8808	2.32	2	27.8	135.7	0	592-609	R.GPPGESGAAGPAGPIGSR.G	Oxidation: 3, 11
1210	2	815.1408	3.28	4	40.5	51.7	1	610-645	R.GPSGPPGPDGNKGEPGVLGAPGTAGPSGPSGLPGER.G	Oxidation: 8, 12, 15, 21, 26
1207	2	815.1401	2.53	4	40.4	43.6	1	610-645	R.GPSGPPGPDGNKGEPGVLGAPGTAGPSGPSGLPGER.G	Oxidation: 2, 5, 6, 8, 33
1417	2	717.0238	2.21	3	44.8	104.0	0	622-645	K.GEPGVLGAPGTAGPSGPSGLPGER.G	Oxidation: 3, 9
1284	2	722.3557	2.60	3	42.0	104.3	0	622-645	K.GEPGVLGAPGTAGPSGPSGLPGER.G	Oxidation: 3, 9, 21
1290	1	1083.0289	1.71	2	42.1	26.6	0	622-645	K.GEPGVLGAPGTAGPSGPSGLPGER.G	Oxidation: 3, 14, 17
384	2	508.9078	3.41	3	23.7	20.6	0	676-693	R.GAPGAVGAPGPAGANGDR.G	Oxidation: 9, 11; Deamidated: 15
388	3	762.8568	1.70	2	23.8	82.1	0	676-693	R.GAPGAVGAPGPAGANGDR.G	Oxidation: 3, 9; Deamidated: 15
398	3	762.8568	1.72	2	24.0	56.4	0	676-693	R.GAPGAVGAPGPAGANGDR.G	Oxidation: 3, 11; Deamidated: 15
354	2	762.3645	1.36	2	23.1	58.8	0	676-693	R.GAPGAVGAPGPAGANGDR.G	Oxidation: 3, 9
350	2	762.3646	1.51	2	23.0	38.5	0	676-693	R.GAPGAVGAPGPAGANGDR.G	Oxidation: 3, 11
839	2	692.5870	4.29	4	33.0	89.2	1	676-708	R.GAPGAVGAPGPAGANGDRGEAGPAGPAGPAGPR.G	Oxidation: 3, 9; Deamidated: 15
829	2	923.1113	1.89	3	32.9	38.6	1	676-708	R.GAPGAVGAPGPAGANGDRGEAGPAGPAGPAGPR.G	Oxidation: 3, 9; Deamidated: 33
517	2	631.3190	1.83	2	26.3	96.5	0	694-708	R.GEAGPAGPAGPAGPR.G	
1170	1	1034.4947	4.39	2	39.7	88.0	0	715-738	R.GEVGPAGPNGFAGPAGAAGQPGAK.G	Oxidation: 21, 24; Deamidated: 9
1167	1	1033.9991	0.93	2	39.7	80.7	0	715-738	R.GEVGPAGPNGFAGPAGAAGQPGAK.G	Oxidation: 21, 24

Detailed Protein Report

Cmpd.	No. of Cmpds.	m/z meas.	Δ m/z [ppm]	z	Rt [min]	Score	P	Range	Sequence	Modification
1049	2	804.0541	3.95	3	37.2	126.4	1	715-741	R.GEVGPAGPNGFAGPAGAAGQPGAKGER.G	Oxidation: 21, 24; Deamidated: 9
1063	2	955.4778	9.30	3	37.5	70.4	1	745-777	K.GPKGENGPVGPTGPVGAAGPAGPNGPPGPAGSR.G	Oxidation: 2, 29; Deamidated: 6
1070	1	955.4753	6.70	3	37.6	44.1	1	745-777	K.GPKGENGPVGPTGPVGAAGPAGPNGPPGPAGSR.G	Oxidation: 2, 29; Deamidated: 24
1197	2	856.0843	4.05	3	40.2	90.0	0	748-777	K.GENGPVGPTGPVGAAGPAGPNGPPGPAGSR.G	Oxidation: 24; Deamidated: 21
1199	1	856.0840	3.70	3	40.3	37.9	0	748-777	K.GENGPVGPTGPVGAAGPAGPNGPPGPAGSR.G	Oxidation: 26; Deamidated: 3
817	2	737.3407	1.61	2	32.6	58.6	0	778-794	R.GDGGPPGATGFPGAAGR.I	Oxidation: 6, 12
813	2	737.3408	1.82	2	32.6	40.2	0	778-794	R.GDGGPPGATGFPGAAGR.I	Oxidation: 5, 12
997	2	929.4726	2.05	2	36.1	41.1	0	795-815	R.IGPPGPSGISGPPGPPGPAGK.E	Oxidation: 3, 4, 18, 21
907	1	625.3165	3.22	3	34.5	24.0	0	795-815	R.IGPPGPSGISGPPGPPGPAGK.E	Oxidation: 4, 6, 12, 13, 15
735	2	739.8426	1.50	2	30.9	85.4	0	831-846	R.TGETGASGPPGFAGEK.G	Oxidation: 10
2411	2	1011.8387	3.37	3	62.7	66.4	0	847-879	K.GPSGEPGTAGPPGTPGPQGILGAPGFLGLPGSR.G	Oxidation: 2, 15, 17, 24, 30
1832	2	1066.0618	0.99	2	52.8	103.7	0	883-906	R.GLPGVAGSVGEPGPLGIAGPPGAR.G	Oxidation: 3, 12, 21
478	2	889.9251	0.74	2	25.6	74.2	0	907-926	R.GPPGAVGNPGVNGAPGEAGR.D	Oxidation: 3, 9, 15
482	2	593.6208	3.55	3	25.7	29.0	0	907-926	R.GPPGAVGNPGVNGAPGEAGR.D	Oxidation: 2, 3, 15
531	2	593.9483	2.72	3	26.6	37.8	0	907-926	R.GPPGAVGNPGVNGAPGEAGR.D	Oxidation: 3, 9, 15; Deamidated: 12
546	2	593.9489	3.59	3	26.8	24.4	0	907-926	R.GPPGAVGNPGVNGAPGEAGR.D	Oxidation: 2, 3, 15; Deamidated: 12
908	2	735.6965	3.05	3	34.5	50.2	0	949-974	R.GYPGNPGPAGAAGAPGPQGAVGPAGK.H	Oxidation: 8, 15
824	2	741.0290	4.14	3	32.8	60.3	0	949-974	R.GYPGNPGPAGAAGAPGPQGAVGPAGK.H	Oxidation: 6, 8, 15
830	2	741.0277	2.41	3	32.9	29.2	0	949-974	R.GYPGNPGPAGAAGAPGPQGAVGPAGK.H	Oxidation: 3, 6, 8
909	2	516.9323	3.69	3	34.5	97.4	0	979-996	R.GEPGPAGSVGPAGAVGPR.G	Oxidation: 3
1007	2	766.8961	2.12	2	36.3	47.2	0	979-996	R.GEPGPAGSVGPAGAVGPR.G	
343	3	434.7364	2.36	2	22.9	44.2	0	997-1005	R.GPSGPQGIR.G	
1265	2	703.5999	3.79	4	41.6	28.7	0	1024-1053	K.GHNGLQGLPGLAGHHGDQGAPGPVGPAGPR.G	Oxidation: 9, 29; Deamidated: 6
806	2	590.8261	1.36	2	32.4	45.4	0	1068-1080	R.TGQPGAVGPAGIR.G	
682	2	598.8241	2.35	2	29.9	45.2	0	1068-1080	R.TGQPGAVGPAGIR.G	Oxidation: 4

Detailed Protein Report

Protein 2: collagen alpha-1(I) chain isoform X1 [Sus scrofa]
Accession: XP_020922812.1 **Score:** 3449.69
Database: NCBI_Collagen_2020 **MW [kDa]:** 139.20
Seq. Coverage [%]: 45.70 % **pI:** 5.50
No. of Peptides: 66

Modification(s): Oxidation, Deamidated

Cmpd.	No. of Cmpds.	m/z meas.	Δ m/z [ppm]	z	Rt [min]	Score	P	Range	Sequence	Modification
860	1	581.7932	1.57	2	33.4	38.6	0	256-267	R.GLPGTAGLPGMK.G	Oxidation: 3, 9, 11, 12
890	2	426.2173	1.76	2	34.1	36.2	0	271-279	R.GFSGLDGAK.G	
193	2	880.3650	2.21	2	19.3	65.3	0	289-306	K.GEPGSPGENGAPGQMGPR.G	Oxidation: 3, 6, 12, 17; Deamidated: 9
113	3	374.5330	3.02	3	17.2	42.3	0	313-324	R.GRPGPPGPAGAR.G	Oxidation: 3, 6
1268	2	855.4078	3.01	3	41.7	83.1	0	325-354	R.GNDGATGAAGPPGPTGPAGPPGFPGAVGAK.G	Oxidation: 11, 21, 24, 30
544	3	768.0182	3.08	3	26.8	54.0	0	373-399	R.GEPGPPGPAGAAGPAGNPGADGQPGGK.G	Oxidation: 3, 5, 24, 27
547	3	768.0184	3.31	3	26.9	36.6	0	373-399	R.GEPGPPGPAGAAGPAGNPGADGQPGGK.G	Oxidation: 8, 14, 18, 24
1144	2	793.8834	2.37	2	39.2	45.8	0	400-417	K.GANGAPGIAGAPGFPGAR.G	Oxidation: 6, 12, 15; Deamidated: 3
369	2	674.8302	2.97	2	23.4	111.3	0	418-432	R.GPSGPQGPSGPPGPK.G	Oxidation: 12, 14
363	2	674.8297	2.16	2	23.3	83.2	0	418-432	R.GPSGPQGPSGPPGPK.G	Oxidation: 14, 15
379	2	666.8320	1.90	2	23.6	114.2	0	418-432	R.GPSGPQGPSGPPGPK.G	Oxidation: 12
376	2	666.8320	1.82	2	23.6	54.6	0	418-432	R.GPSGPQGPSGPPGPK.G	Oxidation: 8
595	1	799.0404	2.48	3	27.9	55.0	1	445-470	K.GDTGAKGEPGPTGVQGPPGPAGEEGK.R	Oxidation: 6, 9, 26
706	2	925.4319	0.14	2	30.3	58.9	0	451-470	K.GEPGPTGVQGPPGPAGEEGK.R	Oxidation: 3, 12
711	1	925.4327	1.06	2	30.4	29.1	0	451-470	K.GEPGPTGVQGPPGPAGEEGK.R	Oxidation: 5, 11
588	2	669.3254	2.19	3	27.7	95.6	1	451-471	K.GEPGPTGVQGPPGPAGEEGKR.G	Oxidation: 5, 12
779	4	718.3454	1.67	2	31.8	68.3	0	475-489	R.GEPGPAGLPGPPGER.G	Oxidation: 3, 9, 12
775	4	718.3459	2.34	2	31.7	45.7	0	475-489	R.GEPGPAGLPGPPGER.G	Oxidation: 5, 9, 12
816	4	718.3450	1.10	2	32.6	44.4	0	475-489	R.GEPGPAGLPGPPGER.G	Oxidation: 3, 11, 12
895	2	544.7731	1.68	2	34.2	75.2	0	496-507	R.GFPGADGVAGPK.G	Oxidation: 3
334	2	828.4057	3.54	2	22.7	42.6	0	523-540	K.GSPGEAGRPGEAGLPGAK.G	Oxidation: 3, 9, 15
258	4	629.8007	2.43	2	21.0	85.3	0	541-554	K.GLTGSPGSPGPDGK.T	Oxidation: 6, 9
262	4	629.8004	1.97	2	21.1	56.5	0	541-554	K.GLTGSPGSPGPDGK.T	Oxidation: 6, 14
440	2	686.3337	3.38	3	24.8	41.8	0	555-576	K.TGPPGPAGQDGRPGPPGPPGAR.G	Oxidation: 3, 16, 18, 19
862	2	589.2882	1.84	2	33.5	56.8	0	577-588	R.GQAGVMGFPGPK.G	Oxidation: 6, 9
859	2	589.2878	1.12	2	33.4	44.9	0	577-588	R.GQAGVMGFPGPK.G	Oxidation: 6, 12
818	2	597.2858	2.07	2	32.7	72.5	0	577-588	R.GQAGVMGFPGPK.G	Oxidation: 6, 9, 12

Detailed Protein Report

Cmpd.	No. of Cmpds.	m/z meas.	Δ m/z [ppm]	z	Rt [min]	Score	P	Range	Sequence	Modification
577	4	596.8210	2.15	2	27.5	70.4	0	601-614	R.GVPGPPGAVGPAGK.D	Oxidation: 3, 5
554	2	853.8919	2.10	2	27.0	106.4	0	615-633	K.DGEAGAQGPPGPAGPAGER.G	Oxidation: 10
550	1	853.8913	1.39	2	26.9	45.3	0	615-633	K.DGEAGAQGPPGPAGPAGER.G	Oxidation: 15
1501	1	1024.9890	2.65	4	46.6	22.6	0	634-678	R.GEQGPAGSPGFQGLPGPAGPPGEAGKPGEQGVPG DLGAPGPSGAR.G	Oxidation: 9, 15, 17, 20, 21
442	4	553.2920	1.71	2	24.9	66.2	0	688-699	R.GVQGPPGPAGPR.G	Oxidation: 6
450	1	553.2917	1.14	2	25.0	21.1	0	688-699	R.GVQGPPGPAGPR.G	Oxidation: 11
580	2	738.9959	2.36	3	27.5	71.2	0	712-735	K.GDAGAPGAPGSQGAPGLQGMPGER.G	Oxidation: 6, 9, 15, 20, 21
309	2	392.2226	2.66	2	22.1	28.8	0	736-744	R.GAAGLPGPK.G	Oxidation: 9
299	1	400.2198	1.75	2	21.9	33.4	0	736-744	R.GAAGLPGPK.G	Oxidation: 8, 9
1107	2	795.9110	1.60	2	38.4	80.5	0	766-783	R.GLTGPIGPPGPAGAPGDK.G	Oxidation: 9, 15
1189	2	961.8152	2.72	3	40.1	85.0	1	766-798	R.GLTGPIGPPGPAGAPGDKGETGPSGPAGPTGAR.G	Oxidation: 11, 15
1174	2	967.1460	1.81	3	39.8	26.0	1	766-798	R.GLTGPIGPPGPAGAPGDKGETGPSGPAGPTGAR.G	Oxidation: 9, 11, 15
1179	2	967.1453	1.13	3	39.9	52.6	1	766-798	R.GLTGPIGPPGPAGAPGDKGETGPSGPAGPTGAR.G	Oxidation: 11, 15, 18
389	2	656.3188	1.19	2	23.8	91.3	0	784-798	K.GETGPSGPAGPTGAR.G	
1181	2	776.0507	2.84	3	39.9	30.3	0	838-864	K.GDAGPPGPAGPTGPPGPIGSVGAPGPK.G	Oxidation: 5, 24, 26, 27
1176	2	776.0509	3.07	3	39.8	24.4	0	838-864	K.GDAGPPGPAGPTGPPGPIGSVGAPGPK.G	Oxidation: 5, 6, 26, 27
808	2	730.3509	1.45	2	32.5	67.6	0	868-884	R.GSAGPPGATGFPGAAGR.V	Oxidation: 6, 12
571	1	914.9464	1.65	2	27.3	28.3	0	885-905	R.VGPPGPSGNAGPPGPPGPAGK.E	Oxidation: 3, 4, 18, 21
475	1	730.0160	3.18	3	25.5	44.5	0	913-936	R.GETGPAGRPGEAGPPGPPGPAGEK.G	Oxidation: 14, 15, 17
459	2	735.3472	2.61	3	25.2	33.1	0	913-936	R.GETGPAGRPGEAGPPGPPGPAGEK.G	Oxidation: 14, 15, 17, 18
896	2	702.3405	2.92	3	34.2	57.8	0	937-960	K.GSPGADGPAGAPGTPGPQGIAGQR.G	Oxidation: 3, 12
900	2	702.3405	2.97	3	34.3	54.8	0	937-960	K.GSPGADGPAGAPGTPGPQGIAGQR.G	Oxidation: 8, 12
803	2	1061.0030	1.47	2	32.3	37.0	0	937-960	K.GSPGADGPAGAPGTPGPQGIAGQR.G	Oxidation: 3, 12, 17
794	2	707.6724	3.32	3	32.2	60.1	0	937-960	K.GSPGADGPAGAPGTPGPQGIAGQR.G	Oxidation: 3, 12, 15
798	1	707.6729	4.11	3	32.2	51.8	0	937-960	K.GSPGADGPAGAPGTPGPQGIAGQR.G	Oxidation: 8, 12, 15
733	2	449.7595	1.38	2	30.8	29.2	0	961-969	R.GVVGLPGQR.G	Oxidation: 6
1312	2	664.8276	-0.19	2	42.6	51.5	0	973-986	R.GFPGLPGPSGEPGK.Q	Oxidation: 3, 6
1255	2	672.8256	0.62	2	41.3	51.1	0	973-986	R.GFPGLPGPSGEPGK.Q	Oxidation: 3, 6, 14
925	2	616.9582	2.99	3	34.8	42.7	0	997-1016	R.GPPGPMGPPGLAGPPGESGR.E	Oxidation: 2, 3, 6, 15
928	2	616.9575	1.88	3	34.9	42.4	0	997-1016	R.GPPGPMGPPGLAGPPGESGR.E	Oxidation: 2, 3, 5, 15
929	1	924.9317	0.88	2	34.9	27.8	0	997-1016	R.GPPGPMGPPGLAGPPGESGR.E	Oxidation: 6, 8, 9, 14
635	2	833.7351	1.93	3	28.8	78.8	1	1036-1064	K.GDRGESGPAGPPGAPGAPGAPGPVGPAGK.S	Oxidation: 12, 15, 18, 21
743	2	724.3523	2.82	3	31.0	59.3	0	1039-1064	R.GESGPAGPPGAPGAPGAPGPVGPAGK.S	Oxidation: 9, 12, 15, 18
843	2	654.6663	2.85	3	33.1	89.2	1	1065-1086	K.SGDRGETGPAGPAGPVGPVGAR.G	
987	2	773.9031	1.12	2	35.9	104.4	0	1069-1086	R.GETGPAGPAGPVGPVGAR.G	

1319	2	902.4254	2.50	3	42.8	118.7	0	1114-1143	R.GFSGLQGPPGPPGSPGEQGPSGASGPAGPR.G	Oxidation: 11, 12, 15
1531	1	781.8956	1.87	2	47.2	26.7	0	1155-1170	K.DGLNGLPGPIGPPGPR.G	Oxidation: 9, 12, 13; Deamidated: 4
1406	1	781.4026	0.65	2	44.6	46.1	0	1155-1170	K.DGLNGLPGPIGPPGPR.G	Oxidation: 7, 13, 15
1412	2	781.4032	1.36	2	44.7	48.2	0	1155-1170	K.DGLNGLPGPIGPPGPR.G	Oxidation: 7, 12, 15

8

Detailed Protein Report

LC-MS/MS analysis

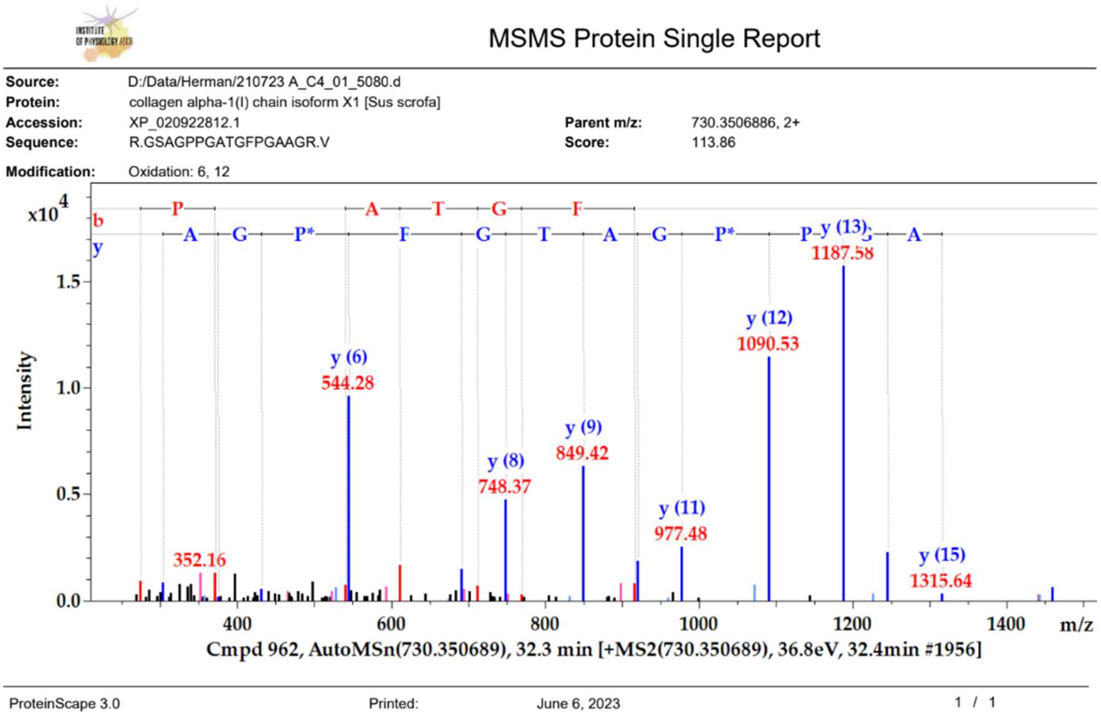

LC-MS/MS analysis of collagen type I, chain 1. Representative MS/MS spectra of tryptic peptide identified as collagen type I, chain 1 (GSAGPPGATGFPGAAGR). Oxidation (hydroxyproline) is marked by an asterix.

Detailed Protein Report

MSMS Protein Single Report

**Source:**	D:/Data/Herman/210723 A_C4_01_5080.d		
**Protein:**	alpha2 chain of type I collagen [Sus scrofa domesticus]		
**Accession:**	BAX02569.1	**Parent m/z:**	775.8823315, 2+
**Sequence:**	R.GPPGESGAAGPAGPIGSR.G	**Score:**	127.95
**Modification:**	Oxidation: 3		

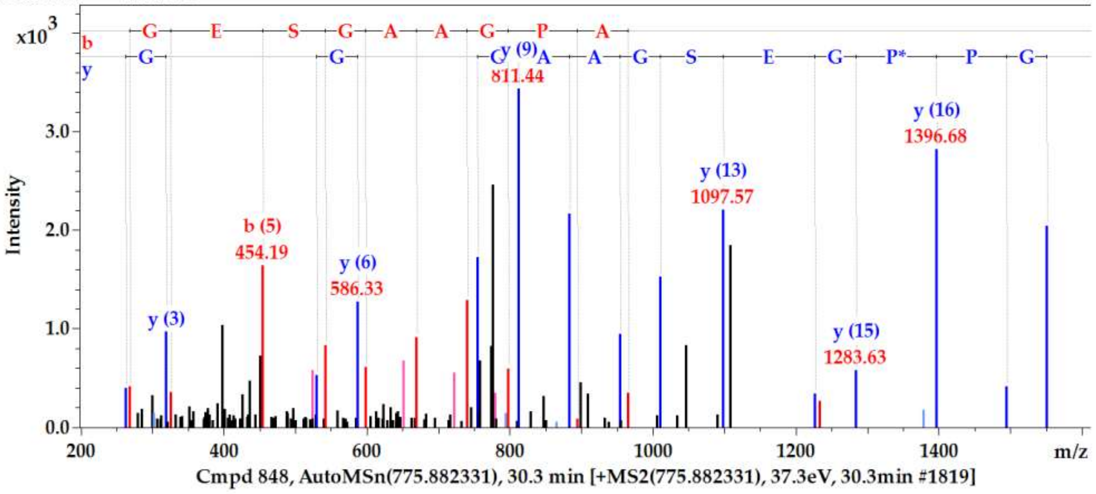

ProteinScape 3.0 Printed: June 6, 2023 1 / 1

LC-MS/MS analysis of collagen type I, chain 2. Representative MS/MS spectra of tryptic peptide identified as collagen type I, chain 2 (GPPGESGAAGPAGPIGSR). Oxidation (hydroxyproline) is marked by an asterix.

Detailed Protein Report

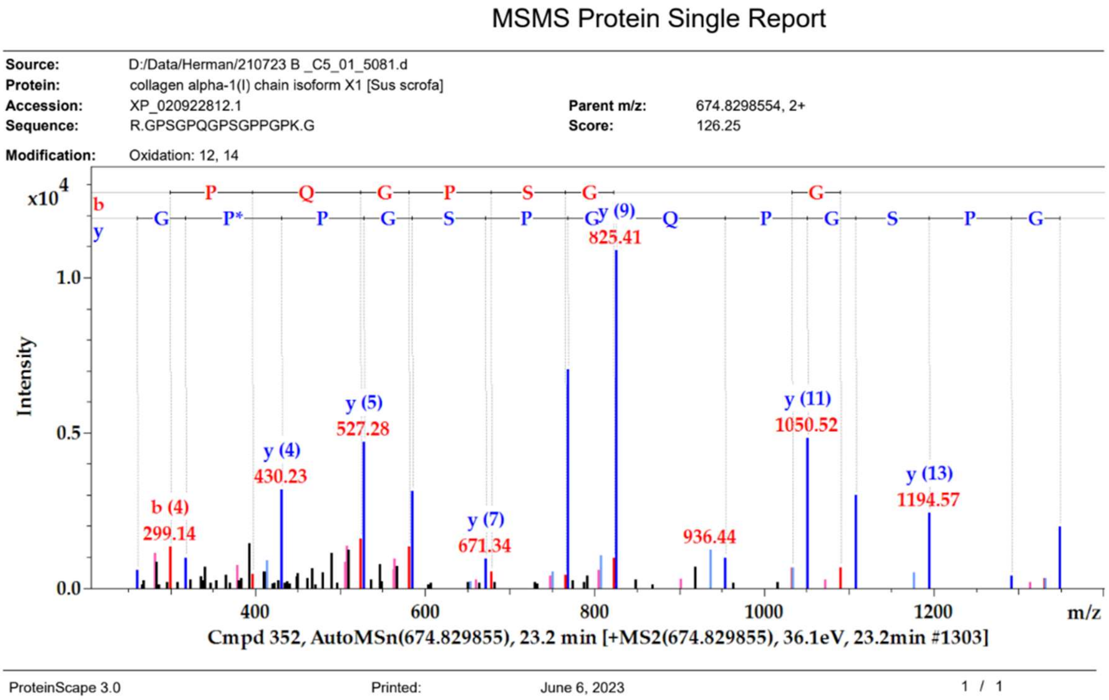

LC-MS/MS analysis of collagen type I, chain 1. Representative MS/MS spectra of tryptic peptide identified as collagen type 1, chain 1 (GPSGPQGPSGPPGPK). Oxidation (hydroxyproline) is marked by an asterix.

Detailed Protein Report

MSMS Protein Single Report

**Source:**	D:/Data/Herman/210723 B _C5_01_5081.d		
**Protein:**	collagen alpha-1(III) chain precursor [Sus scrofa]		
**Accession:**	NP_001230226.1	**Parent m/z:**	704.8290495, 2+
**Sequence:**	R.GPPGPPGTNGAPGQR.G	**Score:**	88.65
**Modification:**	Oxidation: 2, 6, 12; Deamidated: 9		

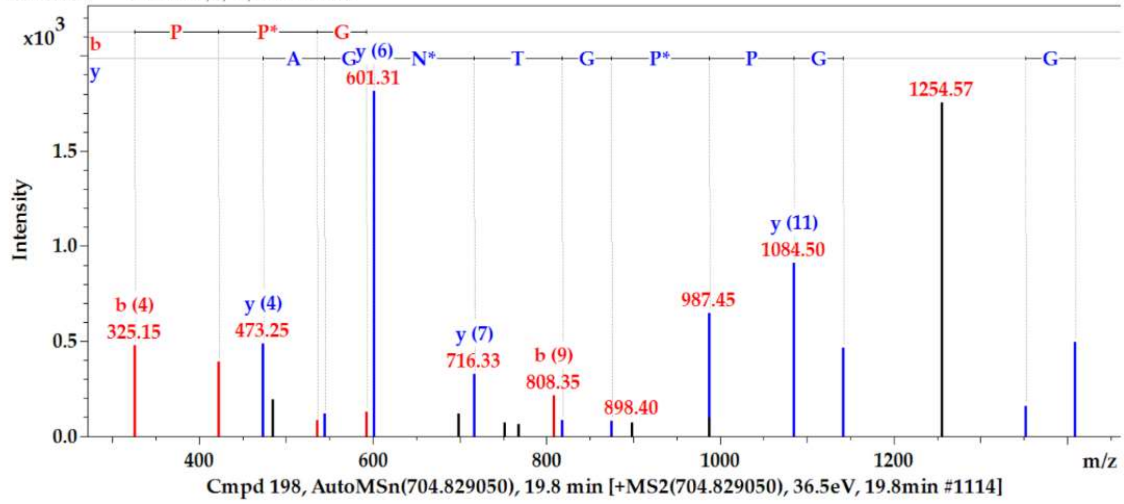

ProteinScape 3.0 Printed: June 6, 2023 1 / 1

LC-MS/MS analysis of collagen type III, chain 1. Representative MS/MS spectra of tryptic peptide identified as collagen type III, chain 1 (GPPGPPGTNGAPGQR). Oxidation (hydroxyproline) is marked by an asterix.

Detailed Protein Report

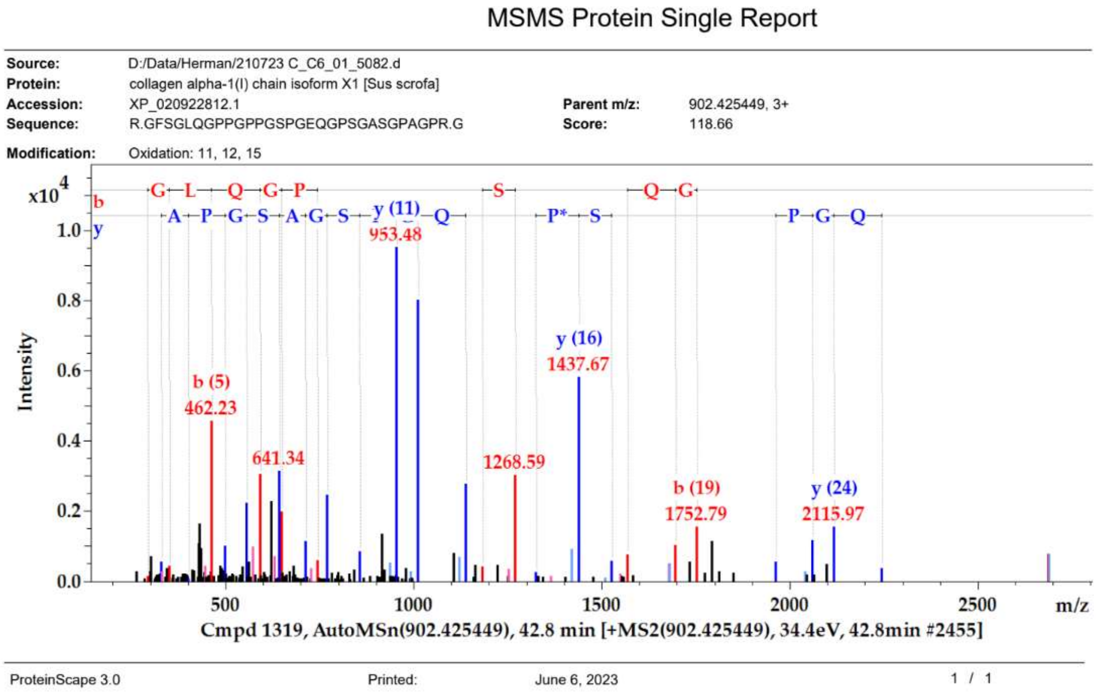

LC-MS/MS analysis of collagen type I, chain 1. Representative MS/MS spectra of tryptic peptide identified as collagen type I, chain 1 (GFSGLQGPPGSPGEQGPSGASGPAGPR). Oxidation (hydroxyproline) is marked by an asterix.

Detailed Protein Report

MSMS Protein Single Report

Source: D:/Data/Herman/210723 C_C6_01_5082.d
Protein: collagen alpha-2(I) chain precursor [Sus scrofa]
Accession: NP_001230584.1 **Parent m/z:** 775.8828274, 2+
Sequence: R.GPPGESGAAGPAGPIGSR.G **Score:** 136.39
Modification: Oxidation: 3

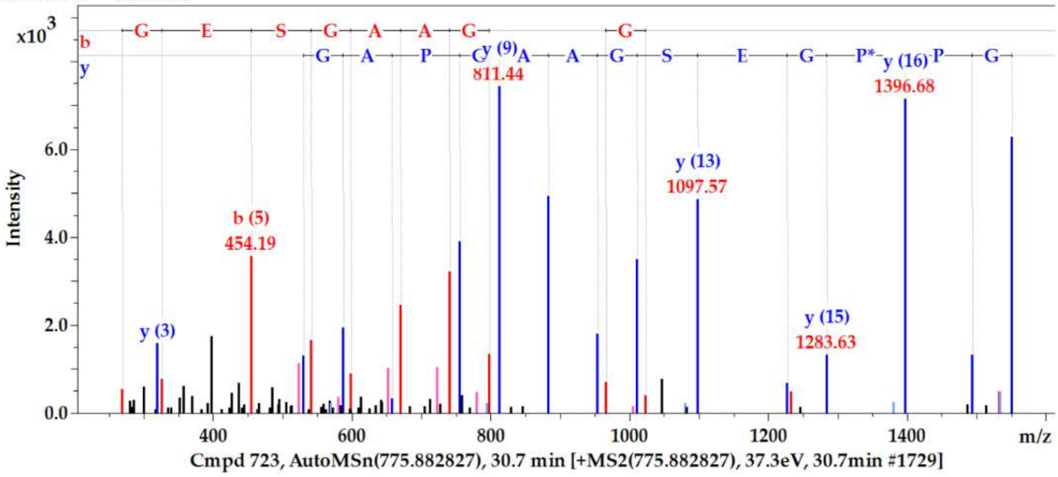

ProteinScape 3.0 Printed: June 6, 2023 1 / 1

LC-MS/MS analysis of collagen type I, chain 2. Representative MS/MS spectra of tryptic peptide identified as collagen type I, chain 2 (GPPGESGAAGPAGPIGSR). Oxidation (hydroxyproline) is is marked by an asterix.
