# Supplementary material for: Discovery of mammalian collagens I and III within ancient poriferan biopolymer spongin
Source: Nat Commun. 2025 Mar 13;16:2515. doi: 10.1038/s41467-025-57460-y (PMC11906918; doi:10.1038/s41467-025-57460-y)
Supplement: Supplementary file 4 — Reporting Summary [file 41467_2025_57460_MOESM4_ESM.pdf]

nature portfolio

Corresponding author(s): Hermann Ehrlich, Markus Buehler

Last updated by author(s): Feb 15, 2025

Reporting Summary

Nature Portfolio wishes to improve the reproducibility of the work that we publish. This form provides structure for consistency and transparency in reporting. For further information on Nature Portfolio policies, see our Editorial Policies and the Editorial Policy Checklist.

Statistics

For all statistical analyses, confirm that the following items are present in the figure legend, table legend, main text, or Methods section.

n/a	Confirmed	
☐	☒	The exact sample size (n) for each experimental group/condition, given as a discrete number and unit of measurement
☐	☒	A statement on whether measurements were taken from distinct samples or whether the same sample was measured repeatedly
☒	☐	The statistical test(s) used AND whether they are one- or two-sided<br>*Only common tests should be described solely by name; describe more complex techniques in the Methods section.*
☒	☐	A description of all covariates tested
☒	☐	A description of any assumptions or corrections, such as tests of normality and adjustment for multiple comparisons
☒	☐	A full description of the statistical parameters including central tendency (e.g. means) or other basic estimates (e.g. regression coefficient) AND variation (e.g. standard deviation) or associated estimates of uncertainty (e.g. confidence intervals)
☒	☐	For null hypothesis testing, the test statistic (e.g. F, t, r) with confidence intervals, effect sizes, degrees of freedom and P value noted<br>*Give P values as exact values whenever suitable.*
☒	☐	For Bayesian analysis, information on the choice of priors and Markov chain Monte Carlo settings
☐	☒	For hierarchical and complex designs, identification of the appropriate level for tests and full reporting of outcomes
☒	☐	Estimates of effect sizes (e.g. Cohen's d, Pearson's r), indicating how they were calculated

Our web collection on statistics for biologists contains articles on many of the points above.

Software and code

Policy information about availability of computer code

Data collection	The nLC-MS/MS instruments were controlled with the software packages HyStar 3.2 and micrOTOF-control 3.0, the data were collected and manipulated with the software packages ProteinScape 3.0 and DataAnalysis 4.0 (Bruker Daltonics).<br>All MD simulations were executed using the NAMD v3b software package with the CHARMM36m forcefield.
Data analysis	Spectroscopic data were analyzed and displayed with MATLAB (MathWorks Inc., Natick, USA).<br>The nLC-MS/MS data were processed using ProteinScape software v. 3.0.0.446 (Bruker Daltonics, Bremen, Germany) using NCBI database (downloaded on July 4, 2020; 133,126 sequences; 105,045,692 residues) using the MASCOT search engine v. 2.3.0 (http://www.matrixscience.com). Control searching was performed on the whole SwissProt database (downloaded on July 4, 2020; 562,755 sequences; 202,599,198 residues).<br>The BLAST-2.15.0+ toolkit (https://blast.ncbi.nlm.nih.gov/doc/blast-help/downloadblastdata.html44) was used to identify sequences similar to the proteins identified in spongin by LC-MS/MS or nucleotide sequences encoding them.<br>As outlined in the computational modeling section of the Methods, we used the PDB structures 7CWK and 8HHI to model a system that mimics spongin for investigation by means of molecular dynamics. These structures are publicly available on the PDB website, where their validation and assessment details can be accessed. In the manuscript and Supplementary Information, we explain how we used standard atomistic modeling techniques to model the different cross-links (with 1, 2, and 3 Bromine atoms, respectively) between the structures. Additionally, we have shared the final modeled PDB structure files. We believe that the information provided is sufficient for reproducing the models.

For manuscripts utilizing custom algorithms or software that are central to the research but not yet described in published literature, software must be made available to editors and reviewers. We strongly encourage code deposition in a community repository (e.g. GitHub). See the Nature Portfolio guidelines for submitting code & software for further information.

Data

Policy information about availability of data

All manuscripts must include a data availability statement. This statement should provide the following information, where applicable:

- Accession codes, unique identifiers, or web links for publicly available datasets
- A description of any restrictions on data availability
- For clinical datasets or third party data, please ensure that the statement adheres to our policy

The data availability statement (DAS) is included in the manuscripts main text:
Data availability
The molecular dynamics data generated in this study have been deposited in the github.com database [https://github.com/albertomds/collagen]. The proteomics LC-MS data generated in this study have been deposited in the ProteomeXchange partner repository MassIVE, ProteomeXchange dataset PXD060839 [https://doi.org/10.25345/C58P5VN6J], as well as to the zenodo.org database [https://doi.org/10.5281/zenodo.14677365]. The ESI-MS, HPLC, UV-VIS data are available in the zenodo.org database [https://doi.org/10.5281/zenodo.14741873]. The Solid State 13C NMR data, FTIR and Raman spectroscopy of spongin data generated for this study as well as the unprocessed scans of SDS-PAAG and Western blots are provided in the Source Data file. Detailed protein report (Mascot search results and LC-MS/MS analysis) is available in Supplementary Data file. The genomes used for BLAST search in this study are available in the NCBI database under following accession codes: GCA_000090795.2 [https://www.ncbi.nlm.nih.gov/datasets/genome/GCF_000090795.2/] (Amphimedon queenslandica), GCA_949841015.1 [https://www.ncbi.nlm.nih.gov/datasets/genome/GCA_949841015.1/] (Aplysina aerophoba), GCA_947172415.1 [https://www.ncbi.nlm.nih.gov/datasets/genome/GCA_947172415.1/] (Chondrosia reniformis), GCA_013339895.1 [https://www.ncbi.nlm.nih.gov/datasets/genome/GCA_013339895.1/] (Ephydatia muelleri), GCA_020423275.1 [https://www.ncbi.nlm.nih.gov/datasets/genome/GCA_020423275.1/] (Halichondria panicea), GCA_947507565.1 [https://www.ncbi.nlm.nih.gov/datasets/genome/GCA_947507565.1/] (Oscarella lobularis), GCA_947044365.1 [https://www.ncbi.nlm.nih.gov/datasets/genome/GCA_947044365.1/] (Petrosia ficiformis). The reference protein sequences used in this study for the Mascot search and BLAST analysis are available in the NCBI database under accession codes: XP_020922812.1 [https://www.ncbi.nlm.nih.gov/protein/XP_020922812.1] (Collagen I alpha-1 chain isoform X1 [Sus scrofa]), BAX02569.1 [https://www.ncbi.nlm.nih.gov/protein/BAX02569.1] (Alpha 2 chain of type I collagen [Sus scrofa domesticus]), XP_020922812.1 [https://www.ncbi.nlm.nih.gov/protein/XP_020922812.1] (collagen alpha-1 (I) chain isoform X1 [Sus scrofa]), NP_001230226.1 [https://www.ncbi.nlm.nih.gov/protein/NP_001230226.1] (Collagen alpha-1 (III) chain precursor [Sus scrofa]), NP_001230584.1 [https://www.ncbi.nlm.nih.gov/protein/NP_001230584.1] (collagen alpha-2 (I) chain precursor [Sus scrofa]), XP_020922812.1 [https://www.ncbi.nlm.nih.gov/protein/XP_020922812.1] (Collagen alpha-1(I) chain isoform X1 [Sus scrofa]), CAQ63561.1 https://www.ncbi.nlm.nih.gov/protein/CAQ63561.1 (fibrillar collagen COL5alpha, partial [Amphimedon queenslandica]), CAQ63562.1 [https://www.ncbi.nlm.nih.gov/protein/CAQ63562.1] (fibrillar collagen COL6alpha, partial [Amphimedon queenslandica]), XP_052314686.1 [https://www.ncbi.nlm.nih.gov/protein/XP_052314686.1] (Collagen alpha-1(I) chain-like [Oncorhynchus keta]), KAJ7374653.1 [https://www.ncbi.nlm.nih.gov/protein/KAJ7374653.1] (Kinesin-like protein kif15 [Desmophyllum pertusum]), XP_034534652.1 [https://www.ncbi.nlm.nih.gov/protein/XP_034534652.1] (LOW QUALITY PROTEIN: collagen alpha-1(IX) chain-like [Notolabrus celidotus]), P18503.1 [https://www.ncbi.nlm.nih.gov/protein/P18503.1] (short-chain collagen C4 [Ephydatia muelleri]), XP_019854257.1 [https://www.ncbi.nlm.nih.gov/protein/XP_019854257.1] (collagen alpha-1(I) chain [Amphimedon queenslandica]), XP_020906601.1 [https://www.ncbi.nlm.nih.gov/protein/XP_020906601.1] (Collagen alpha-1(I) chain [Exaiptasia diaphana]), CAI8027724.1 [https://www.ncbi.nlm.nih.gov/protein/CAI8027724.1] (Collagen alpha-1(XXIV) chain [Geodia barretti]). Unless otherwise stated, all data supporting the results of this study can be found in the article, supplementary, and source data files. Source Data are provided with this paper.

Research involving human participants, their data, or biological material

Policy information about studies with human participants or human data. See also policy information about sex, gender (identity/presentation), and sexual orientation and race, ethnicity and racism.

Reporting on sex and gender	No human participants were involved in this study. Human collagen standarts type I, III, and IV (p. n. C7774, C4407, and C5533, respectively) were purchased from Sigma Aldrich.
Reporting on race, ethnicity, or other socially relevant groupings	No human participants were involved in this study.
Population characteristics	No human participants were involved in this study.
Recruitment	No human participants were involved in this study.
Ethics oversight	There are no ethical aspects within this study.

Note that full information on the approval of the study protocol must also be provided in the manuscript.

Field-specific reporting

Please select the one below that is the best fit for your research. If you are not sure, read the appropriate sections before making your selection.

☒ Life sciences ☐ Behavioural & social sciences ☐ Ecological, evolutionary & environmental sciences

For a reference copy of the document with all sections, see nature.com/documents/nr-reporting-summary-flat.pdf

Life sciences study design

All studies must disclose on these points even when the disclosure is negative.

Sample size	Samples of the Hippospongia communis sponge were measured using conventional measuring tools including caliper etc.
Data exclusions	The Peptide Decoy option was selected during the data search process to remove false positive results of proteomics investigation. Only significant hits were accepted (MASCOT score≥80 for proteins and MASCOT score ≥20 for peptides, http://www.matrixscience.com)
Replication	Experiments were replicated at least 3 times and all replications were successfull.
Randomization	The Hippospongia communis samples were selected randomly for each experiment oriented on collagen identification.
Blinding	In our study, which focused on the characterization of spongin as a protein, blinding was not relevant. The research primarily involved biochemical and structural analysis of spongin, rather than experiments involving subjective assessments or treatment groups where bias might influence the results. As such, blinding of investigators to group allocation was not applicable to the study design.

Reporting for specific materials, systems and methods

We require information from authors about some types of materials, experimental systems and methods used in many studies. Here, indicate whether each material, system or method listed is relevant to your study. If you are not sure if a list item applies to your research, read the appropriate section before selecting a response.

Materials & experimental systems

n/a	Involved in the study	
☐	☒	Antibodies
☒	☐	Eukaryotic cell lines
☒	☐	Palaeontology and archaeology
☐	☒	Animals and other organisms
☒	☐	Clinical data
☒	☐	Dual use research of concern
☒	☐	Plants

Methods

n/a	Involved in the study	
☒	☐	ChIP-seq
☒	☐	Flow cytometry
☒	☐	MRI-based neuroimaging

Antibodies

Antibodies used	Collagen I Polyclonal Antibody<br>Collagen III Polyclonal Antibody
Validation	Collagen I Polyclonal Antibody<br>Producer: Invitrogen<br>Catalog Number: PA5-95137<br>RRID: AB_2806942<br>dilution 1:1000<br>Collagen III Polyclonal Antibody<br>Producer: Invitrogen<br>Catalog Number: PA5-27828<br>RRID: AB_2545304<br>dilution 1:1000

Animals and other research organisms

Policy information about studies involving animals; ARRIVE guidelines recommended for reporting animal research, and Sex and Gender in Research

Laboratory animals	The study did not involve laboratory animals
Wild animals	The study did not involve wild animals
Reporting on sex	The information has not been collected
Field-collected samples	The study did not involve sample collection from the field
Ethics oversight	No ethical approval or guidance was required, as we used only dried acellular samples of spongin from the sponge Hippospongia communis obtained in an industrially harvested form intended for cosmetic use

Note that full information on the approval of the study protocol must also be provided in the manuscript.

Plants

Seed stocks	The study did not involve plants
Novel plant genotypes	*Describe the methods by which all novel plant genotypes were produced. This includes those generated by transgenic approaches, gene editing, chemical/radiation-based mutagenesis and hybridization. For transgenic lines, describe the transformation method, the number of independent lines analyzed and the generation upon which experiments were performed. For gene-edited lines, describe the editor used, the endogenous sequence targeted for editing, the targeting guide RNA sequence (if applicable) and how the editor was applied.*
Authentication	*Describe any authentication procedures for each seed stock used or novel genotype generated. Describe any experiments used to assess the effect of a mutation and, where applicable, how potential secondary effects (e.g. second site T-DNA insertions, mosiacism, off-target gene editing) were examined.*
