# Supplementary material for: Discovery of mammalian collagens I and III within ancient poriferan biopolymer spongin
Source: Nat Commun. 2025 Mar 13;16:2515. doi: 10.1038/s41467-025-57460-y (PMC11906918; doi:10.1038/s41467-025-57460-y)
Supplement: Supplementary file 5 — Transparent Peer Review file [file 41467_2025_57460_MOESM5_ESM.pdf]

nature portfolio

Peer Review File

Discovery of mammalian collagens I and III within ancient poriferan biopolymer spongin

Corresponding Author: Professor Hermann Ehrlich

This file contains all reviewer reports in order by version, followed by all author rebuttals in order by version.

Version 0:

Reviewer comments:

Reviewer #1

(Remarks to the Author)
This paper presents multi-technique evidence of the presence of Collagen I and Collagen III in spongin, the biopolymer forming the skeleton of sea sponges (Porifera).

The evidence for the presence of fibrillar proteins in spongin includes TEM and Second Harmonic Generation, and the evidence specifically for Collagens I and III includes proteomics as well as C-13 NMR. Overall, I find the body of evidence convincing, although the identification of the two collagens could potentially be still not 100% certain.

The discovery of Collagens I and III in sea sponges is highly significant, both from the point of view of development of novel biocomposites and from the point of view of evolutionary biology. As the authors point out, sea sponges are one of the oldest metazoan life forms on Earth, preceding animals such as fish or amphibians by hundreds of millions of years. Therefore, the present results are an important building block in the understanding of evolutionary history of fibrillar proteins and extracellular matrix. Limited evidence of the presence of Collagen I in spongin has been available before (see e.g. ref. 6). However, the present study is a significant step forward from prior knowledge because it presents in-depth, multi-technique characterisation of Collagens I and III in spongin.

While the present paper answers some important questions concerning the make-up of spongin, it also creates a foundation for future research, for example as to the exact role and the evolutionary history of halogens in the collagen structure.

Specific comments:

1) The authors use C^(13) (C with the upper-case 13 after it) to refer to carbon NMR, this notation is odd in that it is not normally used in the NMR literature. The usual notations are ^(13)C (the upper-case 13 precedes C) or simply C-13.

2) MD simulations: Was there any particular reason why TIP3P water was used? More recent water models, e.g. TIP4P/2005, are commonly used the latest literature. What was the rationale for using TIP3P, and are different water models expected to materially affect the results?

3) Have the authors explored the possibility that the proteins discovered could be other proteins with the same NMR and proteomic signatures as Collagens I or III? On the scale of 0 to 100, how “positive” is the identification of these two collagens? Would additional techniques (perhaps Immunohistochemistry?) add more certainty to the collagen identification?

4) It is odd that the there is no Discussion section in the manuscript. If this a journal requirement? There is a brief Discussion section present on p. 14, but there is no heading identifying it as such. My preference would be to add a “Discussion” heading.

5) The captions of Table 1 and Fig 4 are swapped.

Reviewer #2

(Remarks to the Author)
Review of Manuscript: NCOMMS-24-50188
Title: Discovery of mammalian collagens I and III within ancient poriferan biopolymer spongin
Author(s): Ehrlich et al.

The leading author, Prof. Ehrlich gathered (again) a large panel of experts in various techniques, tools and analyses, to tackle a major question regarding the composition of sponge spongin components, and their relatedness to collagens of other animal phyla. The topic of the manuscript is therefore suitable to Nat. Comm. readership, but the content requires revision before accepted to be published. I have to emphasize that I read the entire paper including the supplementary material, but am qualified to expertly assess only some of the aspects reported here (as explained to the editor).
The complimentary techniques and approaches employed, led to the identification of collagen I & II in spongin fibers. In addition, specific cropsslinking agents were discovered, that also contribute to the understanding of the molecules function.
It is clear that the manuscript requires the input of an English (Scientific) writer/editor. Bellow I will mention only some examples.
Bellow I suggest some comments and present questions for the authors to address (in order of appearance – not importance).

Line 35 - "In other 35 words, sponges are a point of origin for " Sponges diverged close to the origin of all other.
Line 42 - Delete "Keratosan sponges of".
Line 42 - Replace "are" with "containes".
Line 44 – Replace "skeletons of these poriferans are" with skeleton of sponges is"
Line 172 – I found no reference to the importance/meaning of the distribution as appears in Fig 3i.
Line 182-184 - Proteomics is not "a method". It certainly is not aimed at "identifying corresponding collagen chains". This sentence should be rephrased.
Line 192 & Line 193 - The table and figure legends are mixed.
Lines 229-232 - I disagree with the statement that in healthy sponge the spongin skeleton is exposed to seawater. Usually it is inside, covered by the mesohyl.
Lines 317-318 - I suggest to tune down this statement. The fact that collagens exist in sponges and other animals does not position sponges as the archaeal source of collagen(s), but that they all originated from the same root.
Lines 330-331 - I really liked this concluding remark.
Between lines 338 and 490, no reference is mentioned to support, or describe, or justify, any of the methods used.
Line 399 - Do you mean "proteins ...... were" solubilized.."?
Lines 529 - 626 - Ref 1 & 3-45 are without doi address, while the rest are with. Please decide.
Lines 564 & 569 - The same journal is cited twice differently.
Line 571 - Change to J. Spectros.
Line 586 - The name of the journal Nature, should not be abbreviated.
Line 601 - Why did the authors cite ref 42 in full (all 18 authors) while, ref 26, 43 and 44 (with 6, 8 and 7 authors respectively) are abbreviated to et al.

Reviewer #3

(Remarks to the Author)
In their manuscript entitled „Discovery of mammalian collagens I and III within ancient poriferan biopolymer spongin", the authors Hermann Ehrlich, Markus Buehler et al., report on the structural composition of sponging, a skeletal compound of the first metazoan organisms. Using an integrated set of analytical techniques including vibrational spectroscopic, electron microscopy, NMR and combining it with molecular dynamics, the authors show that the biopolymer is composed of collagen I and III with dedicated crosslinking. This discovery is used to proposed some discussion on the origin of some collagen domains present in mammals in general and in humans in particular that are already found in this very ancient organism.
I have only minor points to note:
• Most of the labels present in figure 3 are simply not readable when depicted at 100% scale (so on a printed version). Please modify to make it readable.
• The caption of Figure 4 appears on line 193, it should be on line 192 (directly after the figure, but before the table).
• Line 338, I think “imagery” should be “imaging”.
• Line 421, liter is wrongly abbreviated with small “l” here, it is capital “L” as everywhere else in the manuscript.
• Article or page numbers are missing in the reference section.
• Fig SI3.1, please modify the scale bar to 100 nm instead of 0.1 µm for consistency.
• Fig. SI 7.2, the scale bar is present, but no indication of the scale is given.
Looking forward to have this manuscript published
Damien Faivre

Reviewer #4

(Remarks to the Author)
This manuscript seeks to answer fundamental questions about spongin chemical and material nature using a broad range of molecular and chemical tools. The paper presents a thorough investigation that identified collagens I and III as the main structural components of spongin fibers using proteomics, Western blot, translated nucleotide BLAST method, C 13 solid state NMR, and Raman spectroscopy. The complex interplay of collagen molecular structures and crosslinks was characterized using molecular dynamic modeling based upon the HPLC-MS analysis of spongin collagen samples. The work is impressive and answers fundamental questions about collagen structure and crosslinking in spongin particularly but provides a fundamental basis for collagen chemistry and material properties. Prior to publication in Nature Communications, this work requires some modifications. Please see the major and minor comments:
Major Comments:
1. The data in Figure 4 and Table 1 as presented is confusing. It is unclear what various columns in Table 1 are referring to, particularly the columns titled, 'Scores' and SC (%). The data in Figure 4, includes the SDS-Page data of the 3 different collagen proteins listed in the Table 1. It is not clearly mentioned but the 210723 A band seems to be a dimerization of both the collagen alpha-1(I) chain isoform X1 [Sus scrofa] and the Alpha 2 chain of type I collagen [Sus scrofa domesticus] whereas the other bands B & C are not dimers based on the reported molecular weights. In panels (a), (b), and (c) of Figure 4, individual bands are reported to be labeled with collagen I and collagen III antibodies based on different preparation methods of disintegrated spongin samples. The molecular weights of the labeled bands are much different than typically reported molecular weights for collagens I and III and do not correspond to the molecular weights provided in Table 1. Please increase clarity regarding this figure and its role in supporting your conclusions.
Minor Comments:
1. Column 1 in Figure 6 which shows the 3D visualizations of the cross talks are color coded. Please add to the legend that the purple color represents the bromide atoms.
2. Line 341: What are the percentages of ethanol used in the series and for how long is the sample left in each ethanol solution?
3. Line 349: How long were the samples dried at 37C?
4. Line 387: How much spongin powder was added incrementally every 3 hours to get to the final concentration?
5. Line 403: What gel chemistry was used for the polyacrylamide gel?
6. Line 404: What was the specific running buffer used?
7. Lines 467 & 474: What was the specific transfer buffer used?
8. More information on the instrument settings of TEM and FTIR imaging is needed.

Reviewer #5

(Remarks to the Author)
I co-reviewed this manuscript with one of the reviewers who provided the listed reports. This is part of the Nature Communications initiative to facilitate training in peer review and to provide appropriate recognition for Early Career Researchers who co-review manuscripts.

Version 1:

Reviewer comments:

Reviewer #1

(Remarks to the Author)
This is the revised version of the paper demonstrating the presence of Collagen I and Collagen III in spongin, the biopolymer forming the skeleton of sea sponges (Porifera), using a wide range of experimental and computational techniques.

The revision addresses my original comments well, and I am fully satisfied with the changes and/or clarifications made. In my view, the work itself and the subsequent analysis are meticulous and thoughtful. While the identification of Collagens I and III in spongin is still not absolutely certain, in my view there are sufficient groundbreaking findings in this paper to merit its publication in Nature Communications. I therefore recommend Acceptance.

Specifically with regards to computational MD studies, I think the methodology used is highly competent and instils confidence in the conclusions made. I agree that TIP3P water model is a valid choice that provides a good trade-off between accuracy and efficiency, and there is good evidence that the important results would not be sensitive to the choice of the water model.

One recommendation I would like to make for future work is to explore Bromine NMR for the purpose of using brominated Tyrosine as a spectrally uncrowded probe of the molecular configuration and dynamics of the collagens. Bromine has two stable isotopes, Br-79 and Br-81, each with close to 50% natural abundance and similar NMR properties; there are pros and cons for using either isotope for specific tasks (e.g. collagen identification, cross-link geometry or molecular dynamics). I am not suggesting that this work should be performed for the present paper; rather, this is a suggestion for future work.

Reviewer #2

(Remarks to the Author)
I am satisfied with the corrections made, and recommend publication of the manuscript.
One minor correction yet to be made, is to put the Table caption above the table (unlike the figure legends legend).

Reviewer #3

(Remarks to the Author)
All my points have been taken into consideration, i look forward to have the paper published

Reviewer #4

(Remarks to the Author)
The authors did a phenomenal job in responding to the reviewer questions and clearly incorporating the responses into the article. There is still some additional details involving Figure 4 that will improve clarity. From the figure caption, it can be inferred that Figure 4a is collagen I while Figure 4c is collagen III. Figure 4b is less clear where the authors state "Identification of collagens I and III in this spongin with Western Blot is represented in Fig (b)." Thus, more clarification of antibodies used is needed for Figure 4b. Furthermore, the authors do not address why Sample A (spongin at pH 7.1) has stronger bands than Sample B (spongin at pH 2.3) in Figure 4a (collagen I) while the opposite is true in Figure 4c (collagen III).

Reviewer #5

(Remarks to the Author)
I co-reviewed this manuscript with one of the reviewers who provided the listed reports. This is part of the Nature Communications initiative to facilitate training in peer review and to provide appropriate recognition for Early Career Researchers who co-review manuscripts.

Open Access This Peer Review File is licensed under a Creative Commons Attribution 4.0 International License, which permits use, sharing, adaptation, distribution and reproduction in any medium or format, as long as you give appropriate credit to the original author(s) and the source, provide a link to the Creative Commons license, and indicate if changes were made.
In cases where reviewers are anonymous, credit should be given to 'Anonymous Referee' and the source.
The images or other third party material in this Peer Review File are included in the article’s Creative Commons license, unless indicated otherwise in a credit line to the material. If material is not included in the article’s Creative Commons license and your intended use is not permitted by statutory regulation or exceeds the permitted use, you will need to obtain permission directly from the copyright holder.
To view a copy of this license, visit https://creativecommons.org/licenses/by/4.0/

REVIEWER COMMENTS

Reviewer #1 (Remarks to the Author):

This paper presents multi-technique evidence of the presence of Collagen I and Collagen III in spongin, the biopolymer forming the skeleton of sea sponges (Porifera).

The evidence for the presence of fibrillar proteins in spongin includes TEM and Second Harmonic Generation, and the evidence specifically for Collagens I and III includes proteomics as well as C-13 NMR. Overall, I find the body of evidence convincing, although the identification of the two collagens could potentially be still not 100% certain.

The discovery of Collagens I and III in sea sponges is highly significant, both from the point of view of development of novel biocomposites and from the point of view of evolutionary biology. As the authors point out, sea sponges are one of the oldest metazoan life forms on Earth, preceding animals such as fish or amphibians by hundreds of millions of years. Therefore, the present results are an important building block in the understanding of evolutionary history of fibrillar proteins and extracellular matrix. Limited evidence of the presence of Collagen I in spongin has been available before (see e.g. ref. 6). However, the present study is a significant step forward from prior knowledge because it presents in-depth, multi-technique characterisation of Collagens I and III in spongin.

While the present paper answers some important questions concerning the make-up of spongin, it also creates a foundation for future research, for example as to the exact role and the evolutionary history of halogens in the collagen structure.

Response: We appreciate your critical remarks as well as recommendations!

Specific comments:

1) The authors use C^(13) (C with the upper-case 13 after it) to refer to carbon NMR, this notation is odd in that it is not normally used in the NMR literature. The usual notations are ^(13)C (the upper-case 13 precedes C) or simply C-13.

Response: Thank you for your remark, we have changed the notation to ^{13}C (the upper-case 13 precedes C).

2) MD simulations: Was there any particular reason why TIP3P water was used? More recent water models, e.g. TIP4P/2005, are commonly used the latest literature. What was the rationale for using TIP3P, and are different water models expected to materially affect the results?

Response: We thank the reviewer for this comment. Historically, TIP3P has been widely used in protein studies because of its efficiency, sufficient accuracy, and compatibility with many standard force fields (e.g., CHARMM, AMBER). It remains a default water model in many protein simulations in recent studies, such as https://doi.org/10.1021/acs.biomac.4c00183, where TIP3P was successfully used to investigate collagen hydration and bulk water properties, and https://doi.org/10.1021/acsbiomaterials.2c00728, where TIP3P was utilized to study the interaction of water with collagen and hydroxyapatite, revealing specific local water disruptions caused by ion coordination.

While the TIP4P family of water models (e.g., TIP4P/2005) is more accurate in reproducing certain bulk water properties (e.g., density, diffusion, and dielectric constants)

[https://pubs.acs.org/doi/full/10.1021/acs.jcim.1c00794, DOI:10.3390/polym13020176], it demands higher computational cost, especially for large or complex systems. The TIP3P water model, on the other hand, offers a good trade-off between accuracy and computational efficiency, without compromising the quality of the protein dynamics we focus on. Although TIP4P/2005 may better capture some aspects of water behavior, a study using this water model found no significant differences in collagen dynamics or interactions with water (https://doi.org/10.1021/acs.jpcb.9b03078).

Given our focus on protein dynamics rather than detailed water behavior, the TIP3P model has proven sufficient and computationally feasible for our study. We have provided further clarification on this point in the revised manuscript, particularly in the Supplementary Information section, where we state:

"We initiated our study by constructing **the collagen types I and III** complex, capitalizing on the pre-existing hydrophobic groups. Specifically, we leveraged phenylalanine residues (later replaced with TYR) as interaction points within the sequences. Our computational investigation included 51 ns of MD simulations across four distinct systems: no crosslink, 1Br, 2Br, and 3Br crosslinks. **For these simulations, we used the TIP3P water model, chosen for its computational efficiency and accuracy in capturing the relevant collagen protein dynamics, as supported by recent studies on similar systems (https://doi.org/10.1021/acs.biomac.4c00183 and https://doi.org/10.1021/acsbiomaterials.2c00728)**" …

3) Have the authors explored the possibility that the proteins discovered could be other proteins with the same NMR and proteomic signatures as Collagens I or III? On the scale of 0 to 100, how "positive" is the identification of these two collagens? Would additional techniques (perhaps Immunohistochemistry?) add more certainty to the collagen identification?

Response: We appreciate this important observation. To minimize the possibility that the proteins identified could be other proteins with similar NMR and proteomic signatures, we employed complementary techniques, including SDS-PAGE and mass spectrometry, and confirmed the presence of Collagen I and III using Western blot with specific antibodies. The proteomic and NMR results demonstrated clear alignment with the known properties of Collagen I and III. On a scale of 0 to 100, we estimate the certainty of the collagen identification to be above 90, acknowledging that there is always a small margin for ambiguity in biochemical analyses.

To further enhance the certainty of these findings, we agree that Immunohistochemistry could add value by localizing the collagen in situ. Although not part of our initial study, this method could be considered in future investigations to corroborate the proteomic and spectroscopic data.

4) It is odd that the there is no Discussion section in the manuscript. If this a journal requirement? There is a brief Discussion section present on p. 14, but there is no heading identifying it as such. My preference would be to add a "Discussion" heading.

Response: We appreciate the suggestion. The presence of a formal "Discussion" heading is not a journal requirement, however we will add the "Discussion" heading in the revised manuscript to clearly separate the discussion of our findings from the results section.

5) The captions of Table 1 and Fig 4 are swapped.

Response: Thank you for pointing out the caption error. We will correct the captions for Table 1 and Figure 4 in the revised manuscript to avoid any confusion.

Reviewer #2 (Remarks to the Author):

Review of Manuscript: NCOMMS-24-50188

Title: Discovery of mammalian collagens I and III within ancient poriferan biopolymer spongin
Author(s): Ehrlich et al.

The leading author, Prof. Ehrlich gathered (again) a large panel of experts in various techniques, tools and analyses, to tackle a major question regarding the composition of sponge spongin components, and their relatedness to collagens of other animal phyla. The topic of the manuscript is therefore suitable to Nat. Comm. readership, but the content requires revision before accepted to be published. I have to emphasize that I read the entire paper including the supplementary material, but am qualified to expertly assess only some of the aspects reported here (as explained to the editor).

The complimentary techniques and approaches employed, led to the identification of collagen I & III in spongin fibers. In addition, specific cropsslinking agents were discovered, that also contribute to the understanding of the molecules function.

It is clear that the manuscript requires the input of an English (Scientific) writer/editor. Bellow I will mention only some examples.

Bellow I suggest some comments and present questions for the authors to address (in order of appearance – not importance).

Line 35 - "In other words, sponges are a point of origin for " Sponges diverged close to the origin of all other.

Response: Thank you for highlighting this point. We agree that the phrasing should be refined to reflect the evolutionary position of sponges more accurately. The sentence will be revised to: "In other words, sponges diverged early in the evolutionary history of multicellular animals, close to the origin of all other metazoans."

Line 42 - Delete "Keratosan sponges of".

Response: Thank you. We have implemented the changes in the manuscript.

Line 42 - Replace "are" with "containes".

Response: Thank you. We have implemented the changes in the manuscript.

Line 44 – Replace "skeletons of these poriferans are" with skeleton of sponges is"

Response: Thank you. We have implemented the changes in the manuscript.

Line 172 – I found no reference to the importance/meaning of the distribution as appears in Fig 3i.

Response: Thank you for pointing this. We have added the following literature references:

- Lefevre, T., Rousseau, M. E., & Pezolet, M. (2007). Protein secondary structure and orientation in silk as revealed by Raman spectromicroscopy. *Biophys J*, 92, 2885–2895.
- Church, J. S., Poole, A. J., & Woodhead, A. L. (2010). The Raman analysis of films cast from dissolved feather keratin. *Vibrational Spectroscopy*, 53, 107–111.

Line 182-184 - Proteomics is not "a method". It certainly is not aimed at "identifying corresponding collagen chains". This sentence should be rephrased.

Response: Thank you. We agree that the phrase needs refinement. We have revised the sentence to:

"Proteomics involves the application of various techniques to identify and characterize proteins, including the specific collagen chains present in diverse species of invertebrates and vertebrates."

Line 192 & Line 193 - The table and figure legends are mixed.

Response: Thank you for paying attention to that. We have corrected the legends for the table and figure in these lines to ensure they are properly aligned with their corresponding content.

Lines 229-232 - I disagree with the statement that in healthy sponge the spongin skeleton is exposed to seawater. Usually it is inside, covered by the mesohyl.

Response: We appreciate this correction. We agree that, in most cases, the spongin skeleton is indeed covered by mesohyl and not directly exposed to seawater. We have modified this statement to accurately reflect that the spongin skeleton is typically encased within the sponge's mesohyl matrix.

Lines 317-318 - I suggest to tune down this statement. The fact that collagens exist in sponges and other animals does not position sponges as the archaeal source of collagen(s), but that they all originated from the same root.

Response: Thank you for your comment. We have revised this statement to clarify that while sponges share a common evolutionary origin for collagen with other animals, they should not be positioned as the singular archaeal source of collagens.

Lines 330-331 - I really liked this concluding remark.

Response: Thank you for your positive feedback.

Between lines 338 and 490, no reference is mentioned to support, or describe, or justify, any of the methods used.

Response: Thank you for your comment. The references supporting those methods are majorly presented in the Supplementary Information section together with the results and figures. We have added information about that in the main text.

Line 399 - Do you mean "proteins ...... were" solubilized.."?

Response: Yes, we do. Thank you for pointing that. We have made corresponding changes to the paper.

Lines 529 - 626 - Ref 1 & 3-45 are without doi address, while the rest are with. Please decide.

Response: Thank you. We have made the changes to the manuscript.

Lines 564 & 569 - The same journal is cited twice differently.

Response: Thank you. We have made the necessary corrections.

Line 571 - Change to J. Spectros.

Response: Thank you. We have made the changes to the manuscript.

Line 586 - The name of the journal Nature, should not be abbreviated.

Response: Thank you. We have made the necessary corrections.

Line 601 - Why did the authors cite ref 42 in full (all 18 authors) while, ref 26, 43 and 44 (with 6, 8 and 7 authors respectively) are abbreviated to et al.

Response: Thank you for pointing that. We have made the necessary corrections.

Reviewer #3 (Remarks to the Author):

In their manuscript entitled „Discovery of mammalian collagens I and III within ancient poriferan biopolymer spongin", the authors Hermann Ehrlich, Markus Buehler et al., report on the structural composition of spongin, a skeletal compound of the first metazoan organisms. Using an integrated set of analytical techniques including vibrational spectroscopic, electron microscopy, NMR and combining it with molecular dynamics, the authors show that the biopolymer is composed of collagen I and III with dedicated crosslinking. This discovery is used to proposed some discussion on the origin of some collagen domains present in mammals in general and in humans in particular that are already found in this very ancient organism.

I have only minor points to note:

• Most of the labels present in figure 3 are simply not readable when depicted at 100% scale (so on a printed version). Please modify to make it readable.

Response: Thank you. We have updated the figure to make it more readable.

• The caption of Figure 4 appears on line 193, it should be on line 192 (directly after the figure, but before the table).

Response: We have made the corresponding changes. Thank you for the comment.

• Line 338, I think "imagery" should be "imaging".

Response: Thank you. We have made the necessary corrections.

• Line 421, liter is wrongly abbreviated with small "l" here, it is capital "L" as everywhere else in the manuscript.

Response: Thank you. We have made the necessary corrections.

• Article or page numbers are missing in the reference section.

Response: Thank you. We have made the necessary corrections.

• Fig SI 3.1, please modify the scale bar to 100 nm instead of 0.1 µm for consistency.

Response: We have modified the scalebar on the mentioned figure. Thank you for pointing that.

• Fig. SI 7.2, the scale bar is present, but no indication of the scale is given.

Response: Thank you for your attention to the details. We have added the information about the scalebar in the caption of the figure.

Looking forward to have this manuscript published
Damien Faivre

Reviewer #4 (Remarks to the Author):

This manuscript seeks to answer fundamental questions about spongin chemical and material nature using a broad range of molecular and chemical tools. The paper presents a thorough investigation that identified collagens I and III as the main structural components of spongin fibers using proteomics, Western blot, translated nucleotide BLAST method, C 13 solid state NMR, and Raman spectroscopy. The complex interplay of collagen molecular structures and crosslinks was characterized using molecular dynamic modeling based upon the HPLC-MS analysis of spongin collagen samples. The work is impressive and answers fundamental questions about collagen structure and crosslinking in spongin particularly but provides a fundamental basis for collagen chemistry and material properties. Prior to publication in Nature Communications, this work requires some modifications.

Response: We apprecuate your critical comments and remarks!

Please see the major and minor comments:

Major Comments:

1. The data in Figure 4 and Table 1 as presented is confusing. It is unclear what various columns in Table 1 are referring to, particularly the columns titled, ‘Scores’ and SC (%). The data in Figure 4, includes the SDS-Page data of the 3 different collagen proteins listed in the Table 1. It is not clearly mentioned but the 210723 A band seems to be a dimerization of both the collagen alpha-1(I) chain isoform X1 [Sus scrofa] and the Alpha 2 chain of type I collagen [Sus scrofa domesticus] whereas the other bands B & C are not dimers based on the reported molecular weights. In panels (a), (b), and (c) of Figure 4, individual bands are reported to be labeled with collagen I and collagen III antibodies based on different preparation methods of disintegrated spongin samples. The molecular weights of the labeled bands are much different than typically reported molecular weights for collagens I and III and do not correspond to the molecular weights provided in Table 1. Please increase clarity regarding this figure and its role in supporting your conclusions.

Response:

Thank you for your valuable feedback. Below is a detailed explanation addressing your comments regarding Table 1 and Figure 4:We also made corresponding changes in the test of the manuscript.

1. **Clarification of Table 1 Headers:**

   In the revised manuscript, we have added a detailed explanation of the column headers to the table’s caption for improved clarity:

   - **SDS PAGE BAND**: The band excised from the gel and subjected to analysis.
   - **Accession**: Database accession number of the identified protein.
   - **Protein**: Name of the identified protein.
   - **MW**: Molecular weight of the native protein, as provided by theoretical database data.
   - **Scores**: A statistical score indicating the probability of peptide or protein matching to the theoretical sequence in the database. A higher score corresponds to greater confidence in the identification.
   - **Peptides**: Number of peptides derived from the analyzed protein and mapped to the theoretical sequence.

- **S.C. (Sequence Coverage)**: Percentage of the protein sequence covered by the identified peptides.

The table presents results of the MS analysis of three bands excised from the gel (210723 A, 210723 B, 210723 C). For each band two results, with highest Scores, were shown, indicating varying levels of identification confidence. The higher the score, the greater the probability of correct identification. The data was compiled based on reports provided by Professor Miksik, who has detailed expertise on the subject. However, to ensure transparency and unambiguity of the results and avoid misunderstandings, we have removed identifications with lower "score" values, leaving only the most probable identifications in the table.

2. **Addressing SDS-PAGE Results:**

- It should be noted that the protein was extracted from a spongin biopolymer, and the identified fragments may exhibit molecular weights different from native collagen chains due to the extraction method and/or interspecies differences..
- The molecular weights listed in the table are theoretical values sourced from the database. These discrepancies are likely a result of degradation, processing conditions, or post-translational modifications of collagen in the spongin sample.

3. **Dimerization and Identification of Band A:**

- While we cannot unequivocally confirm that band A represents a dimer, MS analysis indicated that peptides derived from hydrolysis of band A cover 23.7% of the collagen alpha-1 sequence. This high sequence homology suggests that band A is likely related to collagen alpha-1, though we lack sufficient evidence to conclusively identify it as such.

4. **Western Blot Results:**

To confirm its collagen identity, we performed a Western blot. Antibodies designed against specific epitopes allow for precise identification of target proteins.

- The Western blot results show bands with lower molecular weights than native collagen chains. This may indicate the presence of lower molecular weight collagen fragments in addition to higher molecular weight collagens (210723 A, 210723 B, 210723 C).
- The antibodies used in Western blotting were generated against specific collagen fragments, Western blotting is detection method is **highly specific** strongly supporting our discovery..

Antigen information

Coll I A synthetic peptide corresponding to a sequence at the C-terminus of mouse Collagen I, identical to the related rat sequence, and different from the related human sequence by two amino acids.

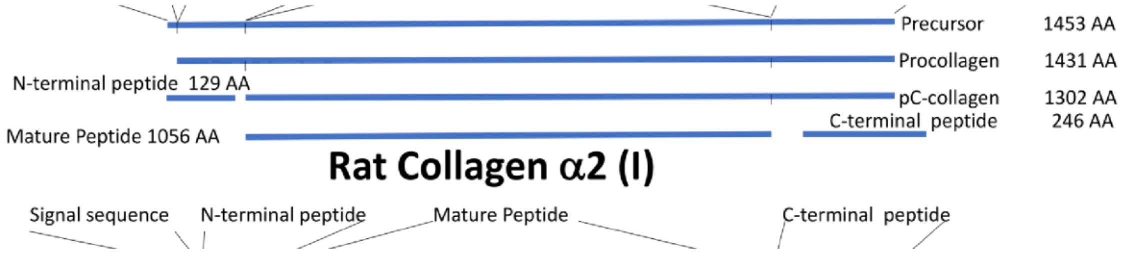

(C terminal peptide is approx. 30 kDa)

Coll III Recombinant protein fragment corresponding to a region within amino acids 1180 and 1444 of Human COL3A1

Seq. from Uniprot 1232-1466 aa Fibrillar collagen NC1

Sequence: DEIMTSLKSVNGQIESLISPDGSRKNPARNCRDLKFCHPELKSGEYWVDPNQGCK
LDAIKVFCNMETGETCISANPLNVPRKHWWTDSSAEKKHVWFGESMDGGFQFSYGNPELP
EDVLDVHLAFLRLLSSRASQNITYHCKNSIAYMDQASGNVKKALKLMGSNEGEFKAEGNS
KFTYTVLEDGCTKHTGEWSKTVFEYRTRKAVRLPIVDIAPYDIGGPDQEFGVDVGPVCFL

The presented results illustrate two complementary approaches used for collagen protein identification. Both mass spectrometry and Western blot confirmed the presence of proteins homologous to collagen proteins by comparing the peptide sequences with those deposited in the database (in the case of MS) and demonstrating affinity for specific antibodies (WB).

Minor Comments:

1. Column 1 in Figure 6 which shows the 3D visualizations of the cross talks are color coded. Please add to the legend that the purple color represents the bromide atoms.

Response: Thank you for this comment. We have made the necessary corrections to the figure legend.

2. Line 341: What are the percentages of ethanol used in the series and for how long is the sample left in each ethanol solution?

Response: Thank you for the valuable comment. We have added to the manuscript following information to clarify this:

"Dehydration through graded ethanol series: ethanol 30% 2 x 20 min; 50% 2 x 15 min; 70% 3 x 15 min; 95% 3 x15 min; 100% 3 x20 min. Araldite inclusion: LMRSOL3® (LaboModderne, France) 2 x 20 min; LMRSOL3®: Araldite epoxy embedding media (Spi Supplies, USA) (1:1) for 1hr; Araldite 2 x 2 h."

3. Line 349: How long were the samples dried at 37C?

Response: The samples were dried at 37°C for 24 hours. We have updated the manuscript to include this detail in the methods. Thank you for the comment.

4. Line 387: How much spongin powder was added incrementally every 3 hours to get to the final concentration?

Response: We have added 25 mg of spongin powder incrementally every 3 hours until reaching the final concentration of 1%. We have updated the manuscript to clarify this procedural detail.

5. Line 403: What gel chemistry was used for the polyacrylamide gel?

Response: Thank you for your valuable question. We have added the following information to the manuscript:

20 µL for silver stain was electrophoresed. Electrophoresis was carried out on polyacrylamide gel: on 4% stacking gel (7.8 mL dH_2O; 1 mL 1M Tris-HCl pH 6.8; 1mL 40% ROTIPHORESE®Gel 40 (37.5:1); 200 µL 20% SDS; 32 µL 30% APS; 16 µL TEMED) and 7.5% resolving gel (7.1 mL dH_2O; 300 µL glycerine; 5.2 mL 1.5M Tris-HCl pH 8.8; 3 mL 40% ROTIPHORESE®Gel 40 (37.5:1); 400 µL 20% SDS; 40 µL 30% APS; 16 µL TEMED).

6. Line 404: What was the specific running buffer used?

Response: Thank you for your valuable question. We have added the following information to the manuscript:

The gel was run using Tris-Glycine/SDS running buffer at 90 V (stacking gel) and 160 V (resolving gel). To control protein separation, the marker (Roti® Mark Tricolor) was simultaneously run on SDS-PAGE with the samples.

7. Lines 467 & 474: What was the specific transfer buffer used?

Response: Thank you for the question. We have used:

Anode buffer: 60mM Tris, 40 mM CAPS, pH 9,6 15% MeOH

Cathode Buffer: 60mM Tris, 40 mM CAPS, pH 9,6 0,1% SDS

This information was added to the revised version of the manuscript.

8. More information on the instrument settings of TEM and FTIR imaging is needed.

Response: We appreciate the reviewers' attention to detail, so following additional information is included in the Methods section of the revised manuscript to ensure full transparency and reproducibility of the experiments.

TEM

Purified, acellular, mineral- and pigment-free skeletal spongin fibers of Hippospongia communis demosponge were placed in 30% ethanol for one day at room temperature (RT). They were then dehydrated at RT in an ethanol series embedded in Araldite (Sigma-Aldrich) epoxy embedding media according to the manufacturer's instructions. Ultrathin sections (60–80 nm) were cut with an Ultramicrotome PowerTome XL, equipped with a Drukkert 45° diamond knife, and contrasted with UranyLess (EMS) solution and lead citrate. TEM analysis of the ultrathin sections was carried out by the FEI Tecnai F30-G2 transmission electron microscope. The TEM works with Super-Twin lenses (Thermo Fisher, Eindhoven, NL) and with a field emission gun at an acceleration voltage of 300 kV. The point resolution amounts to 2.0 Å, and the information limit amounts to about 1.2 Å. The microscope is equipped with a wide-angle slow scan CCD camera (MultiScan, 2k×2k pixels; Gatan Inc., Pleasanton, CA, USA). The analyses of the TEM images were realized by means of the Digital Micrograph software (Gatan, USA). Selected TEM images (Figures 1e and f) were processed by Bragg filtering of the Fourier transform of high-resolution images.

FTIR

FTIR spectra were recorded using a Nicolet 210c spectrometer (Thermo Scientific, Waltham, USA). The spectral resolution was 4 cm^{-1}, and the spectra were collected in the range of 4000 to 400 cm^{-1}. We used attenuated total reflectance (ATR) mode for the characterization of spongin and collagen samples, ensuring non-destructive analysis of the materials.

Reviewer #5 (Remarks to the Author):

I co-reviewed this manuscript with one of the reviewers who provided the listed reports. This is part of the Nature Communications initiative to facilitate training in peer review and to provide appropriate recognition for Early Career Researchers who co-review manuscripts.

Response: We deeply appreciate your participation in the reviewing process.

Dear Referees,

Thank you very much for your critical comments and remarks!

Point-by-point response that addresses the reviewers comments:

Reviewer 1:
One recommendation I would like to make for future work is to explore Bromine NMR for the purpose of using brominated Tyrosine as a spectrally uncrowded probe of the molecular configuration and dynamics of the collagens. Bromine has two stable isotopes, Br-79 and Br-81, each with close to 50% natural abundance and similar NMR properties; there are pros and cons for using either isotope for specific tasks (e.g. collagen identification, cross-link geometry or molecular dynamics). I am not suggesting that this work should be performed for the present paper; rather, this is a suggestion for future work.

Response:

We thank the reviewer for this insightful suggestion. Although this is outside the scope of the present work, we agree that exploring Bromine NMR and brominated Tyrosine could provide a valuable approach to investigating collagen molecular configurations and dynamics in future studies.

Future work could explore Bromine NMR, leveraging brominated Tyrosine as a spectrally uncrowded probe for studying molecular configurations and dynamics of collagens. Bromine's two stable isotopes, Br-79 and Br-81, with their distinct yet complementary NMR properties, may offer a novel means to analyze collagen cross-link geometries, molecular dynamics, and identification with high specificity. Also, for the future, LC MS/MS (with the characteristic isotope patterns of both halogens in the MS) remains the method of choice for the separation and identification of the observed compounds, where possible supported by 1H, 13C and maybe 15N NMR.

Reviewer 2:
One minor correction yet to be made, is to put the Table caption above the table (unlike the figure legends legend).

Response:

Thank you for pointing out this oversight. We will revise the manuscript to place the Table caption above the table as per standard formatting conventions.

Reviewer 4:
There is still some additional details involving Figure 4 that will improve clarity. From the figure caption, it can be inferred that Figure 4a is collagen I while Figure 4c is collagen III. Figure 4b is less clear where the authors state "Identification of collagens I and III in this spongin with Western Blot is represented in Fig (b)." Thus, more clarification of antibodies used is needed for Figure 4b. Furthermore, the authors do not address why Sample A (spongin at pH 7.1) has stronger bands than Sample B (spongin at pH 2.3) in Figure 4a (collagen I) while the opposite is true in Figure 4c (collagen III).

Response:

Thank you very much for your insightful comment. We greatly appreciate the opportunity to clarify and improve the manuscript.

We must acknowledge that the caption for Figure 4 is not accurate. Specifically, Figures 4a and 4c represent Western blot results (Figure 4a shows results using the Collagen I antibody, while Figure 4c shows results using the Collagen III antibody). Figure 4b, however, depicts SDS-PAGE results, which illustrate the total protein content rather than the specific collagen proteins identified by the antibodies in Figures 4a and 4c. We agree with your observation and have decided to remove Figure 4b for improved clarity in the publication.

Regarding the differences in band intensity, it is important to note that the intensity may reflect either the amount of protein present or the affinity of the antibody to the protein. Additionally, Western blotting is a semi-quantitative method and is not designed for precise quantification. In this study, Western blotting was used to confirm the presence of Collagen I and III in the samples. These results, when combined with those from the SDS-PAGE and MS analysis, provide complementary evidence supporting our thesis.

We made corresponding corrections in the revised manuscript.

Thank you once again for your valuable feedback, which has allowed us to make these clarifications and improvements.
